# Quantifying the Acidification-Induced Shift of the Dimerization Equilibrium of PsbS

**DOI:** 10.1021/acs.jpclett.6c00463

**Published:** 2026-03-31

**Authors:** Sara Vitória, Nicoletta Liguori, Roberta Croce, António M. Baptista

**Affiliations:** † Instituto de Tecnologia Química e Biológica António Xavier, 50106 Universidade Nova de Lisboa, Oeiras 2780-157, Portugal; ‡ Department of Physics and Astronomy, Faculty of Sciences, 1190 Vrije Universiteit Amsterdam, De Boelelaan 1081, 1081 HV Amsterdam, The Netherlands

## Abstract

Plants protect themselves from excess light by activating NPQ, a process triggered in the thylakoid by the pH-sensitive membrane protein PsbS upon lumen acidification and protonation of lumen-exposed glutamates. However, how these protonation events are coupled to PsbS oligomerization and photoprotective function is not fully understood. The present study combines constant-pH molecular dynamics simulations of monomeric and dimeric PsbS with a thermodynamic linkage analysis, providing the first direct quantification of the pH-induced shift on the dimerization free energy and monomer fraction. Results indicate only a small acidification-induced shift of the equilibrium toward the monomer under physiological conditions. Analysis of residue-level free-energy contributions reveals key pH-sensitive residues previously found as functionally important in experiments. We further identify complex, pH-dependent protonation correlation networks, especially under acidic conditions, and long-range correlations across the membrane, consistent with communication between lumenal and stromal regions in both oligomeric states.

## Graphical abstract

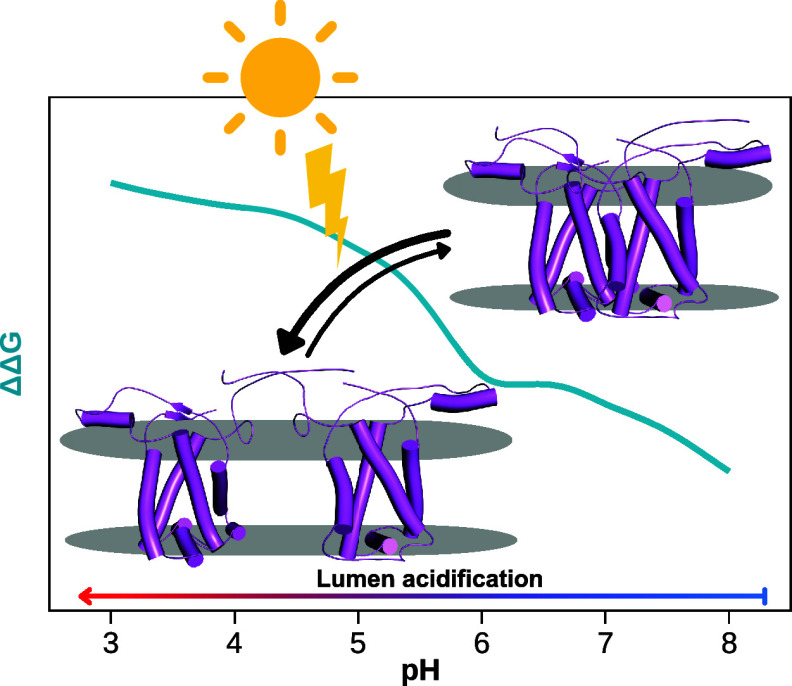

Photosynthesis is a fundamental biological process that converts solar energy into chemical energy. It takes place in the thylakoid membrane, where light-harvesting complexes (LHCs) efficiently funnel excitation energy to the photosynthetic reaction centers.^1^ Under high or fluctuating light, excess absorbed energy can promote the formation of reactive oxygen species, leading to photodamage.^2^ Plants mitigate this stress through photoprotective mechanisms such as nonphotochemical quenching (NPQ), which dissipates excess excitation energy as heat and protects photosystem II (PSII).^3–5^

Energy-dependent quenching (qE) is the fastest, most dynamic component of NPQ. It is rapidly triggered by lumen acidification (from ∼7.5 to ∼5.7) under high light^6^ and tunes light harvesting on time scales of seconds to minutes.^4,7^ A central player in qE is the Photosystem II subunit S (PsbS), a thylakoid membrane protein that functions as a pH sensor.^5^ This sensing arises from lumen-exposed glutamate residues with elevated p*K*_a_ values,^8–12^ enabling PsbS to respond to changes in lumen pH. Modulating PsbS abundance has therefore been reported as a route to improve tolerance to fluctuating light and enhance photosynthetic efficiency.^13–17^

PsbS is a single-chain 22 kDa member of the LHC superfamily^5,18,19^ but is structurally distinct from canonical LHC proteins, comprising four transmembrane helices (TM1–TM4; Figure 1) rather than three.^18^ On the lumenal side, three amphipathic helices (H1–H3) are embedded within loop regions. In the crystal structure, the dimer interface is stabilized by hydrogen bonds between Glu173 (on H2) and the backbone amide nitrogens of Ile74 and Tyr75 on the opposing partner (on H3).^18^ Site-directed mutagenesis identified the lumen-exposed glutamates Glu69 and Glu173 as critical for qE activation: mutations at these positions strongly reduce or abolish fast qE.^11,20,21^ Mutations of Glu55 and Glu159 also markedly impair qE, underscoring the functional importance of these residues.^20^

**Figure 1:**
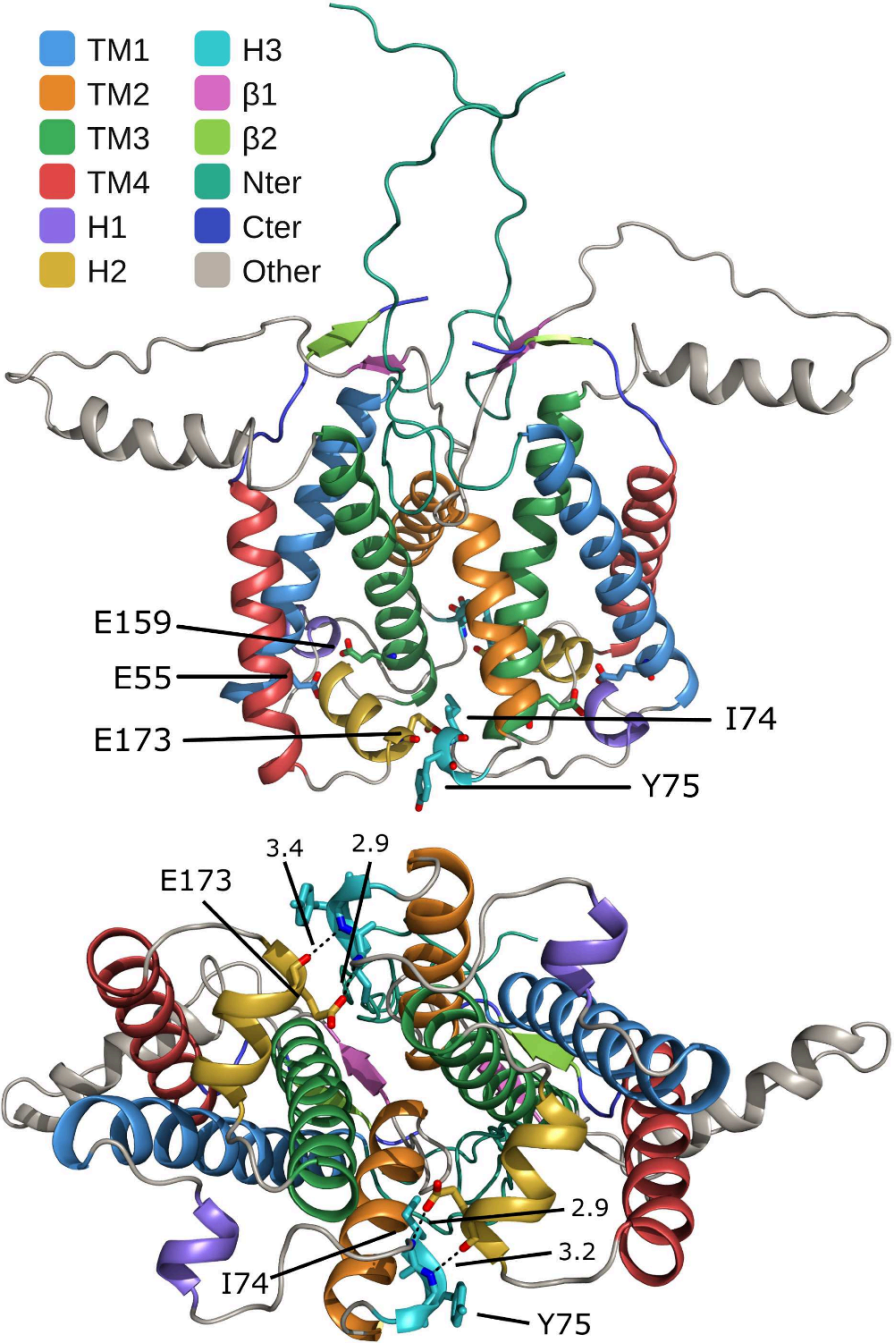
Crystal structure of the PsbS dimer,^18^ with the stromal loop segment (residues 108–133) rebuilt by sequence homology modeling using SWISS-MODEL.^22^ Top: Across-membrane view of the dimer, showing the different domains: transmembrane helices (TM1–TM4), the three lumenal helices (H1–H3), the two β-strands (B1–B2), and the N-terminal and C-terminal ends. Glu55 and Glu159, located in the lumenal loops, anchor helices H2 and H1 to TM1 and TM3, respectively. Bottom: Lumenal interface view showing the dimer-stabilizing hydrogen bonds Ile74–Glu173 and Tyr75–Glu173 formed between the two chains, with intermolecular distances indicated in ångströms.

The molecular determinants of PsbS activation have been widely studied in recent years,^8–10,21^ but important elements of the mechanism remain unresolved. The prevailing model is that excess light leads to acidification of the thylakoid lumen, triggering protonation of specific glutamate residues in PsbS. This protonation is proposed to promote a transition from an inactive dimeric state to an active monomeric state, enabling PsbS to activate qE, likely through interactions with LHC proteins or PSII.^23–28^ The underlying mechanism may involve two of the lumenal small helices, with recent studies^8–10^ showing that neutral pH tends to favor unfolding of the 3_10_-helical structure of H3 and tilting of H2 from the hydrophobic membrane phase toward the aqueous environment.

However, different experimental approaches have led to contrasting views of the pH dependence of the PsbS dimer–monomer equilibrium: SDS-PAGE under denaturing conditions suggests monomerization at low pH,^23^ whereas structural,^18^ spectroscopic,^10^ and computational^8,9^ studies indicate a stable dimeric state under these conditions.

This discrepancy highlights a fundamental gap in our understanding of how pH-dependent protonation events in PsbS are coupled to its oligomeric state, which is part of the broader issue of the effect of pH on protein association and binding.^29–31^ To address this issue, we use the stochastic titration method^32,33^ to perform constant-pH molecular dynamics simulations of the monomeric and dimeric forms of PsbS, in the pH range 3–8 to study the pH-dependence of the PsbS dimerization equilibrium. By analyzing the protonation behavior of key lumen-facing residues and its coupling to dimer stability through a thermodynamic linkage relation, following a recently introduced strategy,^34,35^ we intend to clarify how protonation equilibrium modulates PsbS dimerization. Details of the methodology are provided in the Supporting Information (SI).

Overall, PsbS remained structurally stable across the simulated pH range, exhibiting preserved secondary structure and noticeable fluctuations restricted to loop regions, with no large-scale rearrangements in terms of compactness or protein global tilt, nor substantial pH-dependency of interchain contact surface and hydrogen bonding (Figures S5–S10 in the SI), although some of these analyses are discussed below.

The movement of H2 into the aqueous phase at higher pH has been reported in experiments^10^ and 1 μs coarse-grained simulations,^9^ but not in shorter atomistic simulations,^8^ suggesting a slow, microsecond or longer, process. Despite an aggregate sampling time of 3 μs for each system (monomer and dimer) and pH value, this event was only rarely observed in our trajectories (illustrative structures for the internalized and exposed conformations are shown in Figure S13). We monitored the H2 tilt angle relative to the membrane normal and its possible relation to the protonation state of Glu173, and also measured the Glu173–Ile74 and Glu173–Tyr75 distances across chains (Figures S11 and S12 in the SI) to study potential changes in intermonomer contacts. Reorientation was observed only in a few trajectories (up to ∼160°), although no re-embedding occurred after solvent exposure, and its relation with Glu173 protonation was unclear. This prevents robust conclusions, but it indicates that this event is slow or rare but indeed possible. Further clarification of this question may require enhanced-sampling methods to increase the probability of capturing H2 reorientation events.

The secondary structure of helix H3 was quantified as the relative frequency of 3_10_-helix versus nonhelical states for the monomer and each chain of the dimer across the different pH conditions (Figure 2). Overall, the results show a pH-dependent shift from a 3_10_-helix toward more disordered conformations, consistent with reports of H3 destabilization under neutral conditions in both the monomer and dimer;^8–10^ the decrease is systematic, although within the statistical uncertainty. H3 is highly dynamic in most of the simulations, frequently interconverting between a 3_10_-helix and a turn, while in others, especially in the dimer, it tends to remain trapped in usually the nonhelical state (Figure S14 in the SI). Chain A exhibits a lower 3_10_-helix content than chain B, which retains higher helicity across conditions. However, by looking at Figure S14 this asymmetry seems to be a fortuitous consequence of the insufficient sampling due to the trapped simulations. This shows the importance of performing multiple simulations replicates.

**Figure 2:**
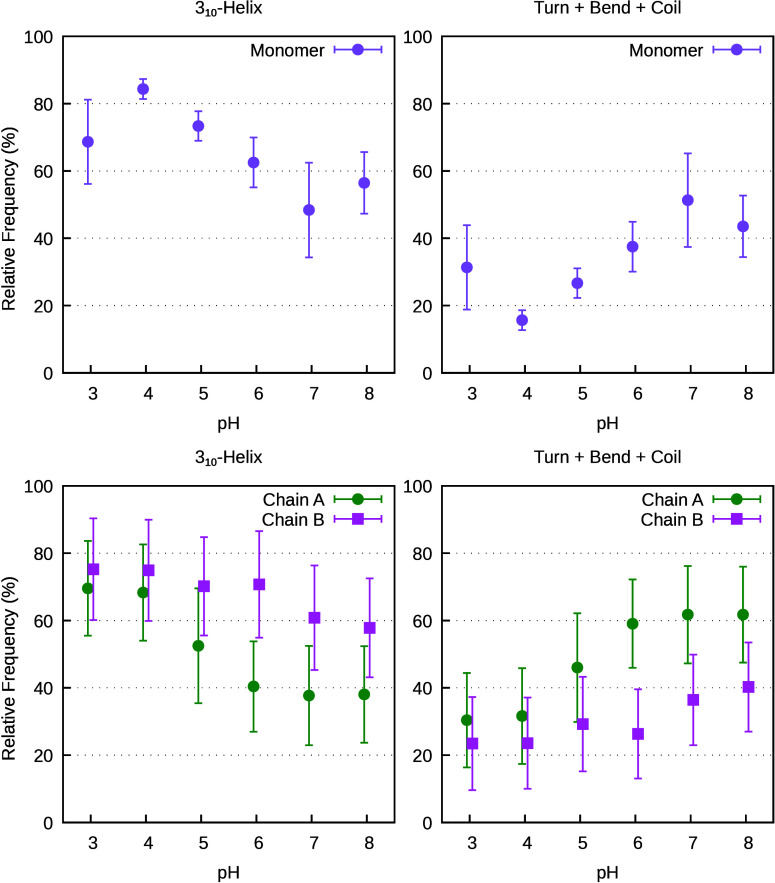
Secondary structure analysis of helix H3 in PsbS as a function of pH. The plots show the relative frequency of the 3_10_-helix and of disordered conformations (turn, bend, and coil) for the monomer and each chain in the dimer. Error bars represent the standard error of the mean across all replicates.

Global titration curves for the monomer and dimer are shown in Figure 3 (top). The dimer exhibits a systematically lower net charge across the pH range, likely driven by interactions at the dimer interface that stabilize the Asp/Glu charged forms. The separation between curves is largest at acidic pH and diminishes at high pH, where most residues are protonated and both curves approach zero net charge.

**Figure 3:**
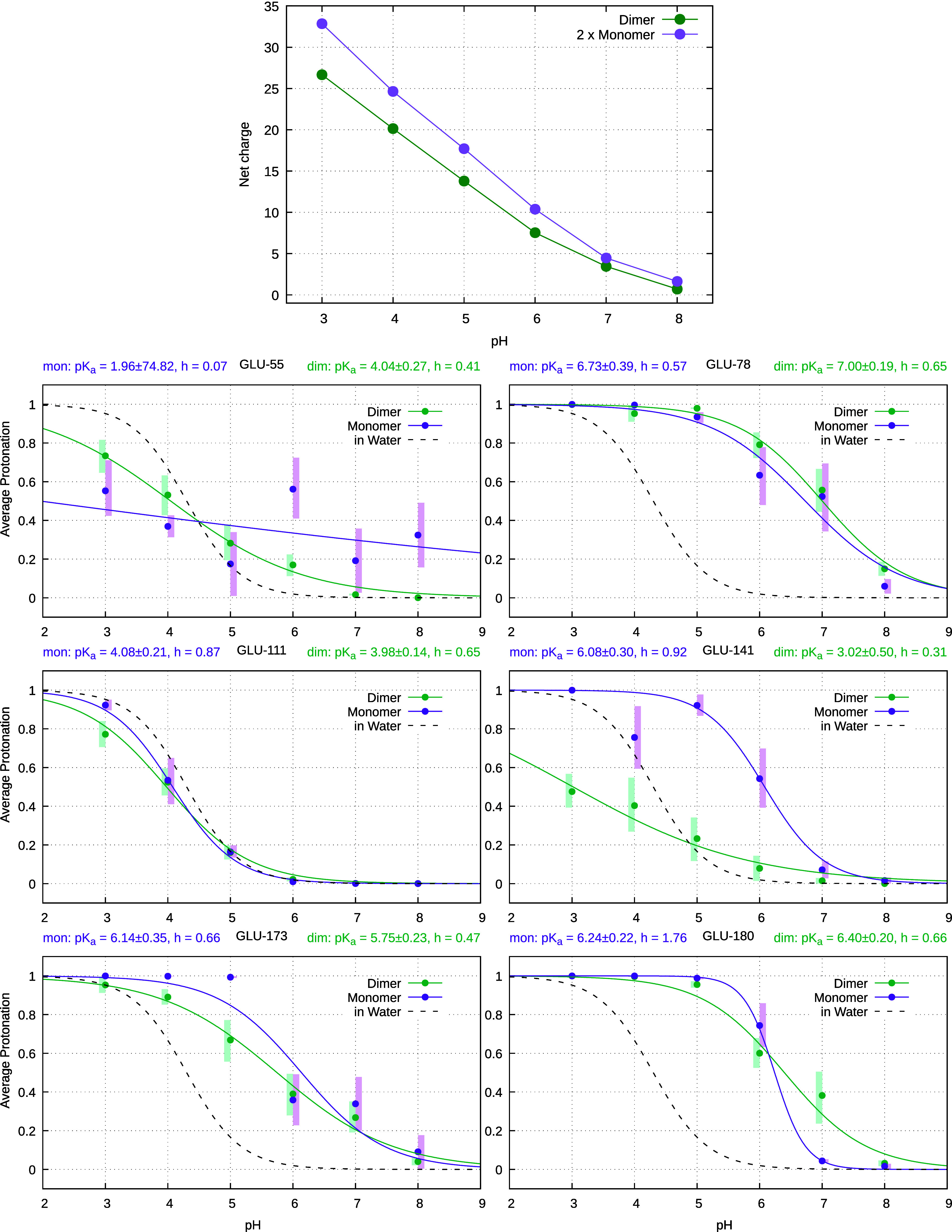
(Top) Global protonation curves for the dimer (green) and 2× monomers (purple) in order to match the dimer stoichiometry. Errors are all below 0.9. (Bottom) Titration curves of residues (Glu55, Glu78, Glu111, Glu141, Glu173, Glu180) in PsbS. The average protonation of each residue is shown for the monomer (purple) and dimer (average of both chains; green), with residues in water represented by black dashed curves. The titration curves were fitted to the Hill equation, and the fitted p*K*_a_ values (±error) and Hill coefficients (*h*) are indicated for each residue. The shaded transparent regions (purple and green) represent the error bounds, estimated as 68% confidence intervals obtained through the bootstrap method.

The computed p*K*_a_ values for individual residues (Table S1 in the SI) reveal important trends regarding the pH sensitivity of PsbS. In particular, several glutamates located on the lumenal side (e.g., Glu76, Glu78, Glu173, Glu180, and Glu182) have notable positive shifts in p*K*_a_ compared to the value in water (4.25),^36^ as it had already been reported by us and other independent studies.^8–10^ Given that their p*K*_a_ values are within 5–7, these lumen-facing residues were therefore suggested to play a direct role in sensing acidification and initiating the qE response. Additionally, some residues exhibit large negative Δp*K*_a_ values between monomer and dimer, such as Glu20, Asp21, Glu37, Asp98, Glu141, and Glu159. Although our computed p*K*_a_ values are broadly consistent with those reported for the PsbS monomer,^8^ the comparison with the dimer study^9^ reveals some discrepancies. The latter study reports no p*K*_a_ for Glu141 and seems to have treated Asp residues as nontitratable, presumably charged, thus ignoring the pH-dependent electrostatic effects that they may exert on Glu residues. Furthermore, its simulation time was 3-fold shorter than ours, which may have limited conformational exploration, such as alternative membrane embedding arrangements coupled to changes in protonation states. The use of different force fields and constant-pH MD algorithms may have also contributed to the discrepancies.

Some representative examples of different protonation behaviors are shown in Figure 3, both for lumenal (Glu55, Glu78, Glu173, Glu180) and stromal (Glu111, Glu141) residues; others are given in Figure S15 in the SI, which shows also the average protonation for each dimer chain. While some residue p*K*_a_ values remain relatively unaffected by dimerization (Glu78, Glu111, Glu173, Glu180), the titration curve of Glu180 becomes much more abrupt, indicating a transition from an anticooperative to cooperative binding, highlighting that the p*K*_a_ value alone is insufficient to fully describe protonation behavior. Glu141 exhibits the largest p*K*_a_ shift upon dimerization, despite being located away from the dimer interface, suggesting that long-range indirect effects may be at play, possibly mediated through networks of correlated acidic residues (see below). Glu55 displays an atypical and noisy titration curve in the monomer, making its fitted p*K*_a_ value merely indicative. This residue is located near two positive lysines, which seem to transiently trap its protonation state (see below).

Analyzing protonation correlations between residues can provide valuable insight into the identification of functional residues that participate in electrostatically driven mechanisms.^34,37^ Residue pairs with substantial correlations are shown in Figures S16 (monomer) and S17 (dimer) in the SI. Many strongly correlated residue pairs are present at pH 4–6, both in the dimer (involving Glu35, Glu37, Glu55, Glu141, Glu159, and Glu173) and the monomer (involving Glu20, Asp120, Glu141, and Glu159). Among these residues, Glu55, Glu159, and Glu173 are of particular interest, as mutation studies have identified them as critical for activating the qE component of NPQ, pointing to a link between protonation correlations and functional relevance,^11,20,21^ as observed in other studies.^34,35^ Correlated titratable residues give rise to a complex network of positive and negative couplings, shown in the correlation maps in Figures S18 and S19 in the SI. Interestingly, some correlations, mostly positive, occur between residues located on opposite sides of the membrane (Figures S20 and S21 in the SI). This pattern suggests a possible degree of stroma–lumen communication mediated by long-range electrostatic coupling across PsbS, as originally contemplated by Monod and co-workers when they introduced the concept of allostery, noting that it could be mediated by a simple “redistribution of charge” without noticeable structural changes.^38,39^ The presence of a complex and dynamic correlation network, involving both positive and negative correlations, shows the cooperative nature of proton sensing in PsbS and suggests that groups of residues, rather than isolated ones, participate in shaping the protein’s response to lumenal acidification.

The analysis of protonation correlation times (Figure S22 in the SI) shows high values up to 100 ns for several residues, indicating very slow proton exchange dynamics for both stromal (Glu35, Glu37, Glu105, Glu141) and lumenal (Glu55, Glu78, Glu159) residues. This may result in kinetically trapped protonations and associated poor sampling, likely explaining the large variability among replicates that gives rise to the substantial errors seen in the titration curves of these residues. As previously observed,^34,35^ the residues identified as being the most frequently involved in stronger correlations also tend to exchange protons at low rate, as reflected by the correlation times obtained for those residues. This suggests that extensive correlation networks can restrict the system to specific configurations of protonation states, thereby suppressing certain fluctuations and largely determining the protein charge distribution of the protein.

The global protonation curves of the monomer and dimer can be used to compute the pH dependence of the dimerization free energy, using the fact that the rate of change of a reaction free energy with pH is given by the difference between the protonation curves of products and reactants.^40,41^ Because it derives from a differential relation, this so-called “linkage relation” yields only relative free energies (section 1.4 in the SI), but it provides a useful way to avoid explicit free-energy calculations; thus, it has been applied to titration curves computed with rigid-structure models^42,43^ and with constant-pH MD.^34,44–46^ The relative dimerization free-energy profile computed for PsbS (top left of Figure 4) decreases with increasing pH, indicating that the PsbS dimer becomes progressively more stable under neutral conditions, although the differences within the physiological range (5.7–7.5)^6^ are small. This plot displays only the shift of the dimerization free energy *relative* to pH 3 (ΔΔ*G*), not the *absolute* free energy (Δ*G*). It is interesting that the pH-dependent pattern of the dimer contact surface (Figure S8 in SI) might seem to contradict the observed free energy trend; however, as shown before, a higher proximity between partners does not necessarily correspond to better binding.^34^

**Figure 4:**
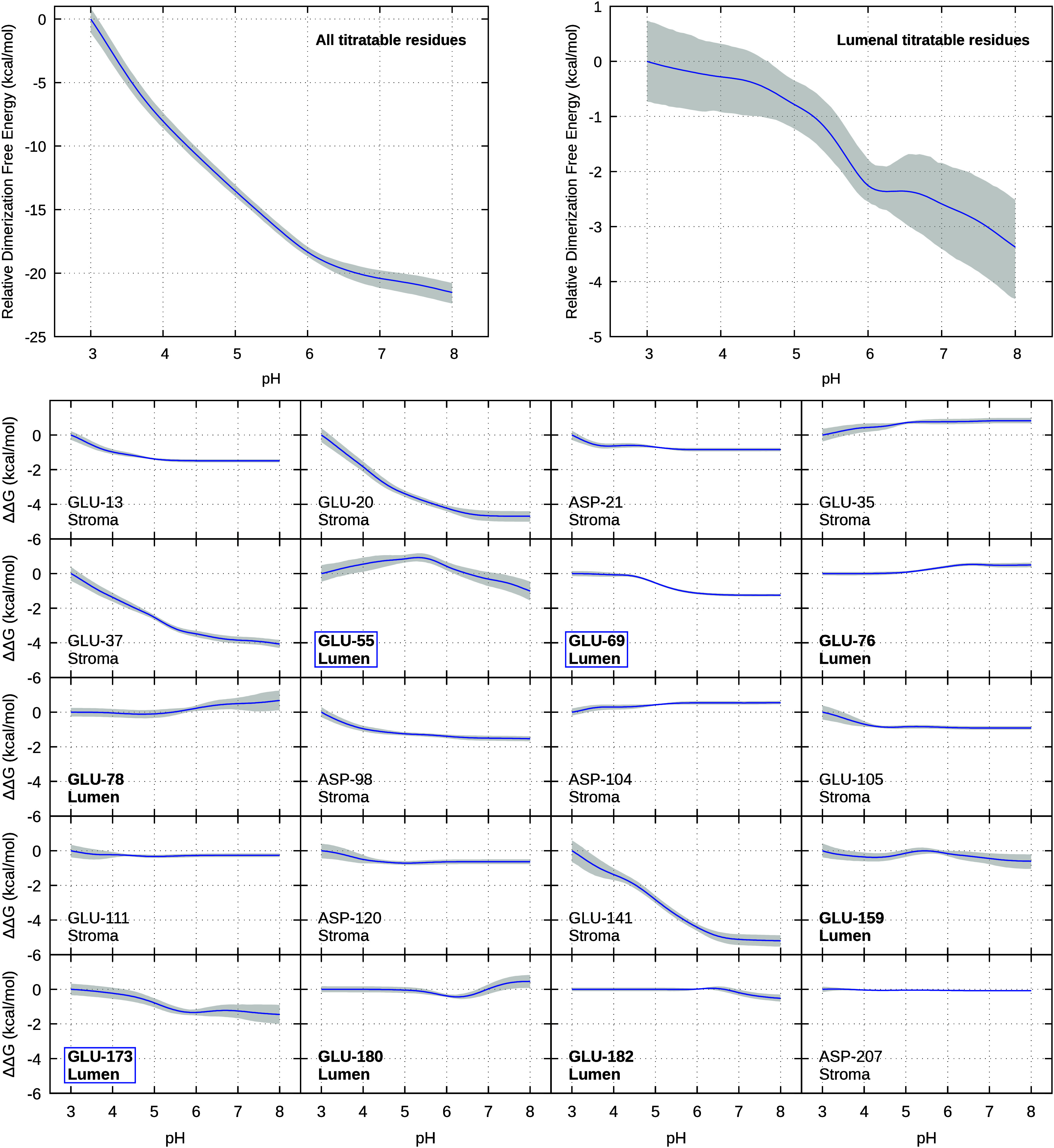
Relative dimerization free energy as a function of pH using pH 3 as reference is represented in blue considering all titratable residues (top left), only residues on the lumenal side (top right) and residue-specific contributions (bottom). In the bottom panel, lumenal residues are highlighted in bold, and Glu55, Glu69, and Glu173 are boxed in blue as they contribute most to the pH-dependent change in dimerization free energy. The shaded gray area corresponds to the error bounds, estimated as the 68% confidence interval obtained through the bootstrap method.

Because each titratable residue has a well-defined contribution to the global titration curve, the corresponding contributions to the relative dimerization free energy can be easily determined (section 1.4 in the SI). Most residues display largely flat contribution curves (bottom of Figure 4). This does not mean that these residues have a negligible contribution to the absolute Δ*G* but rather to the relative ΔΔ*G*, i.e., they have a negligible impact on the *pH sensitivity* of the Δ*G*; in fact, a residue may strongly contribute to Δ*G* and still show a flat ΔΔ*G* curve. The residues exhibiting the most pronounced pH-dependent behavior are Glu20, Glu37, and Glu141, all located on the stromal side. However, under physiological conditions the stromal side should remain largely unaffected, which is currently not possible with our simulation methodology. Nonetheless, we can focus only on the contributions of the lumenal residues (shown in bold in Figure 4). When using only the lumenal contributions, the total free energy retains a similar, although considerably less pronounced, decrease of dimerization with acidification (top right of Figure 4). Moreover, in this case the differences within the physiological pH range (5.7–7.5) are not only smaller but closer to the statistical uncertainties (which show a substantial increase beyond pH 6.2). The lumenal residues contributing at least ± 1 kcal/mol to this curve (boxed) are Glu55, Glu69, and Glu173, which are precisely three of the four residues that mutation studies have identified as critical for maintaining the qE component of NPQ;^11,20^ interestingly the missing one, Glu159, was captured by the correlation analysis shown above.

An alternative approach could have been to fix the stromal residues based on previously estimated p*K*_a_ values. However, this would have introduced its own inconsistencies, since those p*K*_a_ values were originally obtained with all residues treated as titratable, and the present study also yields different dimer p*K*_a_ values. Fixing stromal residues would therefore neglect the stroma–lumen protonation coupling that we observe, particularly at pH 7–8. By keeping the stromal residues titratable, we also retain a description of the no-gradient case, which is relevant for in vitro conditions. In any case, a pH gradient may not be strictly needed for activation, as a uniform pH decrease is sufficient to activate PsbS-dependent quenching in a minimal NPQ system using proteoliposomes.^47^

Turning the computed free energies into monomer fractions is nontrivial: the free energies are only relative, and self-association makes the result dependent on total PsbS concentration. However, if we assume the latter to be constant, it is possible to compute how the monomer fraction will vary if pH is changed (section 1.5 in the SI). Figure 5A presents the acidification-induced shift of the monomer fraction, showing that the monomer-to-dimer transition is less pronounced than often assumed. Nonetheless, the uncertainties associated with these calculations are substantial (plots B–F). Thus, lumen acidification alone may be sufficient to displace the equilibrium toward monomers, without invoking changes in total PsbS abundance, although this effect is small and somewhat uncertain.

**Figure 5:**
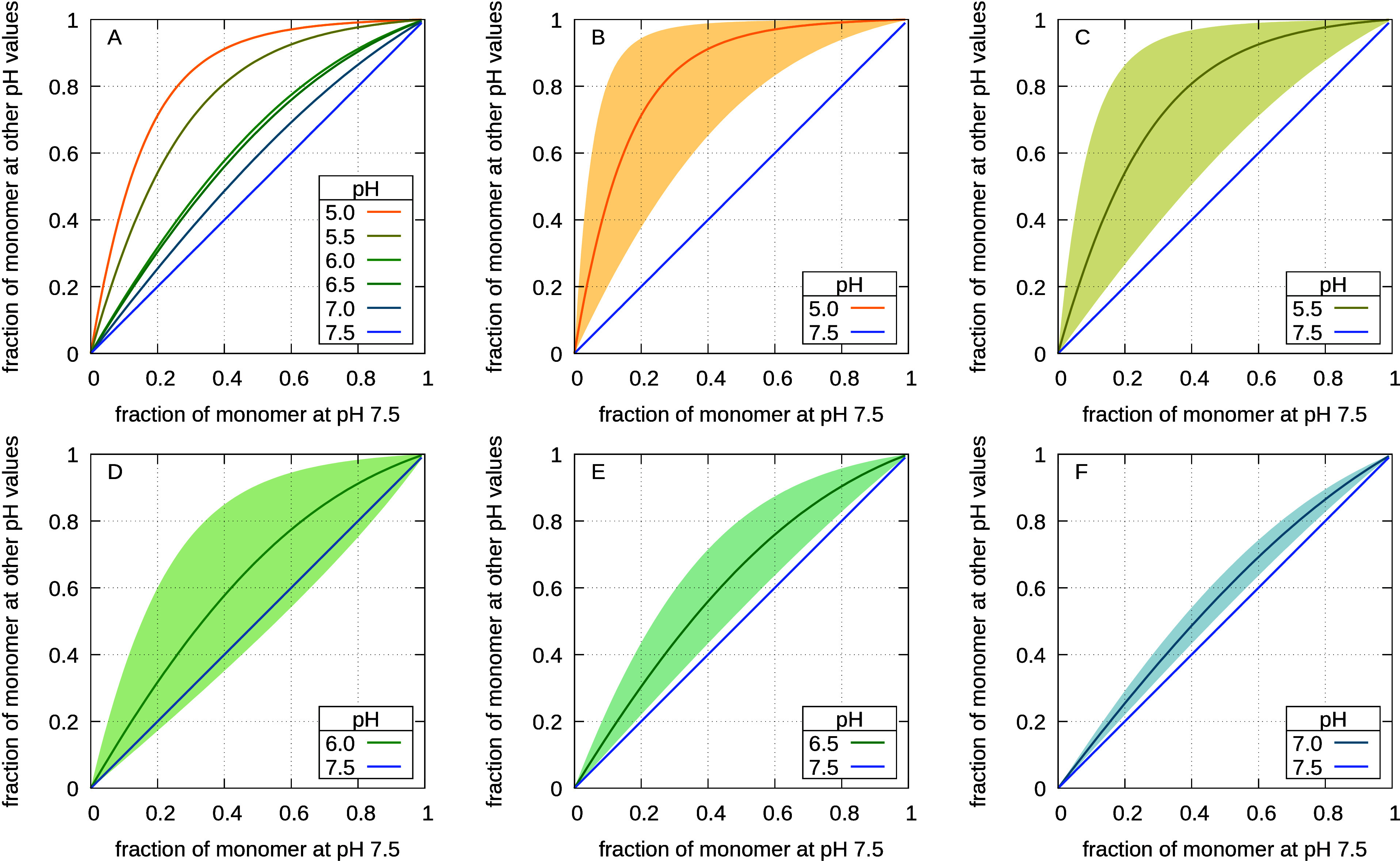
Effect of acidification on the pH-dependent fraction of PsbS present as monomer, relative to pH 7.5, calculated considering only the free-energy contributions of residues on the lumenal side. Panel (A) shows the curves at different pH values, while panels (B)–(F) show the associated statistical uncertainties for each one.

Our results contrast with the study of Chiariello et al., who suggested stronger stabilization of the dimer at low pH based on constant pH coarse-grained simulations combined with metadynamics-based potential of mean force calculations of the dimer dissociation.^9^ Nevertheless, it is important to note that their metadynamics simulations were performed with fixed protonation states, which assumes that PsbS has the same protonation states in both oligomeric forms, such that their titration curves are identical. However, if this were true, linkage function theory would imply that the dimerization free energy is pH-independent (see eq 1 in the SI). By allowing the protonations to freely adapt to the oligomerization form, our approach provides a more realistic depiction of the effect of pH on dimerization and, importantly, makes possible to compute the pH-dependent shift of not only the dimerization free energy, but also of the monomer fraction.

Other factors may affect the dimer–monomer equilibrium. In particular, lumen acidification also induces a shift of the xantophill cycle toward the production of zeaxanthin,^3–5^ whose presence was reported to weaken the interaction between the PsbS dimer partners in a molecular simulation study.^28^ This effect was not examined in the present study, nor in the previous simulation studies reporting a more stable dimer at low pH.^8,9^

In conclusion, the present study provides the first quantification of the pH-induced shift on the dimerization free energy and monomer fraction of PsbS. It gives some support to the view that lumen acidification promotes an increase of its monomeric form,^23,24,28^ but only moderately and affected by some uncertainty. Furthermore, we were able to identify pH-sensitive residues through their individual free-energy contributions, namely Glu55, Glu69, Glu173, which were experimentally identified as functionally important.^11,20^ We also observed protonation correlations in both the monomer and dimer that form complex pH-dependent networks, particularly under acidic conditions, as well as long-range correlations across the membrane, suggesting that communication between the stroma and the lumen may occur in both oligomeric states. Interestingly, the functionally important residue Glu159 ^20^ is found as one of the most correlated residues. Overall, this agreement with experimental observations reinforces the validity of the present methodology to study protein–protein association, combining free-energy calculations based on a linkage relation with protonation correlation analysis. More broadly, this integrated linkage-based free-energy framework with residue-resolved protonation-correlation analysis is readily transferable to other pH-responsive membrane proteins, offering general physical insight into how protonation reshapes association equilibria and long-range allosteric coupling.

## Supplementary Material



## Notes

Detailed methods and supplementary tables and figures addressing equilibration, structural analysis, protonation behavior and protonation correlations (PDF)

## References

[1] Blankenship, R. E. Molecular Mechanisms of Photosynthesis, 3rd Edition; John Wiley & Sons, Ltd.: Chichester, U.K., 2021.

[2] Apel K., Hirt H. (2004). Reactive oxygen
species: metabolism, oxidative stress, and signal
transduction. Annu. Rev. Plant Biol..

[3] Müller P., Li X.-P., Niyogi K. K. (2001). Non-photochemical
quenching. A response
to excess light energy. Plant Physiol..

[4] Ruban A. V., Johnson M. P., Duffy C. D. (2012). The photoprotective
molecular switch
in the photosystem II antenna. Biochim. Biophys.
Acta, Bioenerg..

[5] van Amerongen, H. ; Croce, R. Nonphotochemical quenching in plants: Mechanisms and mysteries. Plant Cell 2025, 37, 10.1093/plcell/koaf240. PMC12619064 41058045

[6] Takizawa K., Cruz J. A., Kanazawa A., Kramer D. M. (2007). The thylakoid proton
motive force in vivo. quantitative, non-invasive probes, energetics,
and regulatory consequences of light-induced pmf. Biochim. Biophys. Acta, Bioenerg..

[7] Horton P., Ruban A. V., Walters R. G. (1996). Regulation
of light harvesting in
green plants. Annu. Rev. Plant Biol..

[8] Liguori N., Campos S. R. R., Baptista A. M., Croce R. (2019). Molecular anatomy of
plant photoprotective switches: The sensitivity of PsbS to the environment,
residue by residue. J. Phys. Chem. Lett..

[9] Chiariello M. G., Grünewald F., Zarmiento-Garcia R., Marrink S. J. (2023). pH-dependent conformational
switch impacts stability of the PsbS dimer. J. Phys. Chem. Lett..

[10] Krishnan-Schmieden M., Konold P. E., Kennis J. T. M., Pandit A. (2021). The molecular
pH-response
mechanism of the plant light-stress sensor PsbS. Nat. Commun..

[11] Li X.-P., Gilmore A. M., Caffarri S., Bassi R., Golan T., Kramer D., Niyogi K. K. (2004). Regulation
of photosynthetic light
harvesting involves intrathylakoid lumen pH sensing by the PsbS protein. J. Biol. Chem..

[12] Li X.-P., Björkman O., Shih C., Grossman A. R., Rosenquist M., Jansson S., Niyogi K. K. (2000). A pigment-binding protein essential
for regulation of photosynthetic light harvesting. Nature.

[13] Murchie E. H., Niyogi K. K. (2011). Manipulation of photoprotection to improve plant photosynthesis. Plant Physiol..

[14] Głowacka K., Kromdijk J., Kucera K., Xie J., Cavanagh A. P., Leonelli L., Leakey A. D., Ort D. R., Niyogi K. K., Long S. P. (2018). Photosystem II subunit S overexpression increases the
efficiency of water use in a field-grown crop. Nat. Commun..

[15] Hubbart S., Smillie I. R. A., Heatley M., Swarup R., Foo C. C., Zhao L., Murchie E. H. (2018). Enhanced
thylakoid photoprotection
can increase yield and canopy radiation use efficiency in rice. Commun. Biol..

[16] Kromdijk J., Głowacka K., Leonelli L., Gabilly S. T., Iwai M., Niyogi K. K., Long S. P. (2016). Improving photosynthesis and crop
productivity by accelerating recovery from photoprotection. Science.

[17] De
Souza A. P., Burgess S. J., Doran L., Hansen J., Manukyan L., Maryn N., Gotarkar D., Leonelli L., Niyogi K. K., Long S. P. (2022). Soybean photosynthesis and crop yield
are improved by accelerating recovery from photoprotection. Science.

[18] Fan M., Li M., Liu Z., Cao P., Pan X., Zhang H., Zhao X., Zhang J., Chang W. (2015). Crystal structures
of the PsbS protein essential for photoprotection in plants. Nat. Struct. Mol. Biol..

[19] Marulanda
Valencia W., Pandit A. (2024). Photosystem II subunit S (PsbS):
A nano regulator of plant photosynthesis. J.
Mol. Biol..

[20] Li X.-P., Phippard A., Pasari J., Niyogi K. K. (2002). Structure–function
analysis of photosystem II subunit S (PsbS) in vivo. Funct. Plant Biol..

[21] Chen L., Rodriguez-Heredia M., Hanke G. T., Ruban A. V. (2025). Distinct features
of PsbS essential for mediating plant photoprotection. Plant Commun..

[22] Schwede T. (2003). Swiss-model:
an automated protein homology-modeling server. Nucleic Acids Res..

[23] Bergantino E., Segalla A., Brunetta A., Teardo E., Rigoni F., Giacometti G. M., Szabò I. (2003). Light- and pH-dependent structural
changes in the PsbS subunit of photosystem II. Proc. Natl. Acad. Sci. U. S. A..

[24] Correa-Galvis, V. ; Poschmann, G. ; Melzer, M. ; Stühler, K. ; Jahns, P. PsbS interactions involved in the activation of energy dissipation in arabidopsis. Nat. Plants 2016, 2, 10.1038/nplants.2015.225. 27249196

[25] Johnson M. P., Ruban A. V. (2011). Restoration of rapidly
reversible photoprotective energy
dissipation in the absence of PsbS protein by enhanced ΔpH. J. Biol. Chem..

[26] Holzwarth A. R., Miloslavina Y., Nilkens M., Jahns P. (2009). Identification of two
quenching sites active in the regulation of photosynthetic light-harvesting
studied by time-resolved fluorescence. Chem.
Phys. Lett..

[27] Kiss A. Z., Ruban A. V., Horton P. (2008). The PsbS protein controls the organization
of the photosystem II antenna in higher plant thylakoid membranes. J. Biol. Chem..

[28] Daskalakis V., Papadatos S. (2017). The photosystem II subunit S under stress. Biophys. J..

[29] Wallace J. A., Shen J. K. (2012). Unraveling a Trap-and-Trigger Mechanism
in the pH-Sensitive
Self-Assembly of Spider Silk Proteins. J. Phys.
Chem. Lett..

[30] Law S. M., Zhang B. W., Brooks C. L. (2013). pH-sensitive
residues in the p19 RNA silencing suppressor protein from carnation
It alian ringspot virus affect siRNA binding stability. Protein Sci..

[31] Onufriev A. V., Alexov E. (2013). Protonation and pk
changes in protein–ligand
binding. Q. Rev. Biophys..

[32] Baptista A. M., Teixeira V. H., Soares C. M. (2002). Constant-pH molecular
dynamics using
stochastic titration. J. Chem. Phys..

[33] Machuqueiro M., Baptista A. M. (2006). Constant-pH molecular
dynamics with ionic strength
effects: Protonation-conformation coupling in decalysine. J. Phys. Chem. B.

[34] da
Rocha L., Baptista A. M., Campos S. R. R. (2022). Approach to study
pH-dependent protein association using constant-pH molecular dynamics:
Application to the dimerization of β-lactoglobulin. J. Chem. Theory Comput..

[35] da
Rocha L., Campos S. R. R., Baptista A. M. (2025). Computing the pH-dependent
thermodynamics of the allostery between dimerization and palmitate
binding in β-lactoglobulin. J. Phys. Chem.
B.

[36] Grimsley G. R., Scholtz J. M., Pace C. N. (2009). A summary
of the measured pK values
of the ionizable groups in folded proteins. Protein Sci..

[37] Baptista A. M., Martel P. J., Soares C. M. (1999). Simulation
of electron-proton coupling
with a Monte Carlo method: Application to cytochrome *c*
_3_ using continuum electrostatics. Biophys. J..

[38] Monod J., Changeux J.-P., Jacob F. (1963). Allosteric proteins and cellular
control systems. J. Mol. Biol..

[39] Monod J., Wyman J., Changeux J.-P. (1965). On the
nature of allosteric transitions:
A plausible model. J. Mol. Biol..

[40] Wyman, J. ; Gill, S. J. Binding and Linkage: Functional Chemistry of Biological Macromolecules; University Science Books: Mill Valley, CA, 1990.

[41] Tanford, C. Protein denaturation: Part C. Theoretical models for the mechanism of denaturation. In Advances in Protein Chemistry; Elsevier, 1970; p 1. 4912353

[42] Yang A.-S., Honig B. (1993). On the ph dependence of protein stability. J. Mol. Biol..

[43] Schaefer M., Sommer M., Karplus M. (1997). ph-dependence of protein stability:
Absolute electrostatic free energy differences between conformations. J. Phys. Chem. B.

[44] Wallace J. A., Shen J. K. (2012). Unraveling a trap-and-trigger mechanism in the pH-sensitive
self-assembly of spider silk proteins. J. Phys.
Chem. Lett..

[45] Law S. M., Zhang B.-W., Brooks C. L. (2013). pH-sensitive
residues in the p19
RNA silencing suppressor protein from carnation italian ringspot virus
affect siRNA binding stability. Protein Sci..

[46] Kim M. O., McCammon J. A. (2016). Computation of pH-dependent
binding free energies. Biopolymers.

[47] Nicol L., Croce R. (2021). The PsbS protein and
low pH are necessary and sufficient to induce
quenching in the light-harvesting complex of plants LHCII. Sci. Rep..

