# Supplementary material for: Quantifying the Acidification-Induced Shift of the Dimerization Equilibrium of PsbS
Source: J Phys Chem Lett. 2026 Mar 31;17(14):4063–70. doi: 10.1021/acs.jpclett.6c00463 (PMC13071914; doi:10.1021/acs.jpclett.6c00463)
Supplement: Supplementary file 1 [file jz6c00463_si_001.pdf]

Supporting Information: Quantifying the Acidification-Induced Shift of the Dimerization Equilibrium of PsbS

Sara Vitória[1,†] Nicoletta Liguori[2,†] Roberta Croce[2,†] and António M. Baptista[1*,†]

†(1) *Instituto de Tecnologia Química e Biológica António Xavier, Universidade Nova de Lisboa, Oeiras, Portugal*

‡(2) *Department of Physics and Astronomy, Faculty of Sciences, Vrije Universiteit Amsterdam, De Boelelaan 1081, 1081 HV Amsterdam, The Netherlands*

E-mail: baptista@itqb.unl.pt

Contents

1 Methods **S-2**

1.1 System Preparation S-2

1.2 Molecular Mechanics (MM) and Molecular Dynamics (MD) Settings S-3

1.3 Constant-pH MD Simulations S-4

1.4 Relative Dimerization Free Energy S-6

1.5 Fraction of Monomers S-8

1.6 Other Analysis S-9

2 Equilibration **S-12**

3 Structural Analysis S-36

4 Protonation Behaviour S-49

5 Protonation Correlations S-54

References S-61

1 Methods

1.1 System Preparation

The crystal structure of the PsbS[S1] (PDB entry 4ri2)[S2] was used as the starting point for all simulations. However, several amino acid residues were missing in the experimental structure: five residues at the N-terminus, four at the C-terminus, and a relatively large portion of the stromal loop (residues 108 to 133).[S1] As the latter region is essential for connecting transmembrane helices TM2 and TM3, it was reconstructed using sequence homology modelling via the SWISS-MODEL server.[S3] The reconstructed PsbS was embedded into a POPC lipid bilayer solvated in water that was pre-equilibrated for 1 μs. To eliminate steric clashes, lipid molecules located within 1.5 Å and water molecules within 2 Å of the protein were removed using VMD.[S4] The system was energy minimized (50 thousand steepest descent steps), then equilibrated in two phases: 10 ps in NVT ensemble and 40 ns in the NPT ensemble. During equilibration, isotropic position restraints of 200 kJ mol^{-1} nm^{-2} were applied to all protein atoms to preserve the structural integrity of PsbS. Simulations were carried out with the GROMOS 54A7 force field for the protein,[S5] the SPC water model[S6] and Kukol et. al force field for POPC.[S7] In the MD setup, NPT simulations were performed with a 2 fs timestep, with all bonds constrained by the LINCS algorithm.[S8] Long-range electrostatics were handled using PME,[S9] with cutoffs of 1 nm for Coulomb and 1.4 nm for van der Waals interactions. Semi-isotropic pressure coupling was applied with a target pressure

of 1 bar using the Parrinelo-Rahman barostat[S10] ($\tau = 5$ ps). Temperature was maintained at 300 K via a Nose-Hoover thermostat[S11] ($\tau = 0.5$ ps). Solvent, membrane, and protein were independently coupled to the thermostat. Periodic boundary conditions were applied in all directions. This protocol was applied to both the monomer and dimer. The monomer system contained 259 POPC molecules and approximately 26,800 water molecules, whereas the final simulation box of the dimer contained 305 POPC lipids and approximately 34,200 water molecules. The numbers of Na^+ and Cl^- ions were determined by requiring that the simulation box is electrically neutral and has an ionic strength of 0.1 M.[S12] The precise ratio of ions was determined empirically by performing preliminary constant-pH MD simulations for each pH value and simulation replicate. The placement of ions was done randomly and performed using the `genion` tool in GROMACS.

1.2 Molecular Mechanics (MM) and Molecular Dynamics (MD) Settings

After system preparation, all subsequent simulations were run using the GROMACS simulation package version 2023.2.[S13] Constraints were imposed to all bonds using the LINCS algorithm[S8] for protein atoms and the SETTLE algorithm for water molecules.[S14] The PME method[S9] was employed to handle long-range electrostatic interactions during the MM/MD simulations. A short-range cut-off of 1.0 nm was applied to both Coulombic and van der Waals interactions, with long-range contributions treated with PME using a Fourier grid spacing of 0.16 nm and an interpolation order of 4. Neighbor lists were constructed using the Verlet algorithm[S15] and updated every 10 steps. The Leap-Frog integrator[S16] was employed to solve Newton's equations of motion, using a 2 fs time step throughout the simulations.

A three-step minimization procedure was performed, that started with 50 ps of molecular MD simulation in the NVT ensemble, using a temperature coupling constant $\tau_T = 1$ ps and applying harmonic restraints to all atoms with a force constant of 1000 kJ mol^{-1} nm^{-2}. This

was followed by another 50 ps of NVT simulation under the same temperature coupling, but with restraints applied only to the Cα atoms. Finally, 100 ps of MD was performed in the NPT ensemble with pressure coupling using the semiisotropic Berendsen barostat ($\tau_P = 5$ ps),[S17] maintaining restraints only on the Cα atoms. The temperature was set to 300 K and the pressure at 1 atm.

1.3 Constant-pH MD Simulations

Constant-pH MD (CpHMD) simulations were performed using the stochastic titration method developed by Baptista and co-workers.[S18,S19] This methodology allows proper sampling of conformational and protonation states by alternating between MM/MD simulations and Poisson-Boltzmann (PB) and Monte Carlo (MC) calculations to update the protonation states. All CpHMD simulations were performed for both the monomer and dimer at six different pH values: 3, 4, 5, 6, 7, and 8. These pH conditions were chosen to capture the pH variations that occur in the thylakoid lumen during the NPQ process. To ensure statistical significance, six independent replicates were performed for each pH condition. CpHMD simulations were performed with 500 ns per pH value and replicate, resulting in a total of 36 CpHMD simulations and an aggregate simulation time of 18 μs for each system. The reduced titration approach[S19] was employed with a threshold of 0.001, where an exclusion list was generated and updated every 50 CpHMD cycles. Each titration cycle consisted of 0.2 ps of solvent relaxation MM/MD, followed by a 10 ps MM/MD simulation of the entire system. All molecular MM/MD simulations were run in the NPT ensemble, maintaining constant pressure and temperature. The system temperature was set to 300 K and the pressure to 1 atm, both regulated using a separate thermostat v-rescale[S20] and barostat Parrinello-Rahman[S10] couplings with relaxation times $\tau_T = 1$ ps and $\tau_P = 5$ ps, respectively.

Arg, Lys and Tyr residues were treated as permanently protonated, as indicated by preliminary PB/MC rigid-structure calculations on the structures obtained after the standard MD equilibrations, and also based on our previous CpHMD study of the monomer[S21] (all

with $\mathrm{p}K_\mathrm{a} > 10$). The N- and C-termini were considered neutral due to the absence of the final residues in the protein sequence (see above). Proton tautomerism in the syn conformation was applied to all other titratable residues, namely Asp and Glu.[S22] The parametrization of the model compounds developed for the GROMOS 54A7 force field by Carvalheda et al.[S23] was used. However, since only the proton oriented towards the front was considered, the $\mathrm{p}K_\mathrm{a}$ of each tautomeric form was corrected using $\mathrm{p}K_\mathrm{taut} = \mathrm{p}K_\mathrm{mod} - \log 2$ to account for this effect.

In addition, because long-range electrostatic interactions were treated using PME rather than GRF approach employed in the original parametrization of the model compounds, control simulations were performed using a pentapeptide system (Ac-$\mathrm{Ala_2}$-X-$\mathrm{Ala_2}$-$\mathrm{NH_2}$, where Ac denotes acetyl and X represents either Asp or Glu). This peptide provides a protein-like chemical environment suitable for the selected model fragments. The simulations were carried out using the same parameters as those used for the PsbS system in order to assess whether the parametrization remained valid under our simulation conditions. The results of these control simulations indicated that the GROMOS 54A7 parametrization of the model compounds by Carvalheda et al.[S23] is appropriate, as the protonation fractions obtained from our simulations closely matched those expected from the experimental $\mathrm{p}K_\mathrm{a}$ values reported by Grimsley et al.[S24]

PB calculations were done using the MEAD package (version 2.2.9)[S25] and consisted of finite difference calculations using a three-step approach, the initial grid was defined as a cube of $121\times121\times121$ points with a mesh size of 1.5 Å centered on the entire system. This was followed by a second focusing step with an $81\times81\times81$ grid at 1 Å spacing, and finally a high-resolution grid of $81\times81\times81$ points with 0.25 Å spacing. The last two grids were centered on the titratable groups. Atomic charges and radii were assigned based on the GROMOS 54A7 force field,[S13] as described before.[S26] The molecular surface was constructed using a solvent probe with a radius of 1.4 Å and a Stern layer of 2.0 Å was applied to account for ion exclusion near the surface. The dielectric constant was set to 2 for the protein interior and 80 for the

solvent environment. Calculations were performed at a temperature of 300 K and an ionic strength of 0.1 M. The protonation state sampling was carried out using MC simulations as implemented in version 1.6 of the in-house PETIT software.[S22] It was performed 10^5 MC cycles and each one involved random selection of protonation states, with transitions accepted or rejected according to the Metropolis criterion,[S27] allowing convergence toward the equilibrium distribution of protonation microstates.

1.4 Relative Dimerization Free Energy

From CpHMD simulations, it is possible to determine the average protonation states of both monomeric and dimeric species across a range of pH values. According to the linkage function theory[S12,S28,S29] these data permits the calculation of the relative dimerization free energy at any pH given by Equation 1:

$$\Delta\Delta G^{\circ}(\mathrm{pH}) = \Delta G^{\circ}(\mathrm{pH}) - \Delta G^{\circ}(\mathrm{pH_{ref}}) = \ln(10)\, RT \int_{\mathrm{pH_{ref}}}^{\mathrm{pH}} \left(\langle n\rangle^{\mathrm{D}} - 2\langle n\rangle^{\mathrm{M}}\right)\, d\mathrm{pH}' \tag{1}$$

In this expression, $\langle n\rangle^{\mathrm{D}}$ and $\langle n\rangle^{\mathrm{M}}$ are the average number of titratable protons bound to respectively the dimer and the monomer, pH' is the integration variable and $\mathrm{pH_{ref}}$ is an arbitrary reference pH. It is important to note that this integration provides the shape of the $\Delta G^{\circ}(\mathrm{pH})$ profile, rather than its absolute vertical position.

The contributions of each site i to the changes of the $\Delta\Delta G^{\circ}(\mathrm{pH})$ profile is given by Equation 2, where n_i is the occupancy of site i. This decomposition allows us to determine the specific residues that play a crucial role for the pH sensitivity of the dimerization process.

$$\Delta\Delta G_i^{\circ}(\mathrm{pH}) = \ln(10)\, RT \int_{\mathrm{pH_{ref}}}^{\mathrm{pH}} \left(\langle n_i\rangle^{\mathrm{D}} - 2\langle n_i\rangle^{\mathrm{M}}\right)\, d\mathrm{pH}' \tag{2}$$

Because CpHMD simulations provide protonation data only at a few discrete pH points, a reliable numerical integration scheme is required. We therefore employ a recently-developed method[S12] based on an analytical expression of the integral, where the integrand is repre-

sented by a Hermite cubic spline.[S30] This method uses the fact that the slope of the total protonation curve is given by[S28,S29]

$$\frac{\mathrm{d}\langle n\rangle}{\mathrm{dpH}} = -\ln(10)\,\mathrm{var}(n)\ , \tag{3}$$

and the slope of a site-specific protonation curve is given by[S12]

$$\frac{\mathrm{d}\langle n_i\rangle}{\mathrm{dpH}} = -\ln(10)\,\mathrm{cov}(n_i, n)\ , \tag{4}$$

where $\mathrm{var}(n)$ is the variance of the total number of bound protons at the considered pH value and $\mathrm{cov}(n_i, n)$ is the covariance between the ocupancy of i and the total number of bound protons. Having the sampled average protonations and their corresponding slopes, we can then derive an interpolating Hermite cubic spline[S30] and easily compute its integral as an analytical expression (since each curve segment is a cubic polynomial, the integral is a sum of quartic polynomials. This is described in detail in the rest of this section.

Given a set of n points $(x_1, y_1), (x_2, y_2), \ldots, (x_n, y_n)$, with $x_1 < x_2 < \cdots < x_n$, and the associated slopes $y'_1, y'_2, \ldots, y'_n$, we can define an interpolating Hermite cubic spline $f(x)$ so that, for $x \in [x_i, x_{i+1}]$, we have (see section 5.3 of ref[S30])

$$f(x) = h_i(x) = \sum_{k=0}^{3} a_{ik}(x - x_i)^k\ ,$$

with

$$\begin{aligned}
a_{i0} &= y_i \\
a_{i1} &= y'_i \\
a_{i2} &= \frac{3(y_{i+1} - y_i)/(x_{i+1} - x_i) - 2y'_i - y'_{i+1}}{(x_{i+1} - x_i)} \\
a_{i3} &= \frac{y'_i + y'_{i+1} - 2(y_{i+1} - y_i)/(x_{i+1} - x_i)}{(x_{i+1} - x_i)^2}\ .
\end{aligned}$$

This ensures that $h_{i-1}(x_i) = h_i(x_i) = y_i$ and $h'_{i-1}(x_i) = h'_i(x_i) = y'_i$ for $2 \leq i \leq n-1$, meaning that $f(x)$ and $f'(x)$ are continuous in $[x_1, x_n]$.

An analytic expression for the spline integral can be obtained by summing the contributions from each interval. Thus, the integral of $f(x)$ from the initial point x_1 to a point $x \in [x_k, x_{k+1}]$ can be written as

$$\int_{x_1}^{x} f(t)\,\mathrm{d}t = \sum_{i=1}^{k-1} g_i(x_{i+1}) + g_k(x) \ ,$$

where

$$g_i(x) = \int_{x_i}^{x} h_i(t)\,\mathrm{d}t = \sum_{k=0}^{3} \frac{a_{ik}}{k+1}\,(x - x_i)^{k+1}$$

is the interval-specific integral of h_i between x_i and an arbitrary value $x \in [x_i, x_{i+1}]$. The integral of $f(x)$ is then a piecewise quartic polynomial.

1.5 Fraction of Monomers

To compute the *absolute* monomer fraction at each pH would require the absolute dimerization free energy (rather than a relative value) and the total PsbS concentration, which depends on thylakoid-level factors (expression, turnover, etc.). However, as shown below, it is possible to derive the relative increase of the monomer fraction.

The concentrations of the monomeric and dimeric forms of PsbS can be expressed as

$$[\mathrm{M}] = f\,[\mathrm{P}] \qquad \text{and} \qquad [\mathrm{D}] = (1-f)[\mathrm{P}]/2 \ , \tag{5}$$

where [P] is the total concentration of PsbS protomers (i.e., chains) and f is the fraction of protomers that are present as monomers, such that the dimerization equilibrium constant can be written as

$$K = \frac{[\mathrm{D}]}{[\mathrm{M}]^2} = \frac{(1-f)}{2f^2[\mathrm{P}]} \ . \tag{6}$$

For the present purposes, any convenient units can be chosen for the protein concentrations,

such as mol/liter, mol/(thylakoid surface area), mol/chloroplast, etc.[S31] If the total amount of PsbS is kept the same (i.e., constant [P]) and the system experiences a pH change $\mathrm{pH}_1 \rightarrow \mathrm{pH}_2$, the ratio of the two corresponding pH-dependent dimerization constants is

$$r = \frac{K(\mathrm{pH}_2)}{K(\mathrm{pH}_1)} = \frac{(1-f_2)f_1^2}{(1-f_1)f_2^2}\,, \tag{7}$$

where f_1 and f_2 are the monomer fractions corresponding to respectively pH_1 and pH_2, which gives

$$f_2 = \frac{f_1\sqrt{f_1^2 + 4r(1-f_1)} - f_1^2}{2r(1-f_1)}\,. \tag{8}$$

Taking into account that the change $\mathrm{pH}_1 \rightarrow \mathrm{pH}_2$ is thermodynamically equivalent to the two-step sequence $\mathrm{pH}_1 \rightarrow \mathrm{pH}_{\mathrm{ref}} \rightarrow \mathrm{pH}_2$, the ratio r can be computed in terms of the relative dimerization free energies computed through Equation 1, namely

$$r = \mathrm{e}^{-[\Delta\Delta G(\mathrm{pH}_2) - \Delta\Delta G(\mathrm{pH}_1)]/RT}\,. \tag{9}$$

Therefore, given a monomer fraction at some pH value pH_1, we can compute the monomer fraction at any other pH value pH_2 using the last two equations and the previously computed pH-dependent relative dimerization free energies.

1.6 Other Analysis

Standard analyses were carried out using the GROMACS package[S13] and in-house scripts on the final 400 ns of each trajectory after equilibration. The equilibration period was determined by monitoring the time evolution of multiple properties, including the root-mean-square deviation (Figure S1), secondary structure content (Figure S2), and protonation states (Figure S3).

The solvent accessible surface area (SASA) of the protein was calculated with the GROMACS `sasa` tool, using a rolling probe with a radius of 0.14 nm to simulate the surface

traced by a water molecule. To evaluate the interaction surface between the two chains of the dimer, the contact surface area was determined using $\frac{(S_\mathrm{A}+S_\mathrm{B}-S_\mathrm{dimer})}{2}$, where S_A and S_B represent the solvent accessible surface areas of chain A and chain B when considered individually, and S_dimer is the surface area of the full dimer.

Protein tilt angles were calculated using the GROMACS `gangle` tool. The global tilt of PsbS was defined by an orientation vector connecting the centers of mass of two atom groups: one comprising four Cα atoms from the stroma side and the other four Cα atoms from the lumen side of each transmembrane helix (TM1–TM4) . The angle between this vector and the membrane normal (z-axis) was then computed. The tilt of helix H2 was determined in an analogous manner, using a vector defined by the first and last Cα atoms of the helix, and measuring its angle relative to the z-axis.

Titration curves were generated by calculating the average protonation state of each titratable residue at each pH level, considering only the equilibrated portion of the simulation. To extract the pK_a values and Hill coefficients (h) for each site, the Hill equation was fitted to the data[S32] using a nonlinear least-squares fitting method implementend in `gnuplot`.[S33]

$$f(\mathrm{pH}) = \left[1 + 10^{h(\mathrm{pH}-\mathrm{p}K_\mathrm{a})}\right]^{-1} \tag{10}$$

The protonation correlations between all pair of sites was calculated using Pearson's correlation coefficient.[S34] The correlation between sites i and j is defined as a function of the variances and covariance of their binding states as seen in Equation 11.

$$\rho_{ij} = \frac{\mathrm{cov}(n_i, nj)}{\sqrt{\mathrm{var}(n_i)\mathrm{var}(n_j)}} \tag{11}$$

The tendency of two sites to have either identical or opposed binding states will lead to a positive or negative correlation, respectively, having a zero value if the occupancies of the two sites are completely unrelated.

The correlation time for each site i was estimated as the time at which the autocorrelation

function (ACF) of its proton occupancy (0 or 1) n_i dropped below 0.1. The global ACF was computed as the average over the replicate-specific ACFs, each computed as shown in Equation 12.

$$C(t) = \frac{\langle (n_i(0) - \langle n_i \rangle) \, (n_i(t) - \langle n_i \rangle) \rangle}{\mathrm{var}(n_i)} \tag{12}$$

Unless otherwise stated, all statistical uncertainties were calculated as the standard error of the mean over the replicates. To estimate statistical uncertainties for the titration curve fits, which yield the computed pK_a values, a bootstrap resampling protocol was applied.[S35] All plots were generated using the `gnuplot` software,[S33] while molecular structures and visual representations were prepared with `PyMOL`.[S36]

2 Equilibration

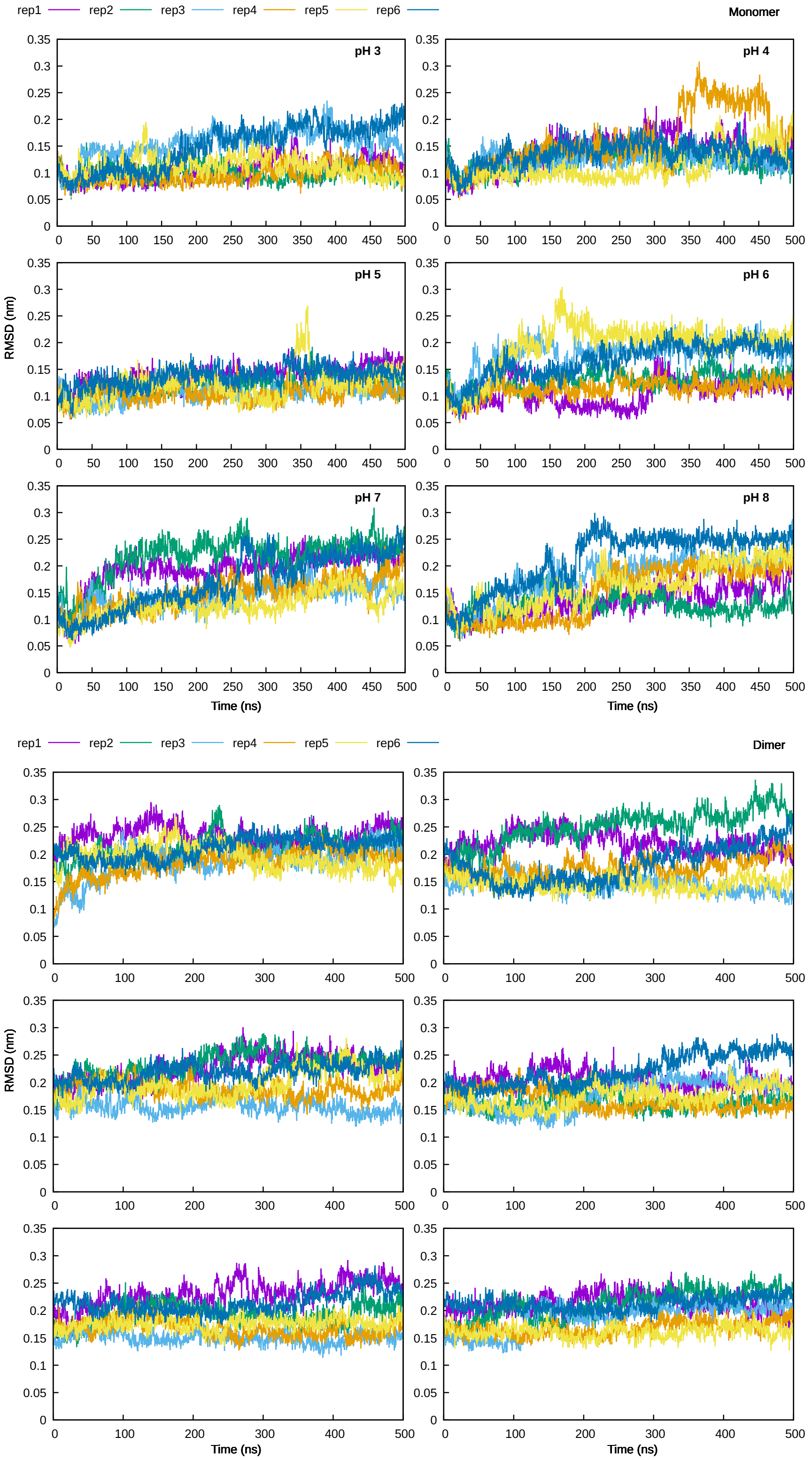

Figure S1: RMSD of the PsbS monomer and dimer as a function of time for different pH conditions (pH 3–8). Each plot shows six independent replicates. RMSD values were calculated relative to the crystal structure, using the Cα atoms of the transmembrane helices (TM1–TM4) for both the fitting and the calculation of the RMSD values.

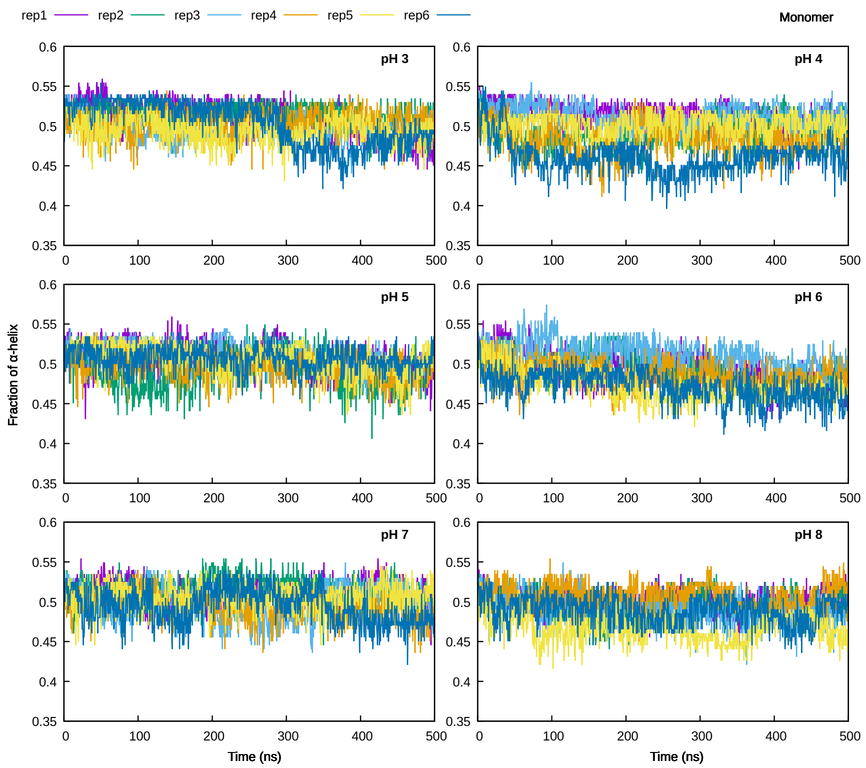

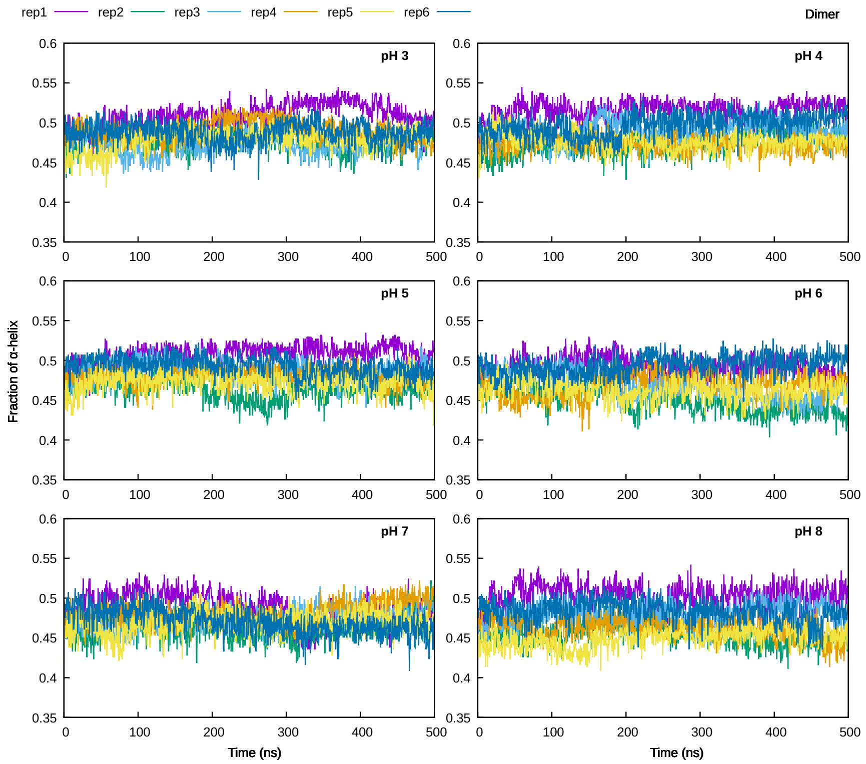

Figure S2: Time evolution of the fraction of residues in α-helical conformation for the PsbS monomer and dimer at different pH values (3–8). Each panel shows the results for six independent replicates (rep1–rep6).

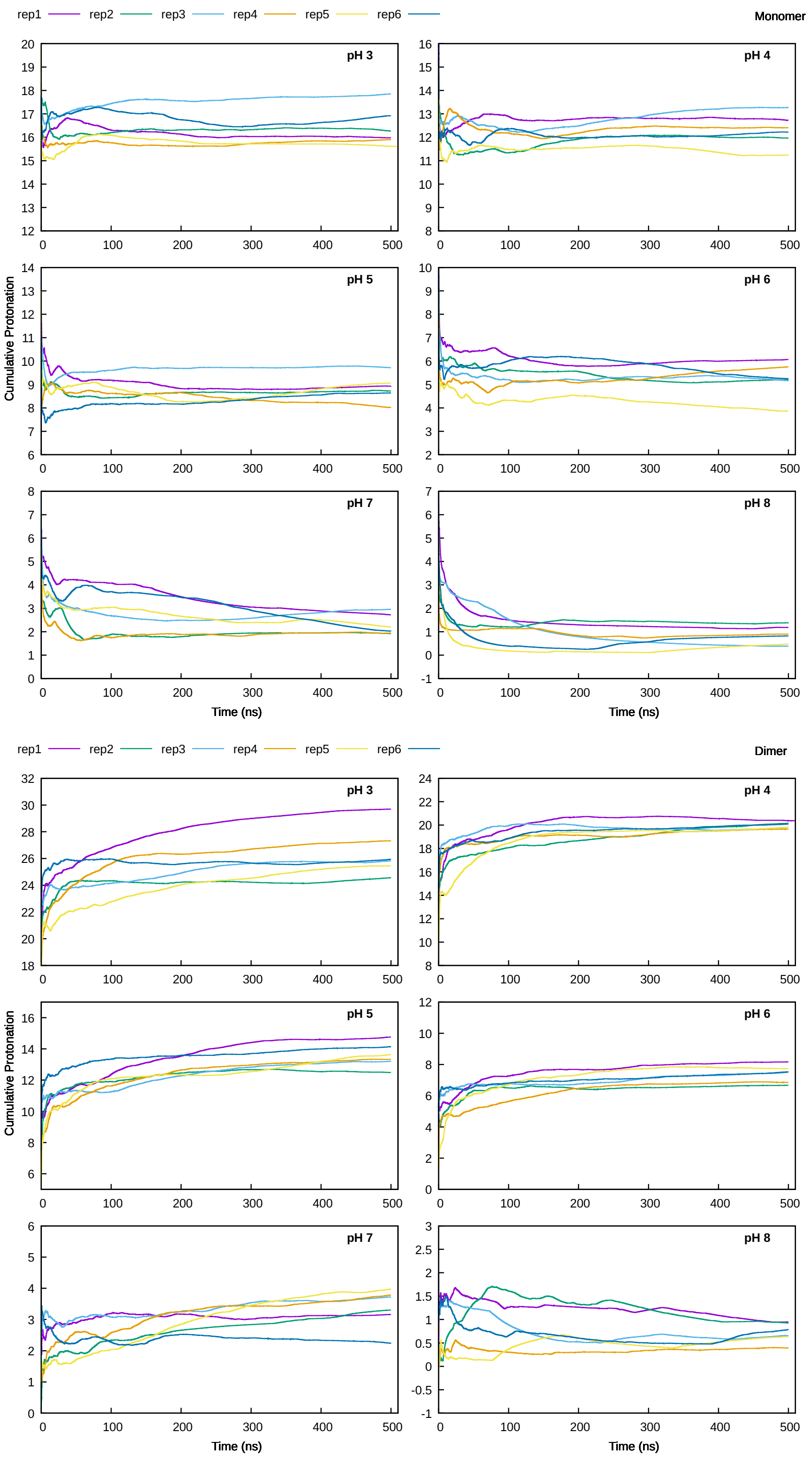

Figure S3: Cumulative number of protonated residues in the PsbS monomer and dimer as a function of simulation time under different pH conditions (pH 3–8). Each panel shows six independent replicates (rep1–rep6) represented by different colors.

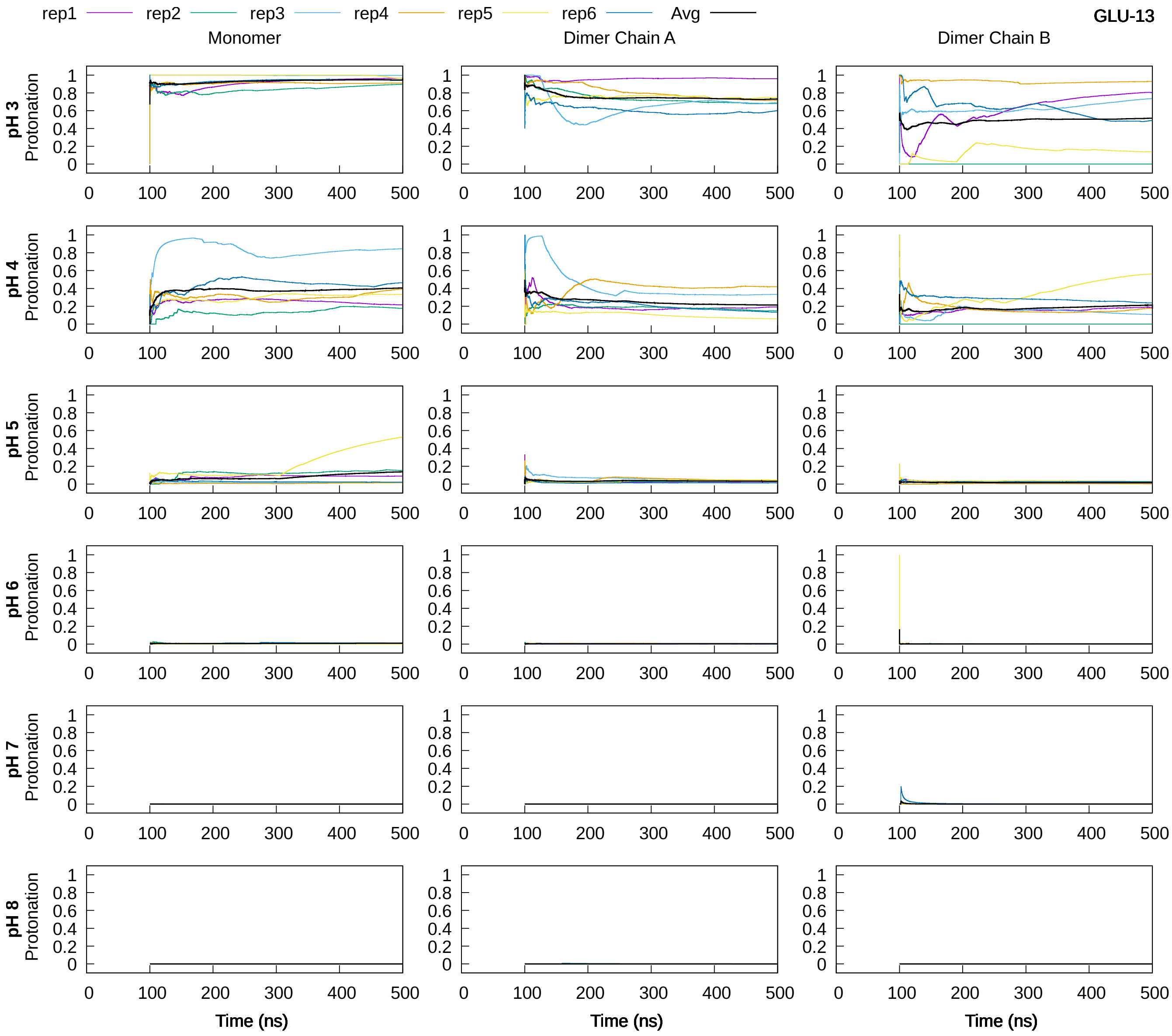

Figure S4: Simulation time series of the cumulative average of the protonation of PsbS sites. The black thick curve corresponds to the average over all replicates.

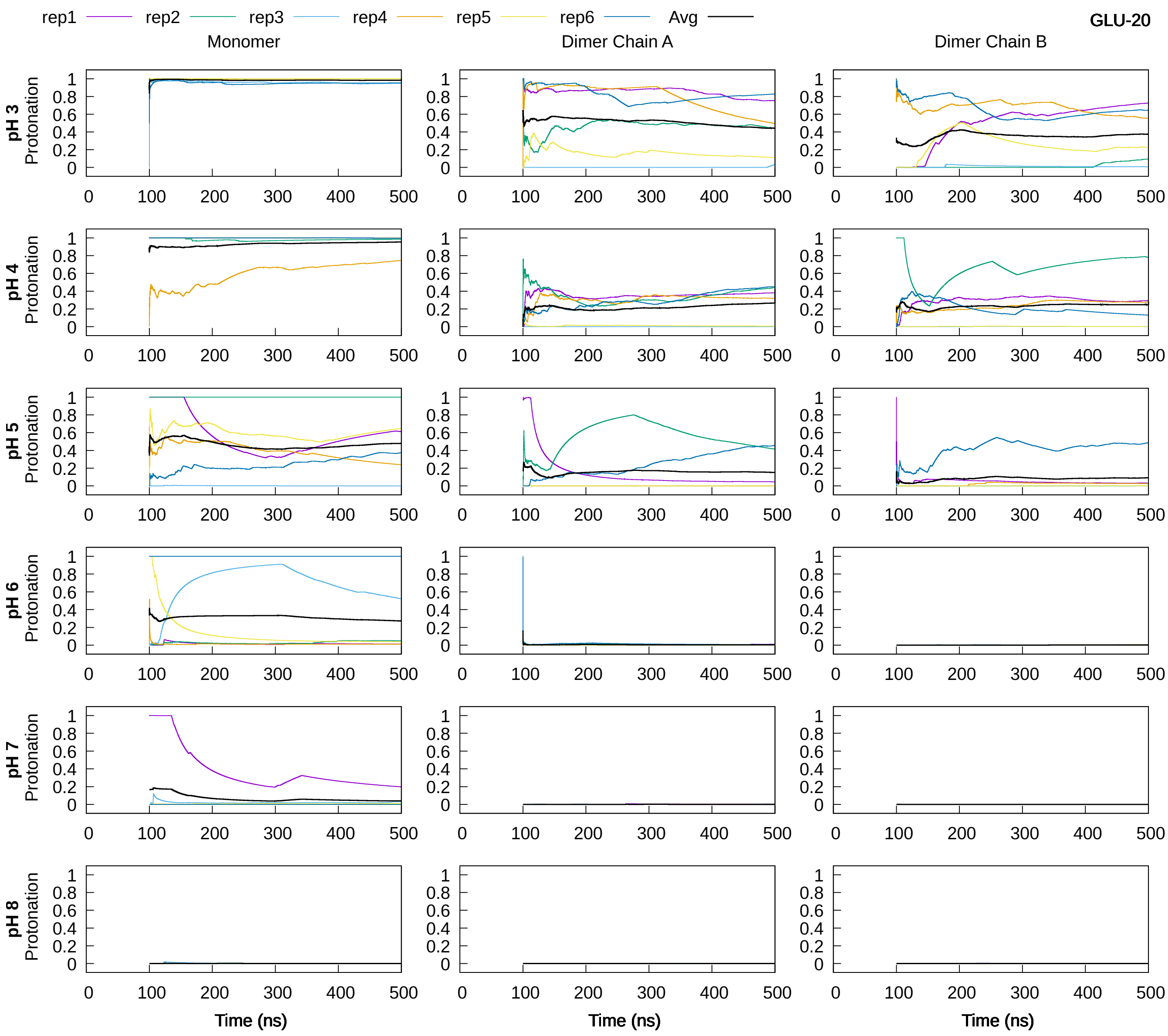

Figure S4: (continued, part 2)

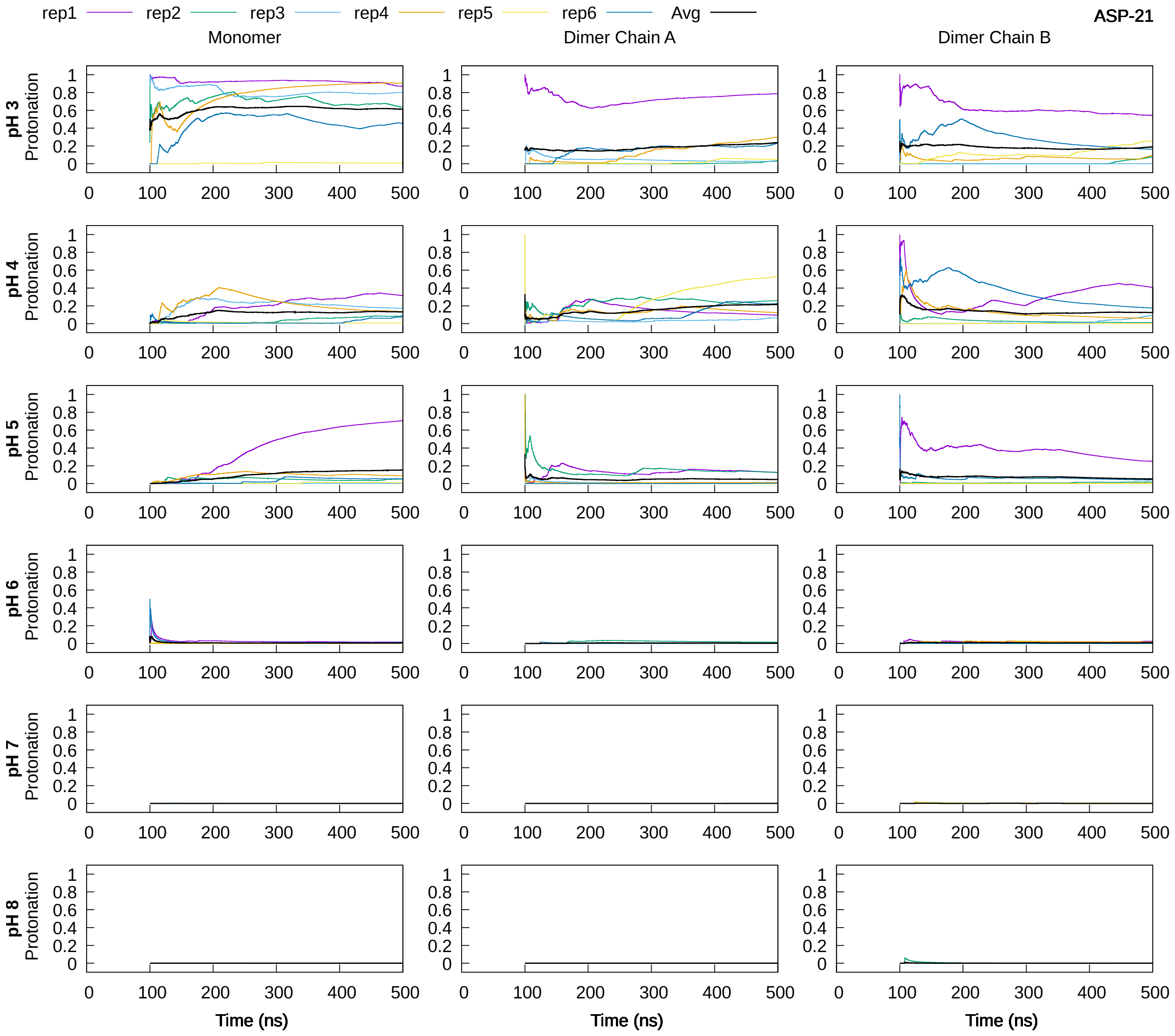

Figure S4: (continued, part 3)

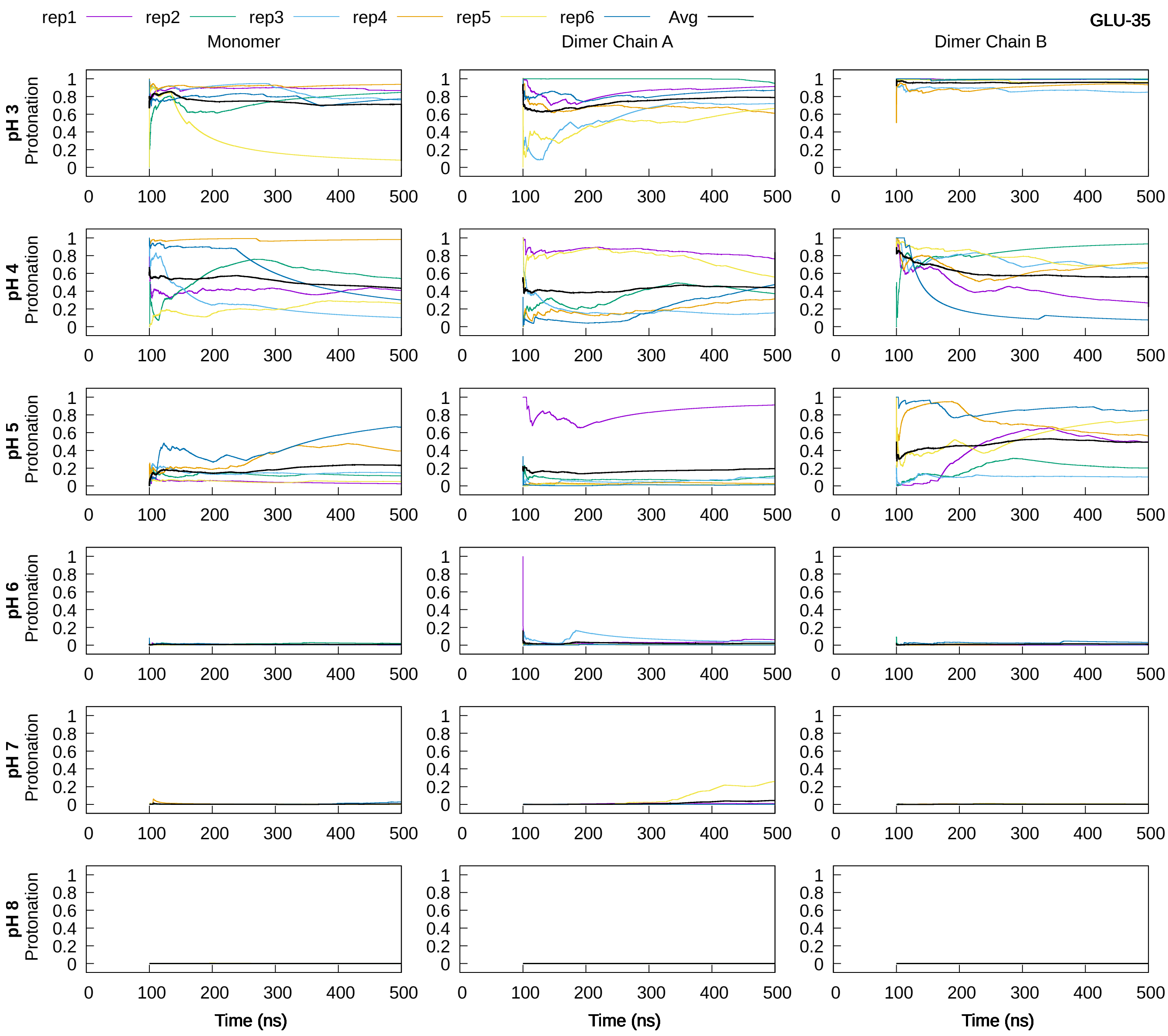

Figure S4: (continued, part 4)

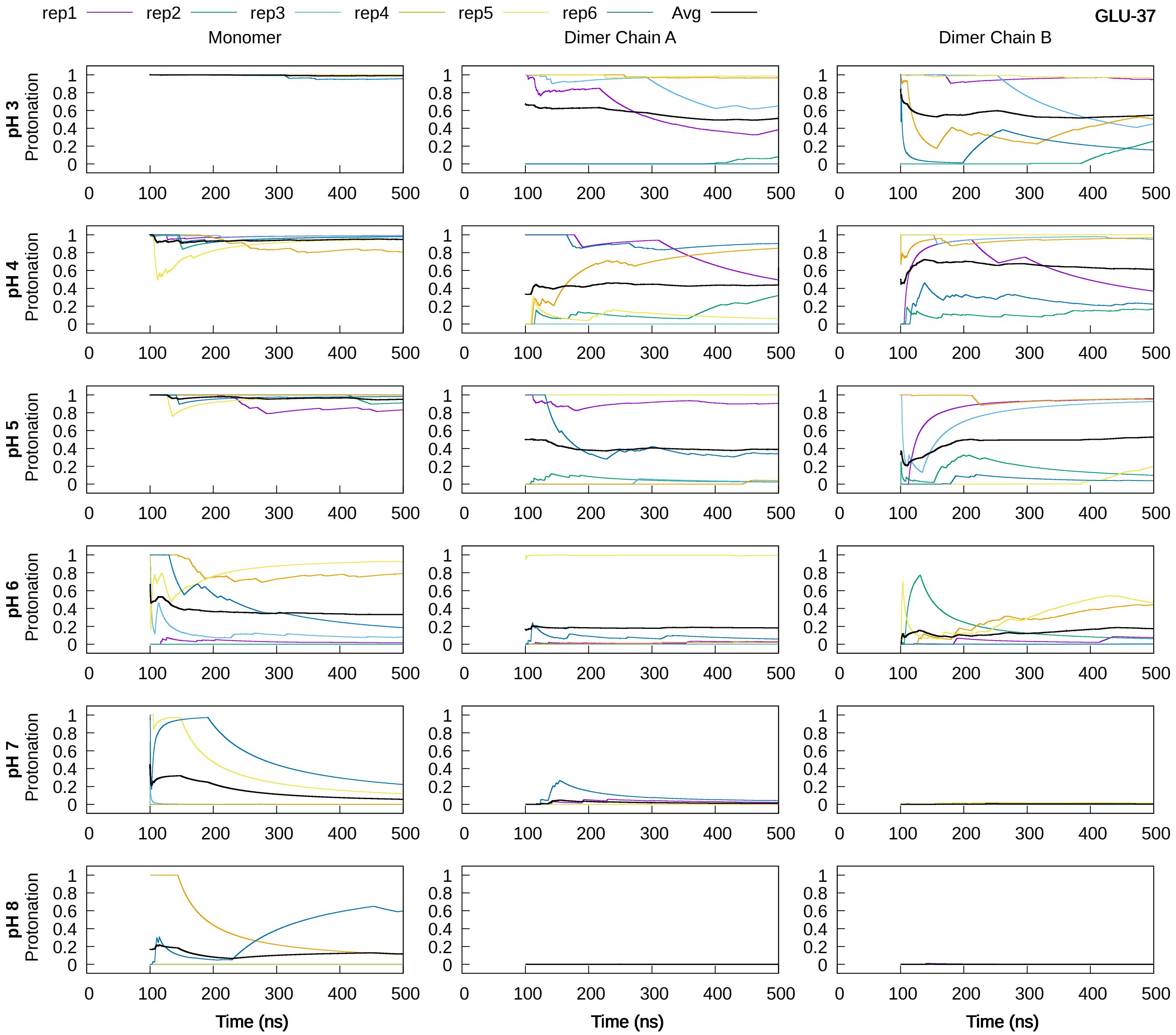

Figure S4: (continued, part 5)

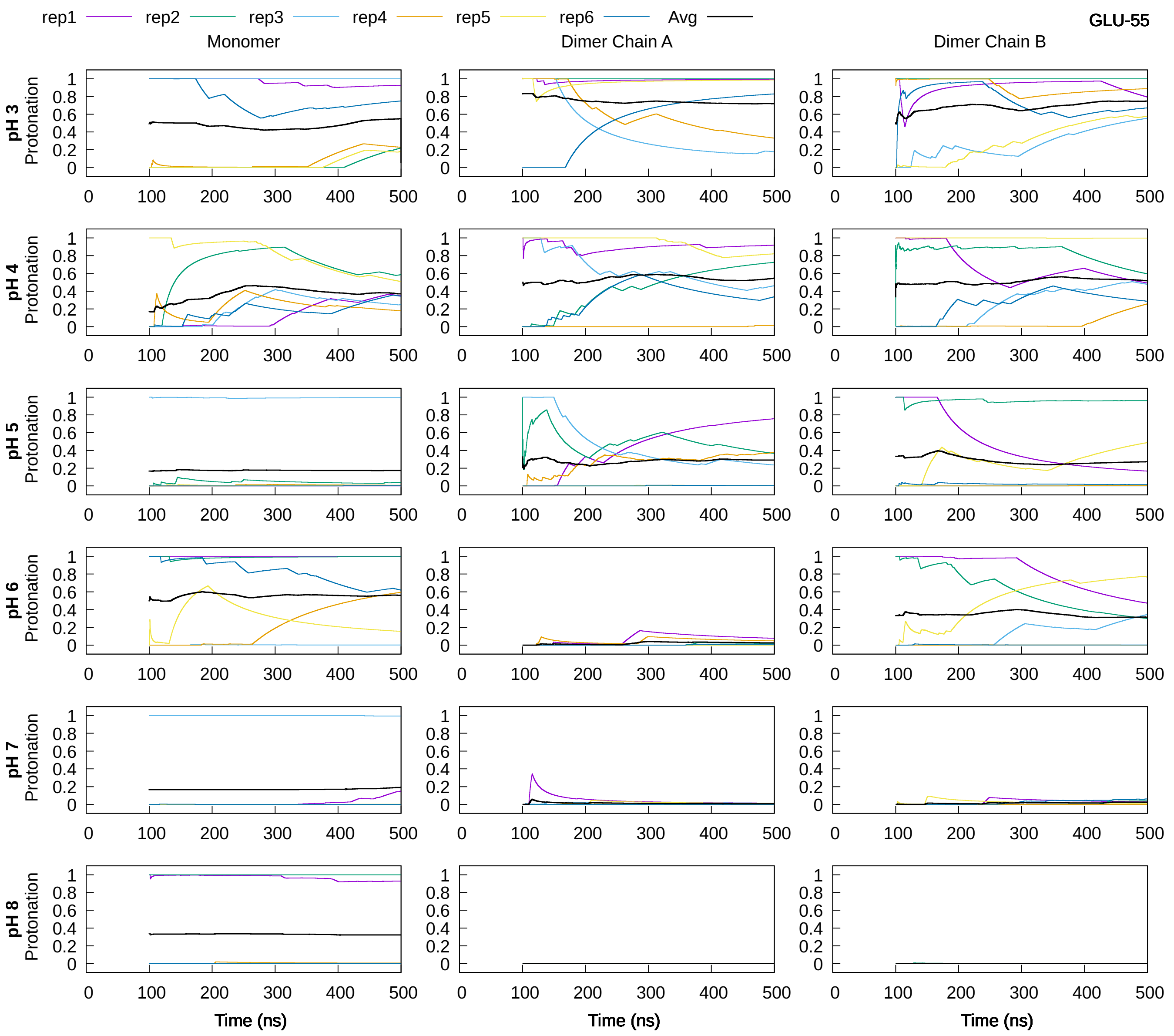

Figure S4: (continued, part 6)

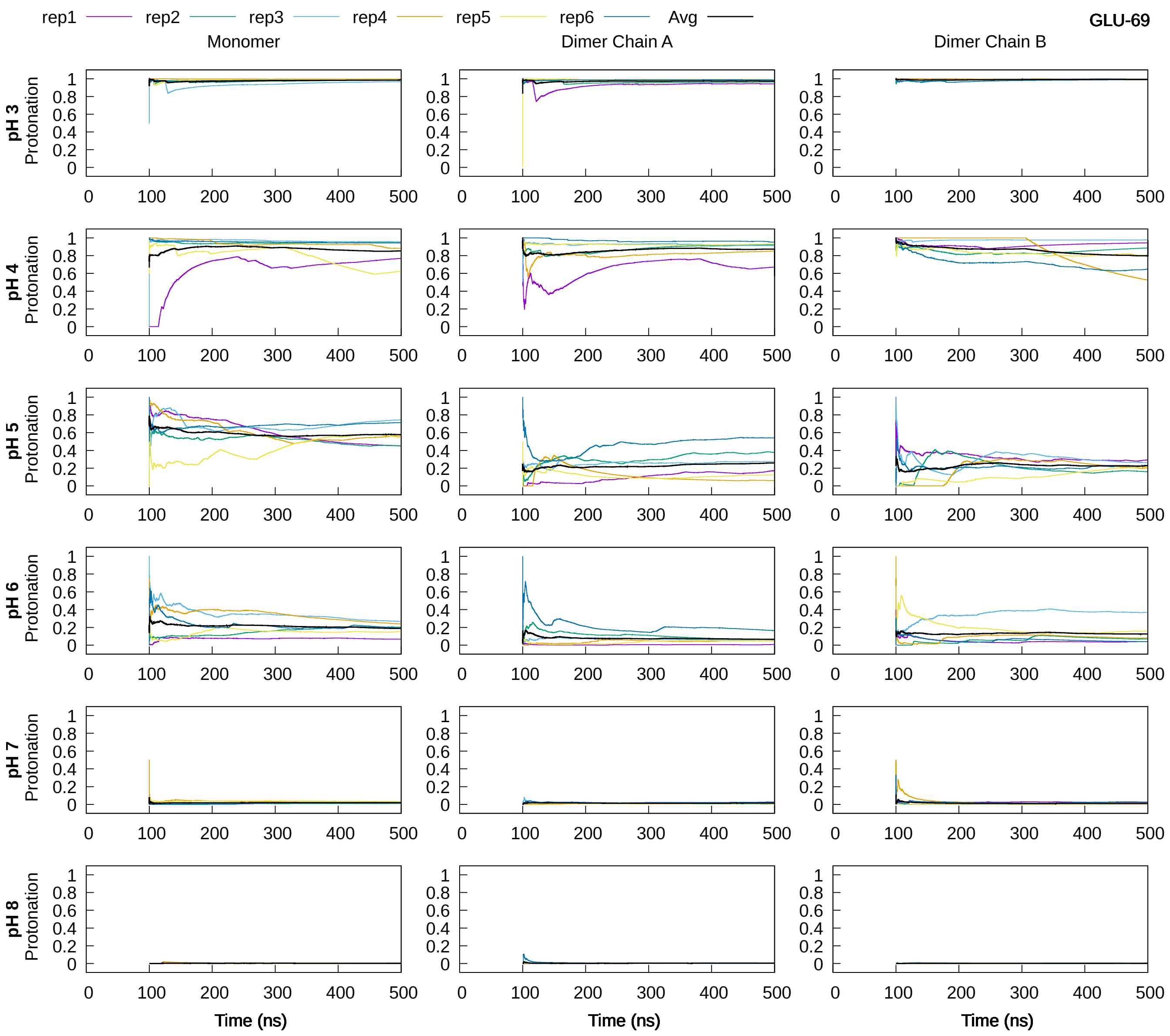

Figure S4: (continued, part 7)

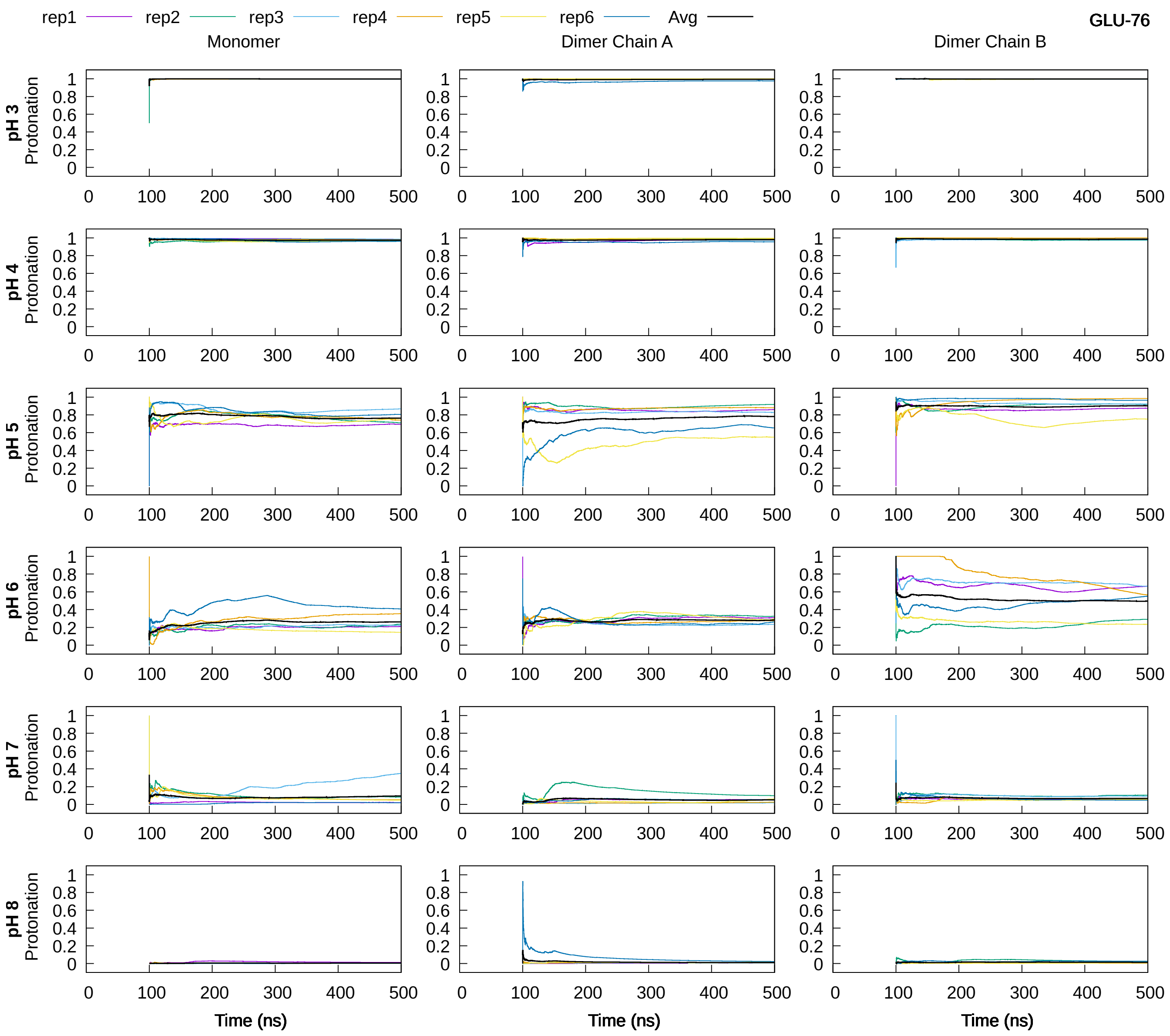

Figure S4: (continued, part 8)

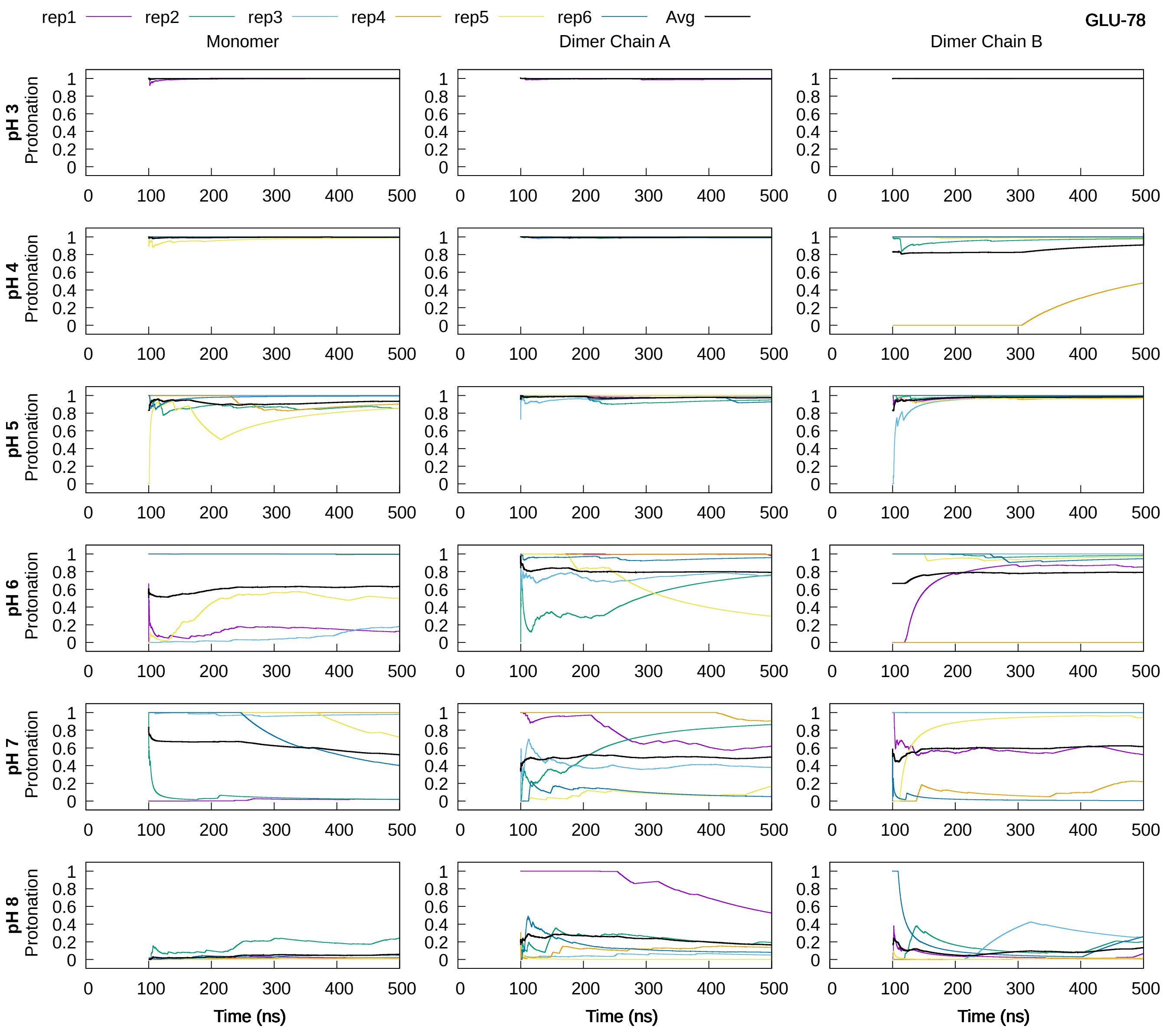

Figure S4: (continued, part 9)

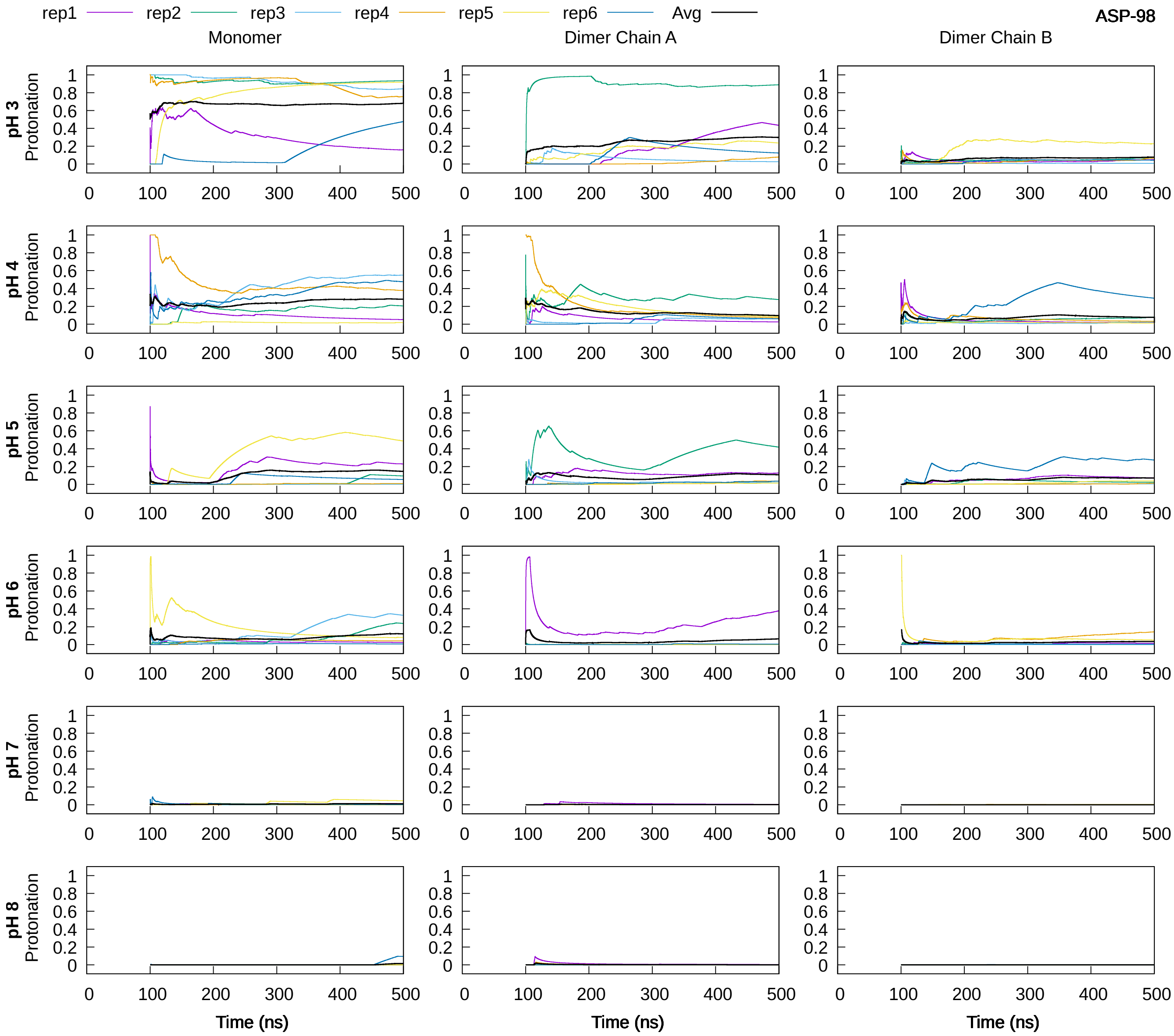

Figure S4: (continued, part 10)

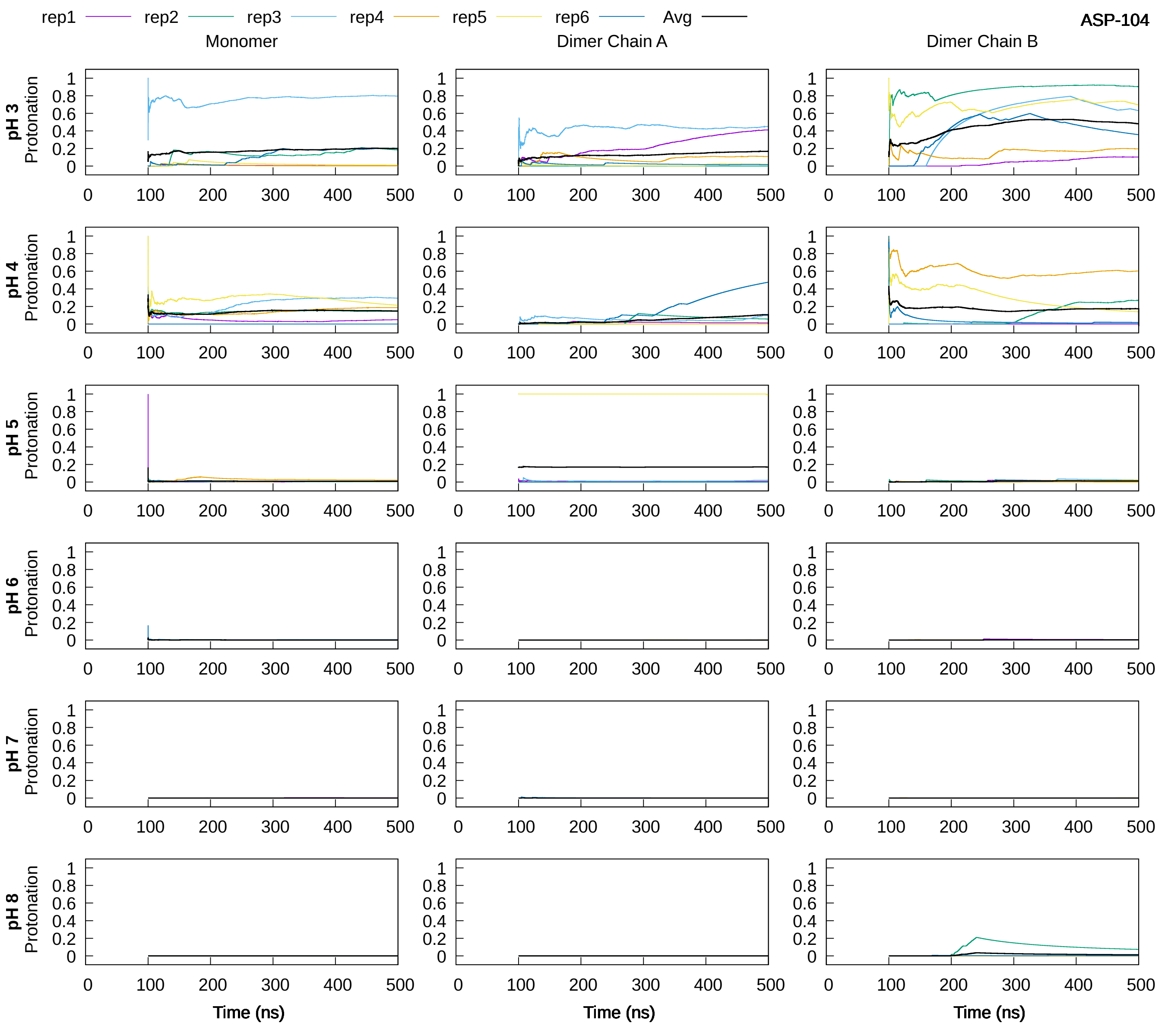

Figure S4: (continued, part 11)

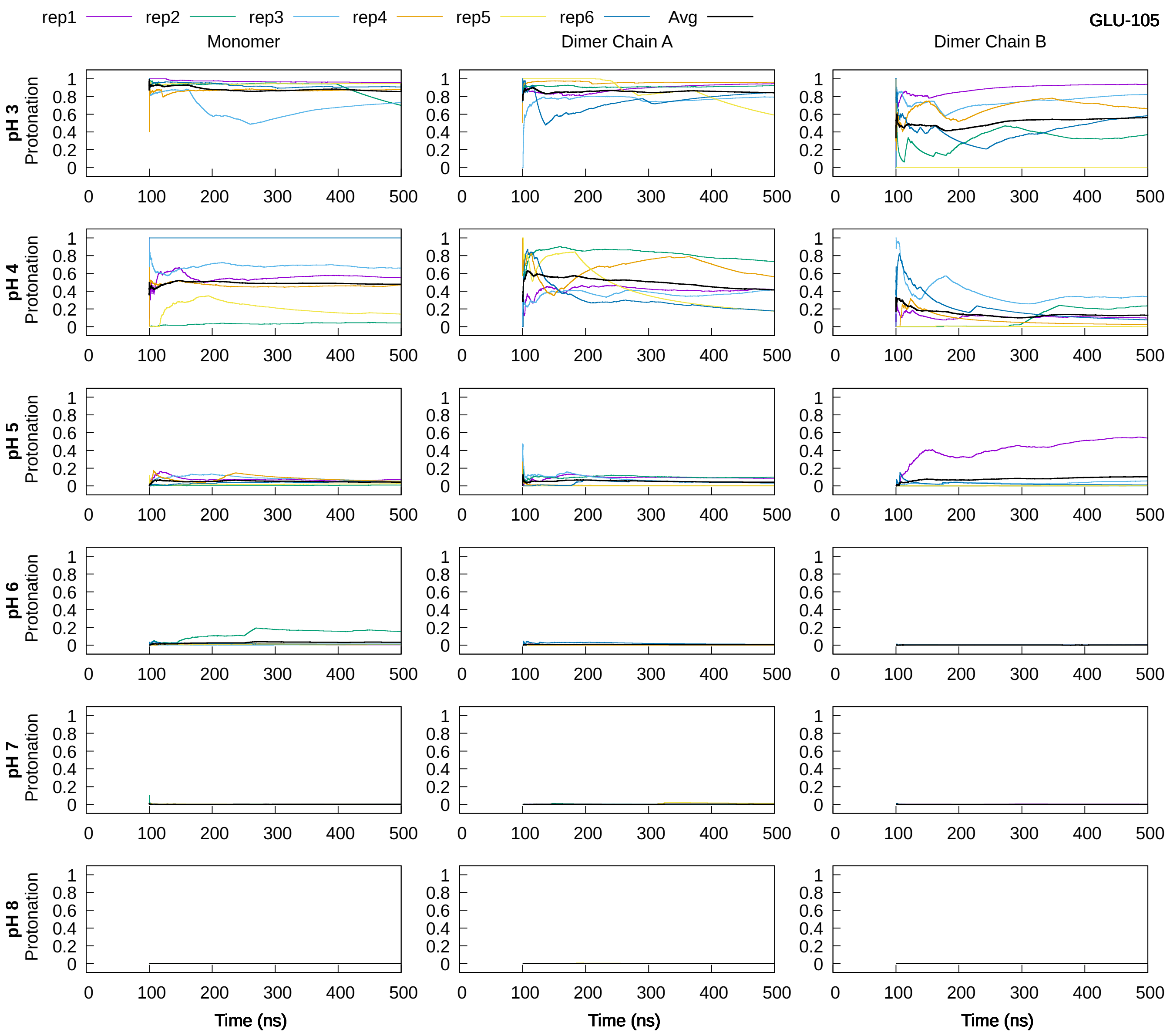

Figure S4: (continued, part 12)

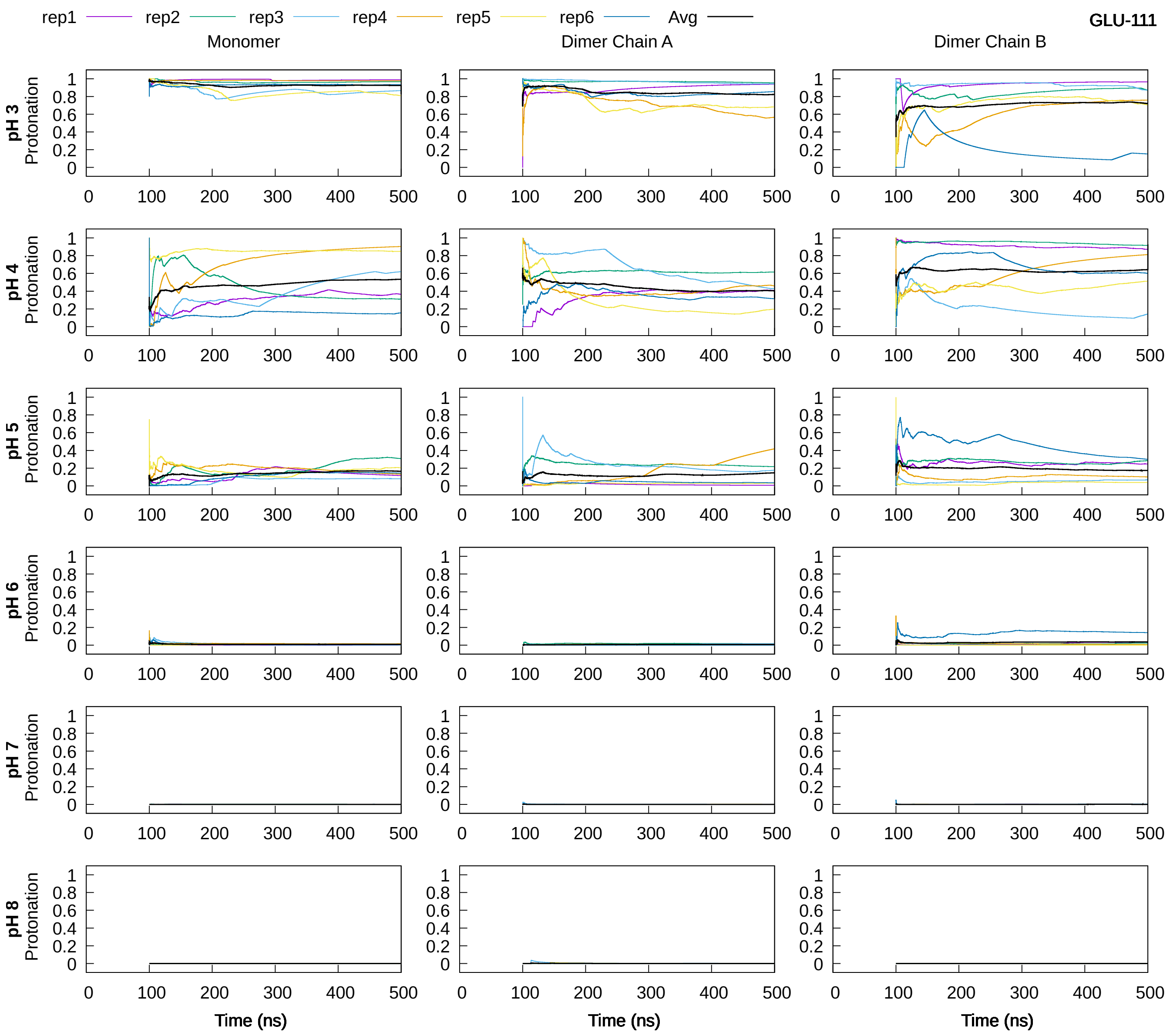

Figure S4: (continued, part 13)

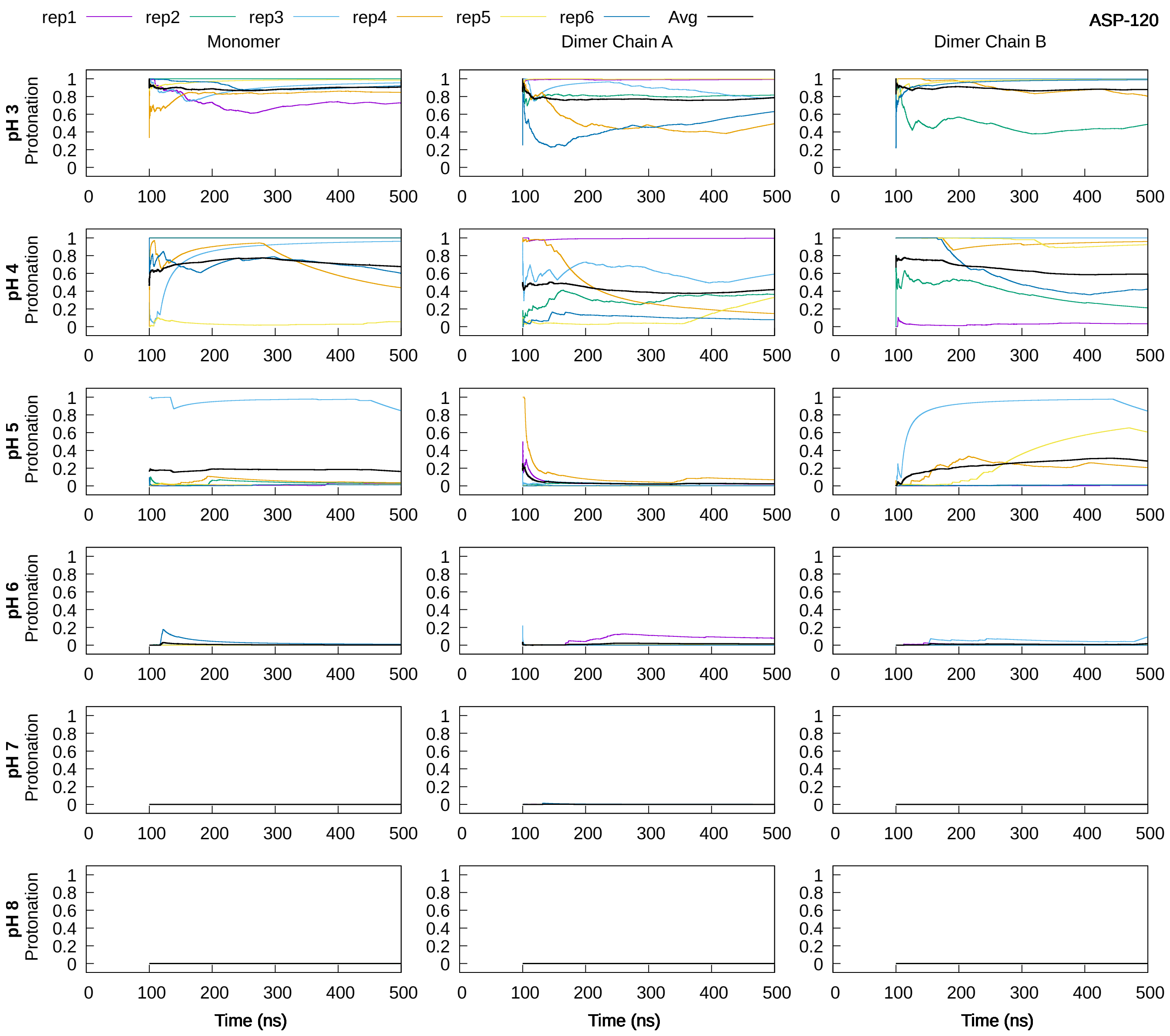

Figure S4: (continued, part 14)

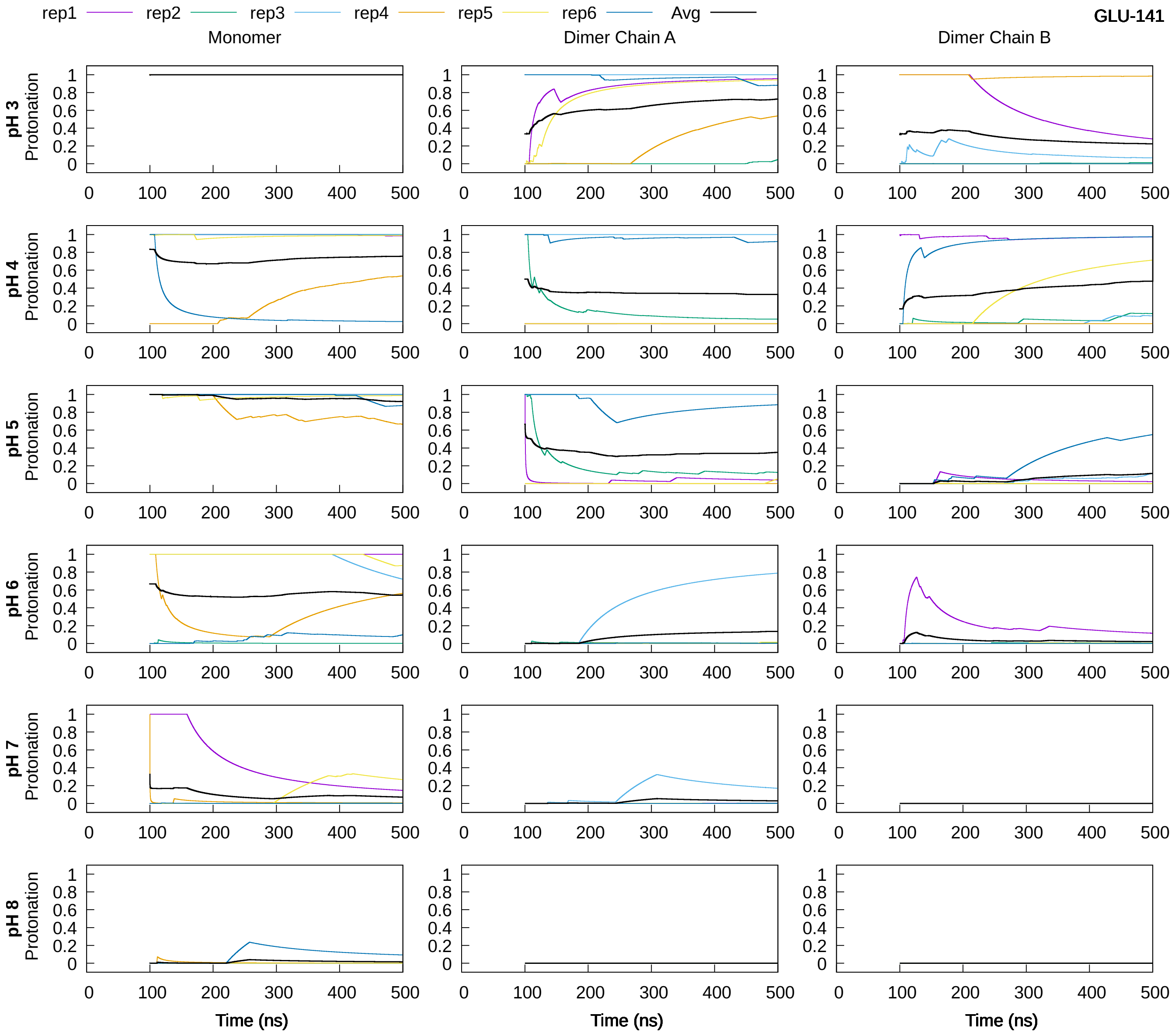

Figure S4: (continued, part 15)

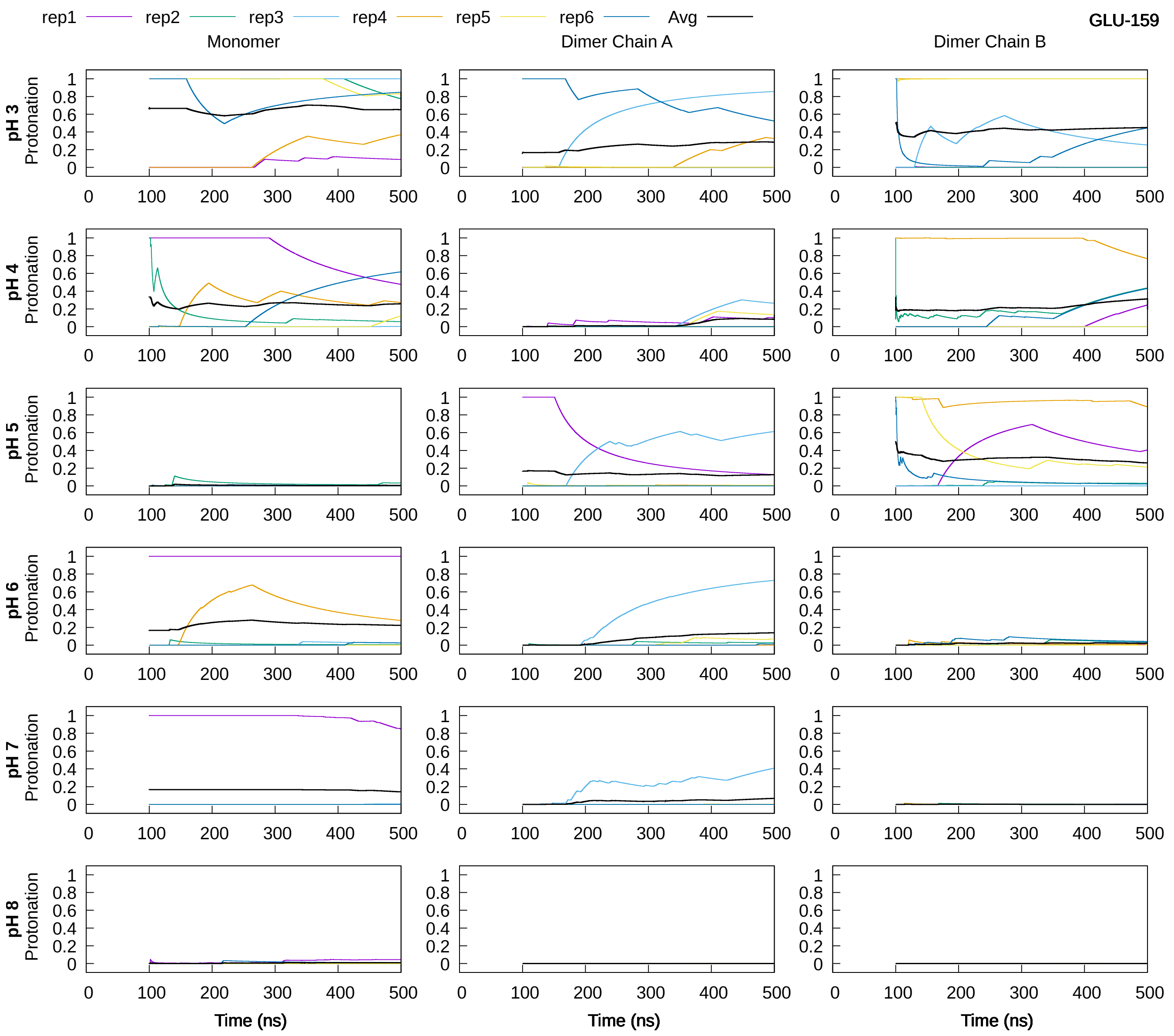

Figure S4: (continued, part 16)

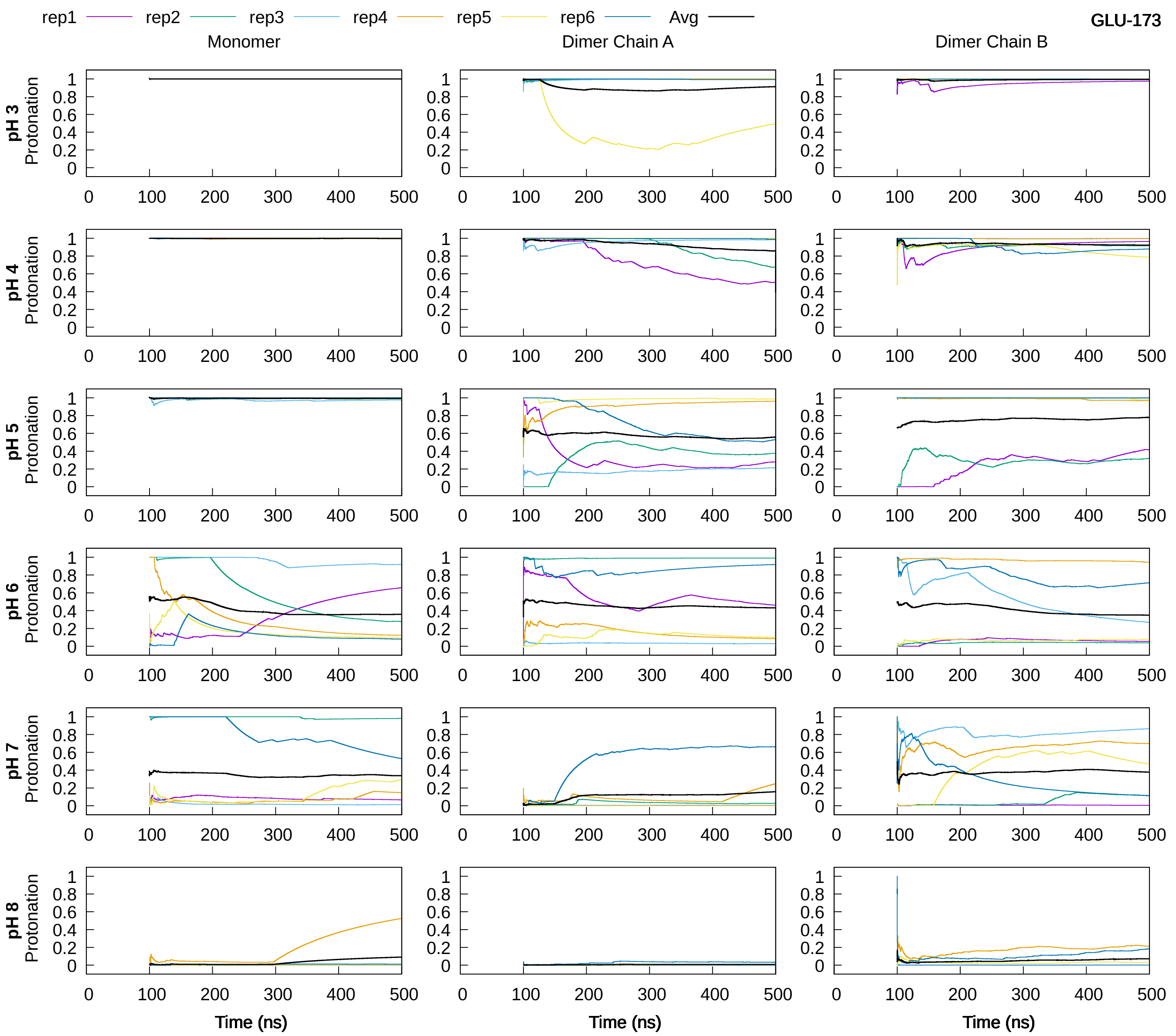

Figure S4: (continued, part 17)

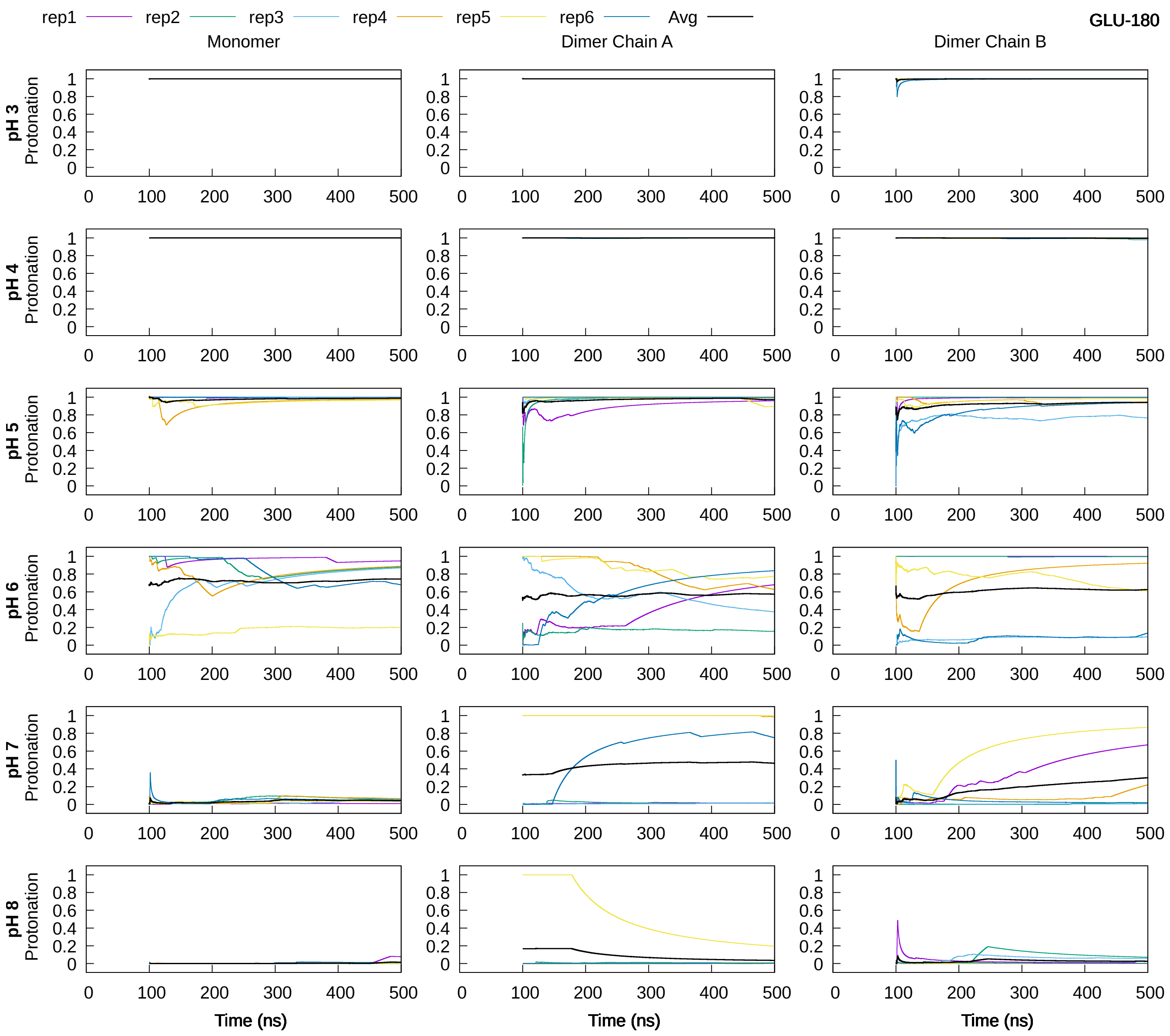

Figure S4: (continued, part 18)

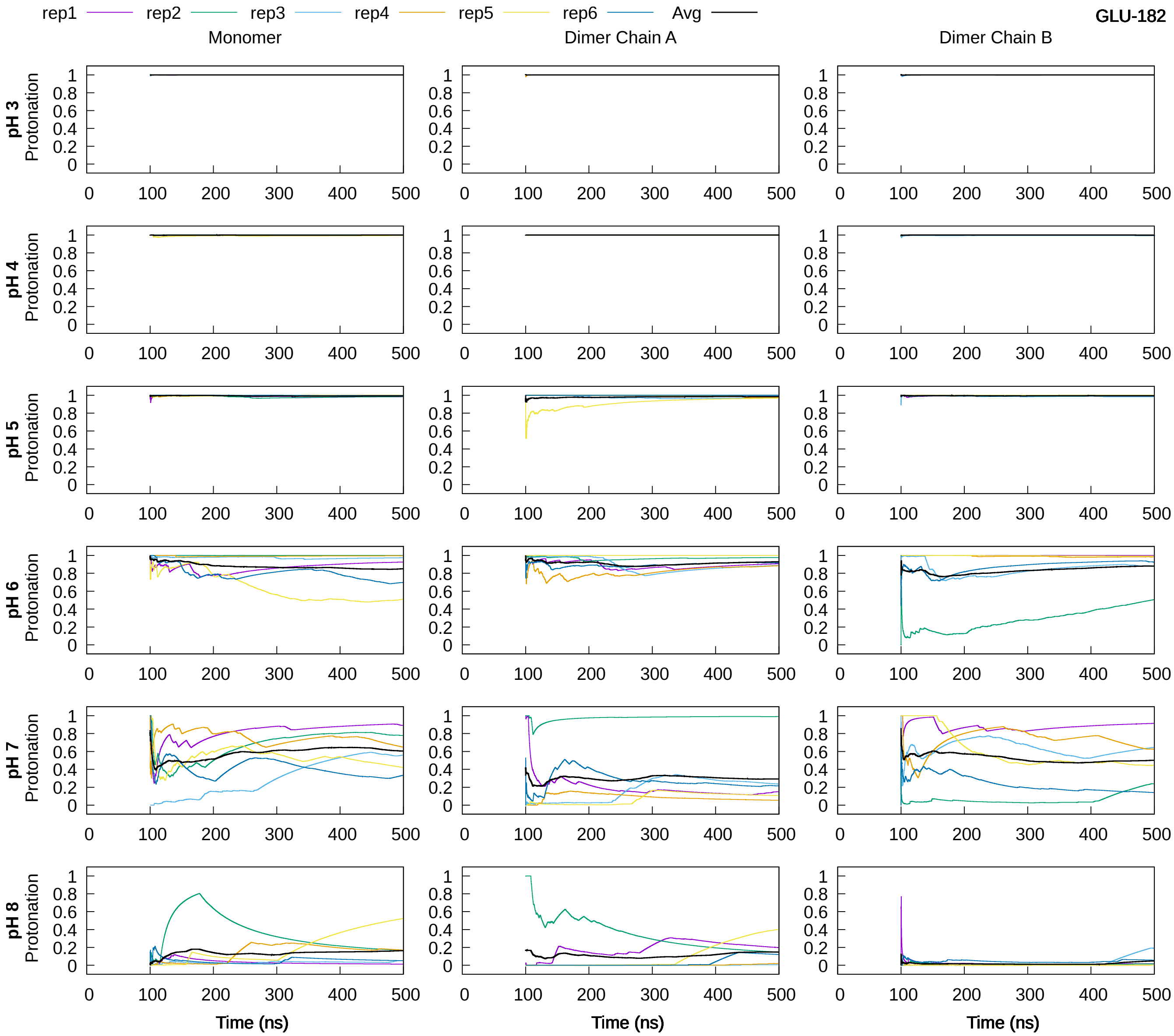

Figure S4: (continued, part 19)

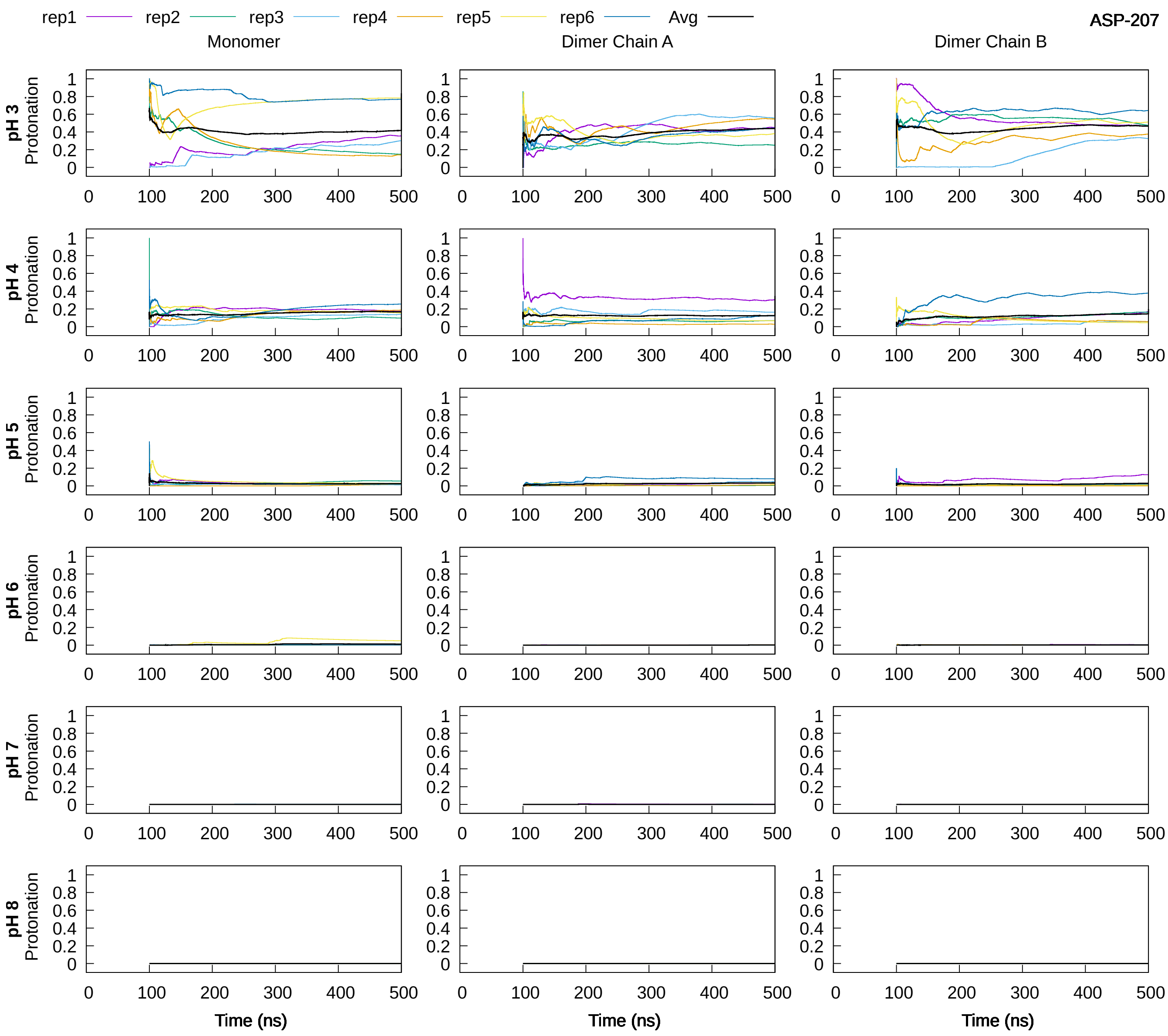

Figure S4: (continued, part 20)

3 Structural Analysis

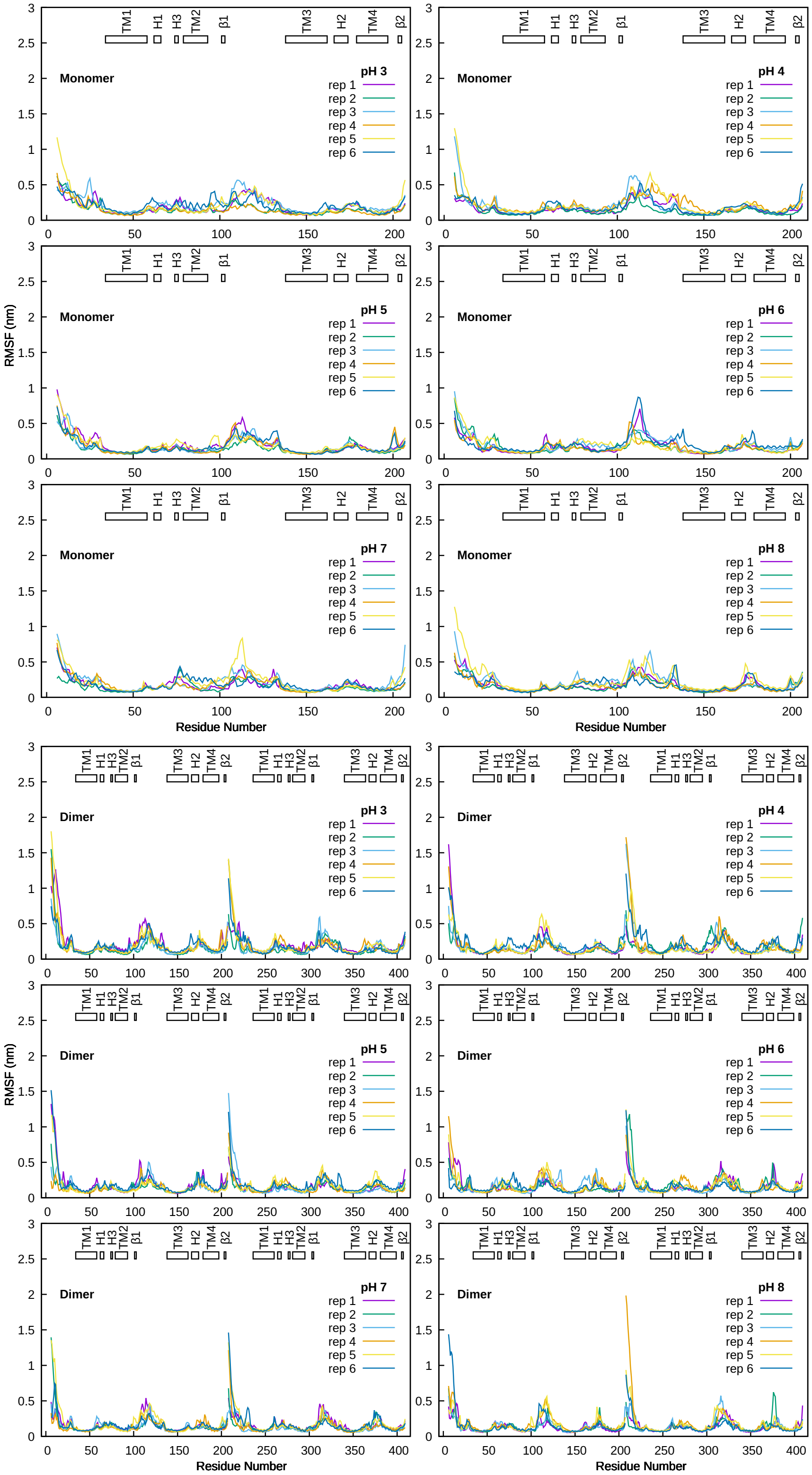

Figure S5: RMSF of the PsbS monomer and dimer at different pH values (pH 3–8). Each plot shows six independent replicates and the different domains labeled with rectangles.

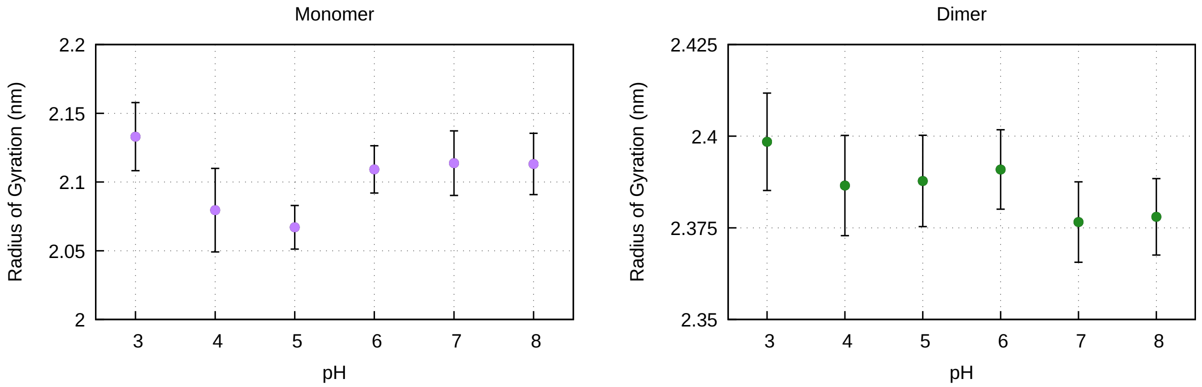

Figure S6: Radius of gyration (nm) of PsbS as a function of pH. Results are presented for the monomer and dimer. Error bars represent the standard error of the mean across six independent replicates.

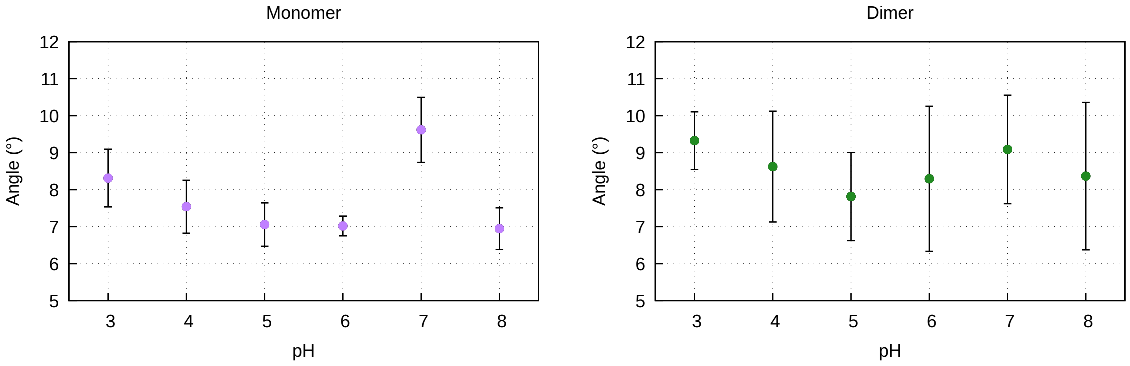

Figure S7: Tilt angle of PsbS relative to the membrane normal (z-axis) as a function of pH. Results are shown for the monomer and the dimer. Error bars represent the standard error of the mean across all replicates.

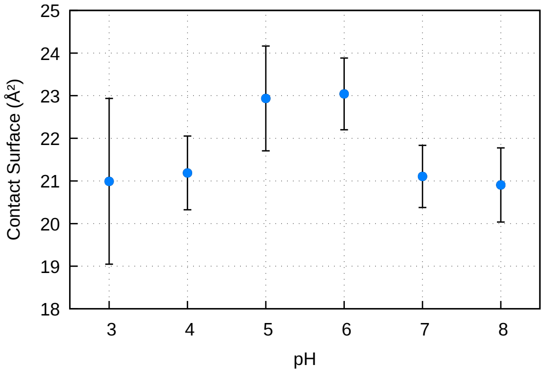

Figure S8: Contact surface of the PsbS dimer as a function of pH. The values represent the average contact surface area between the two monomers, with error bars indicating the standard error of the mean across the six replicates.

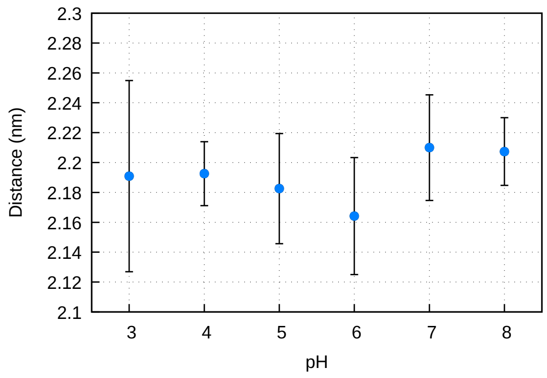

Figure S9: Interchain distance of the PsbS dimer, measured as the distance between the centers of mass of the two protein chains, as a function of pH. Error bars represent the standard error of the mean across all replicates.

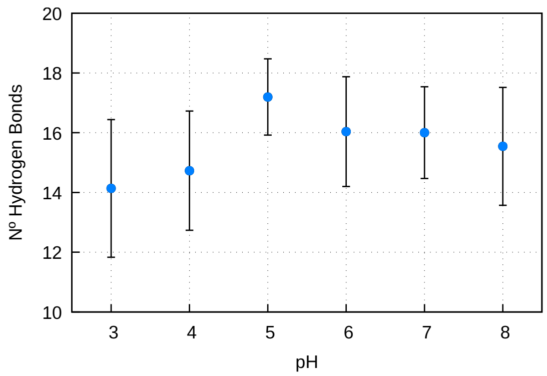

Figure S10: Average number of interchain hydrogen bonds in PsbS dimer as a function of pH. The error bars represent the standard error of the mean across six independent replicates.

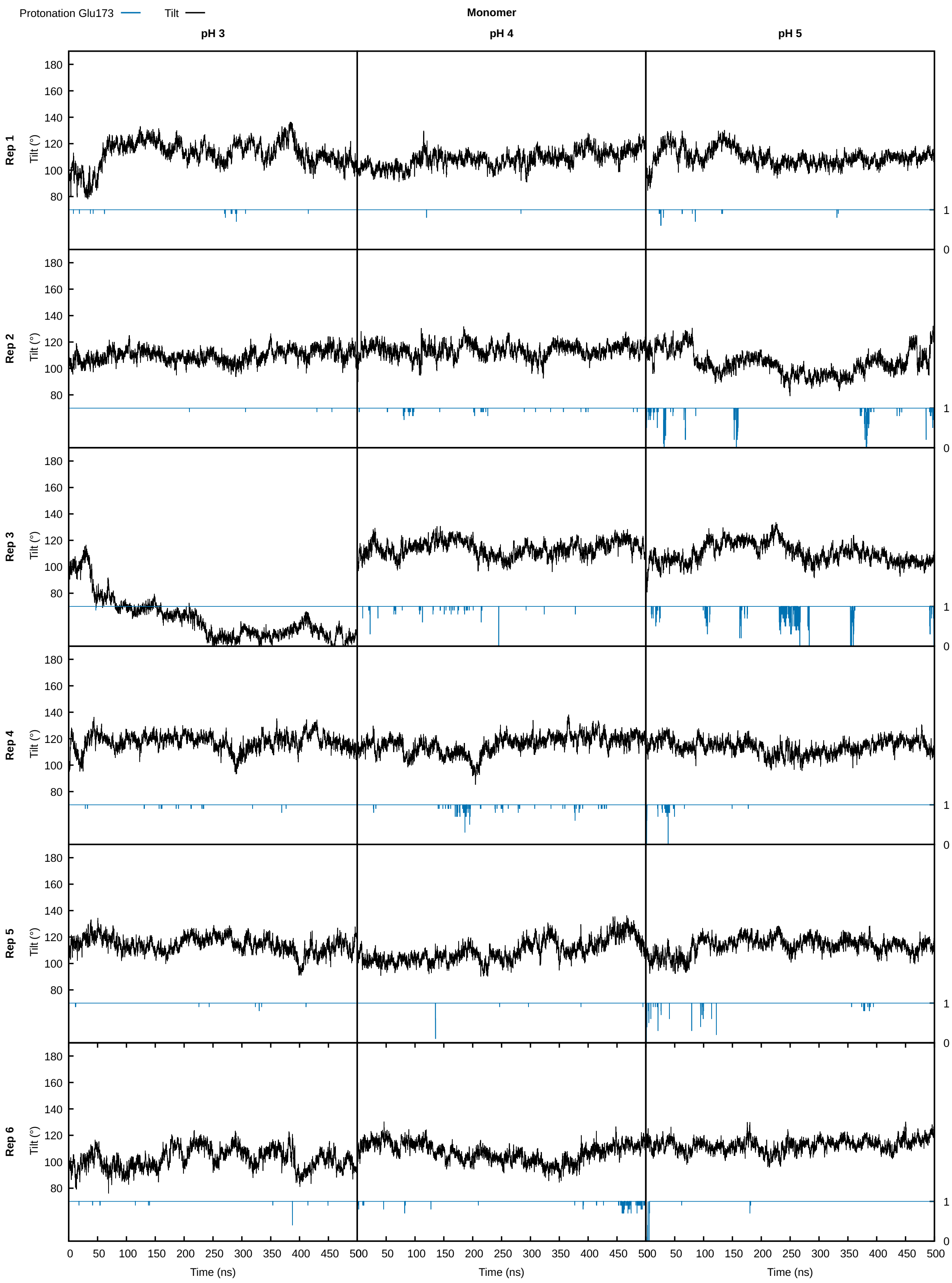

Figure S11: Block averages (0.1 ns windows) of tilt angle of helix H2 (black) relative to the membrane normal and protonation state of Glu173 (blue) are plotted across different pH conditions for the monomer.

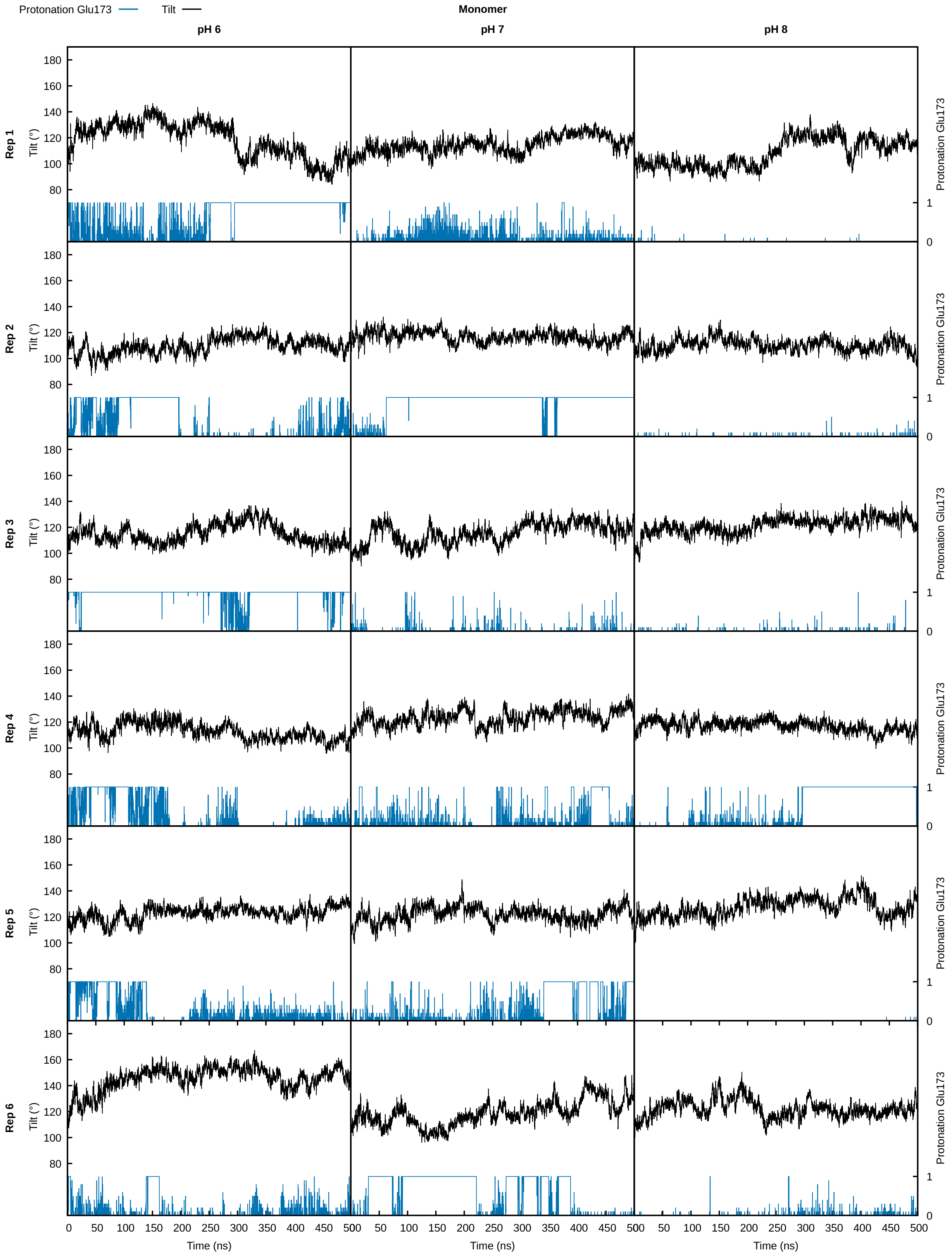

Figure S11: (continued)

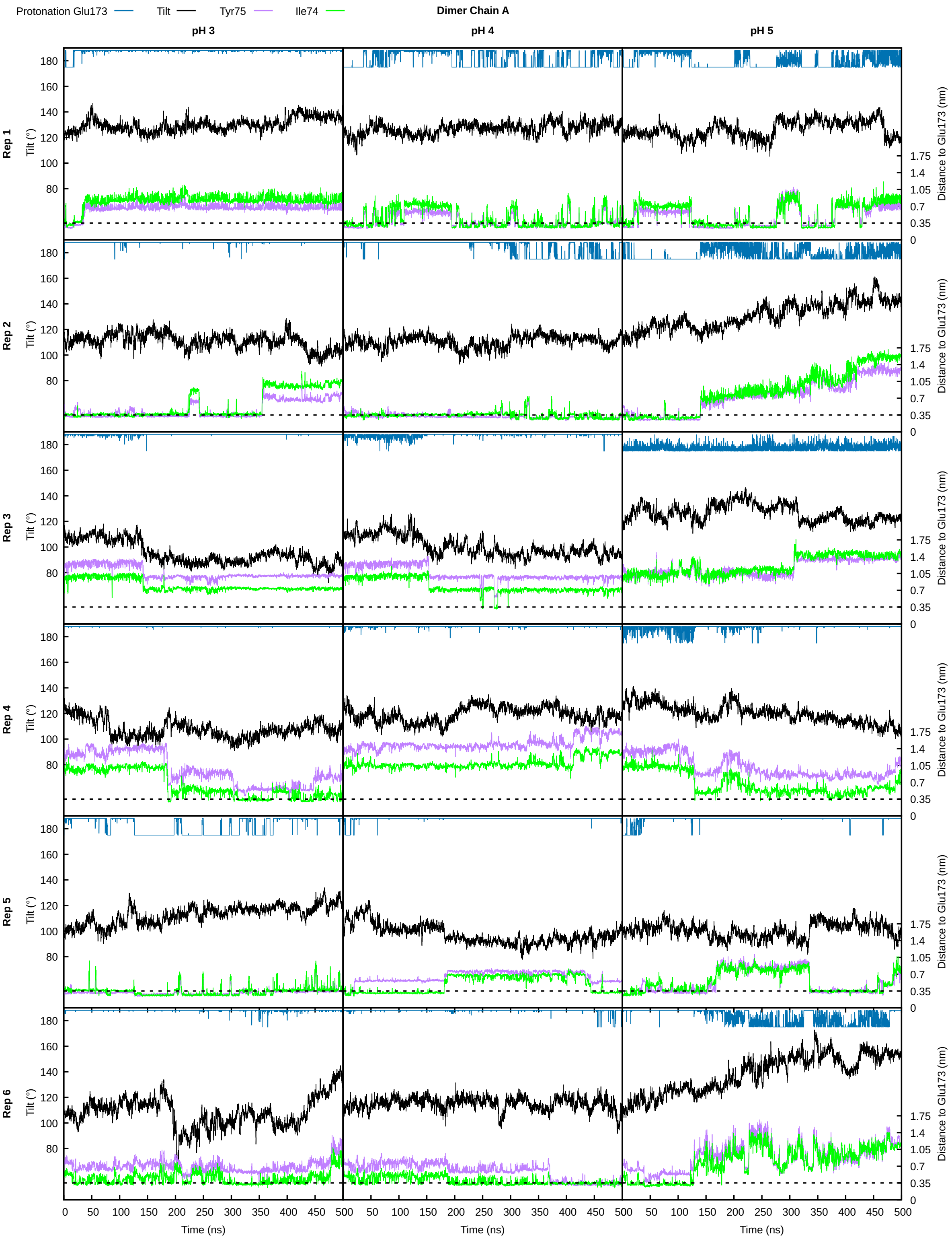

Figure S12: Block averages (0.1 ns windows) of tilt angle of helix H2 (black) relative to the membrane normal, protonation state of Glu173 (blue), and the distances between Glu173 (H2) and the backbone amide groups of Ile74 (green) and Tyr75 (purple) from helix H3 of the opposite chain, plotted across different pH conditions for both chains of the dimer.

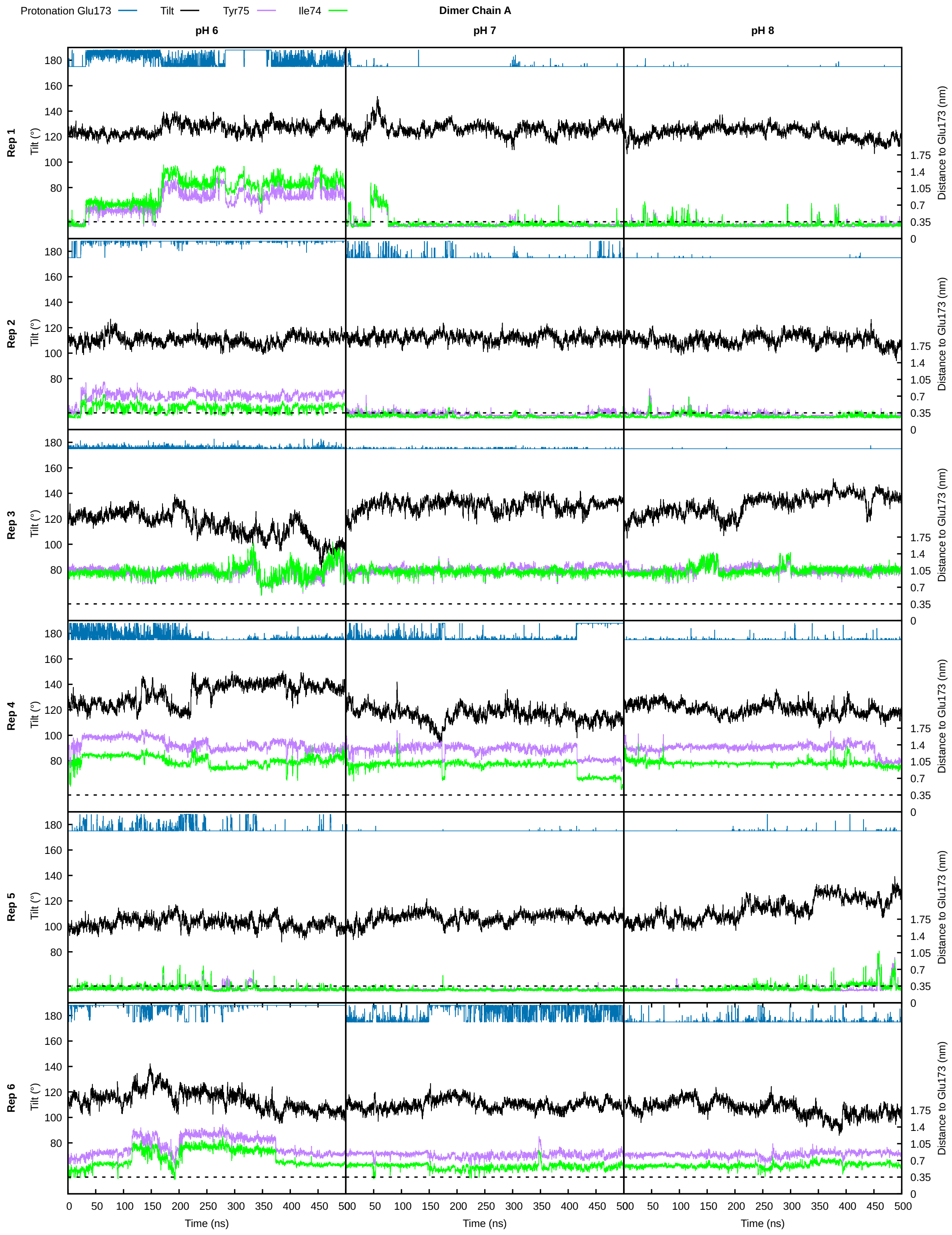

Figure S12: (continued)

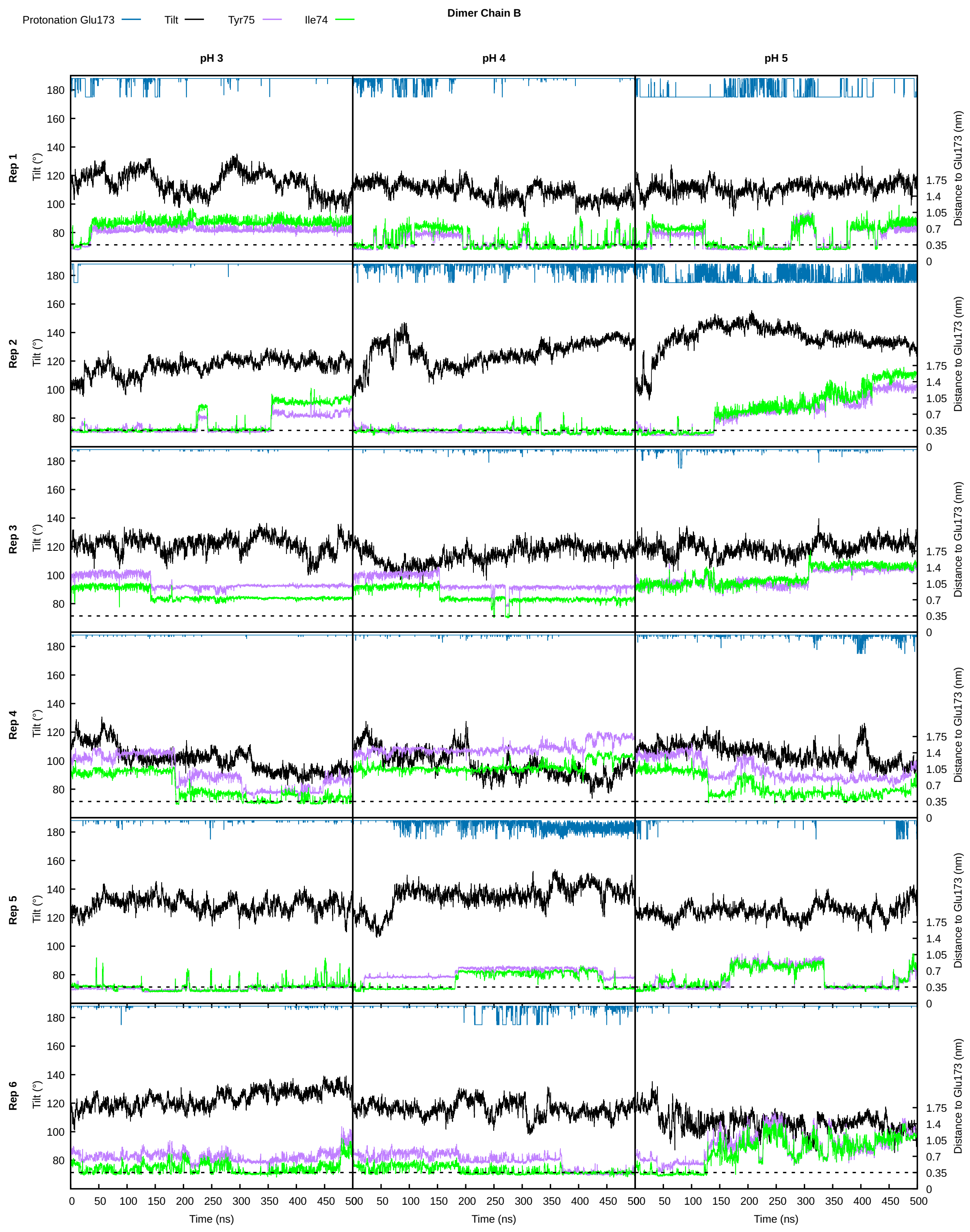

Figure S12: (continued)

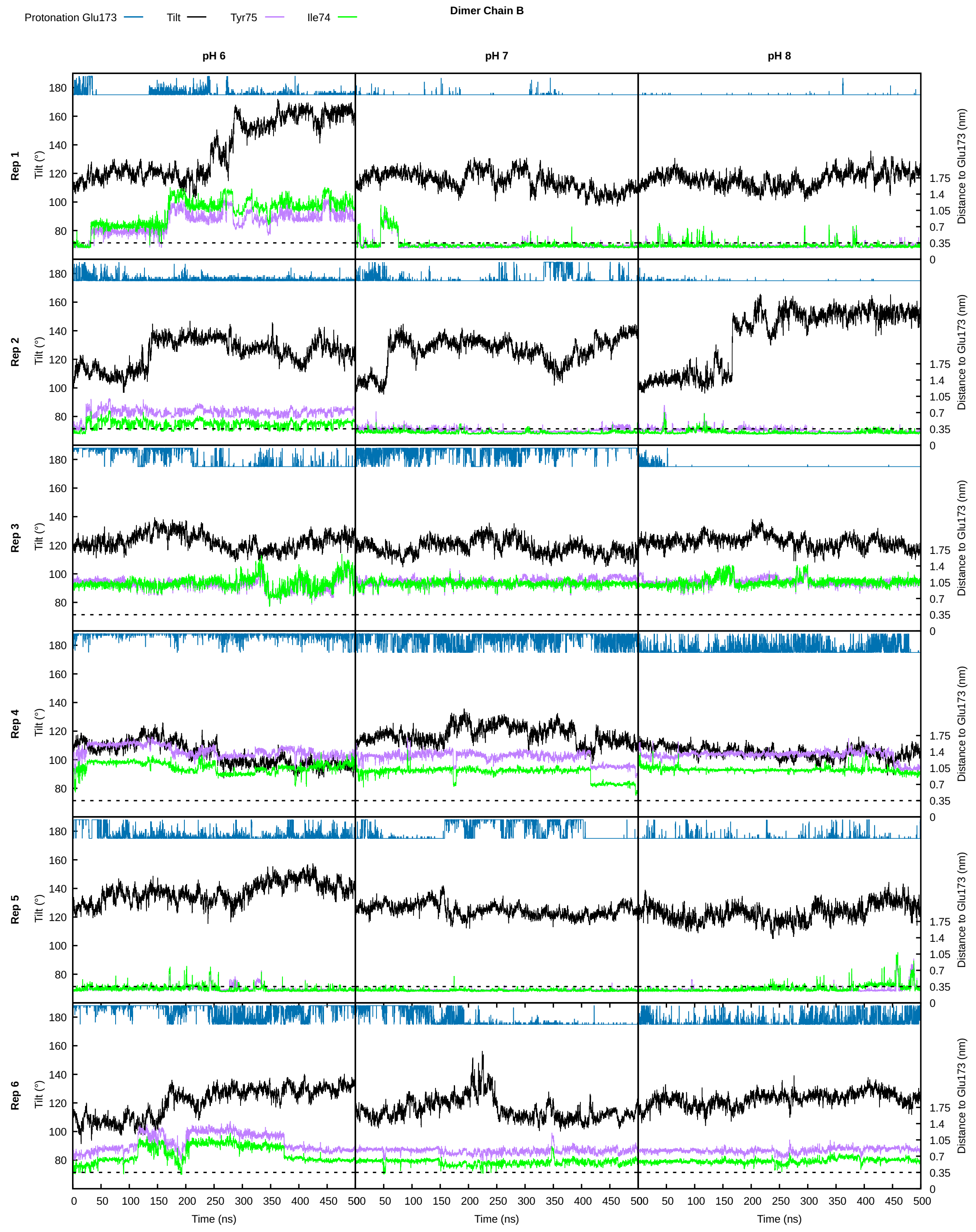

Figure S12: (continued)

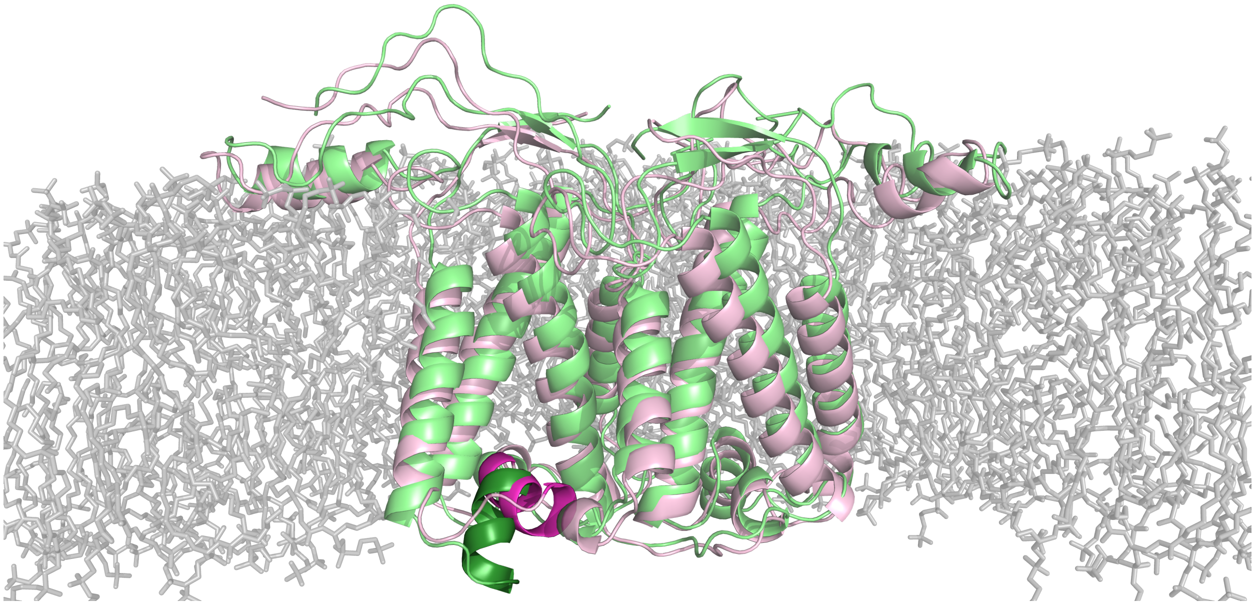

Figure S13: Illustration of the two adopted H2 positions of the PsbS dimer in the membrane: within the membrane (pink, lowest tilt) and in the aqueous environment (green, highest tilt).

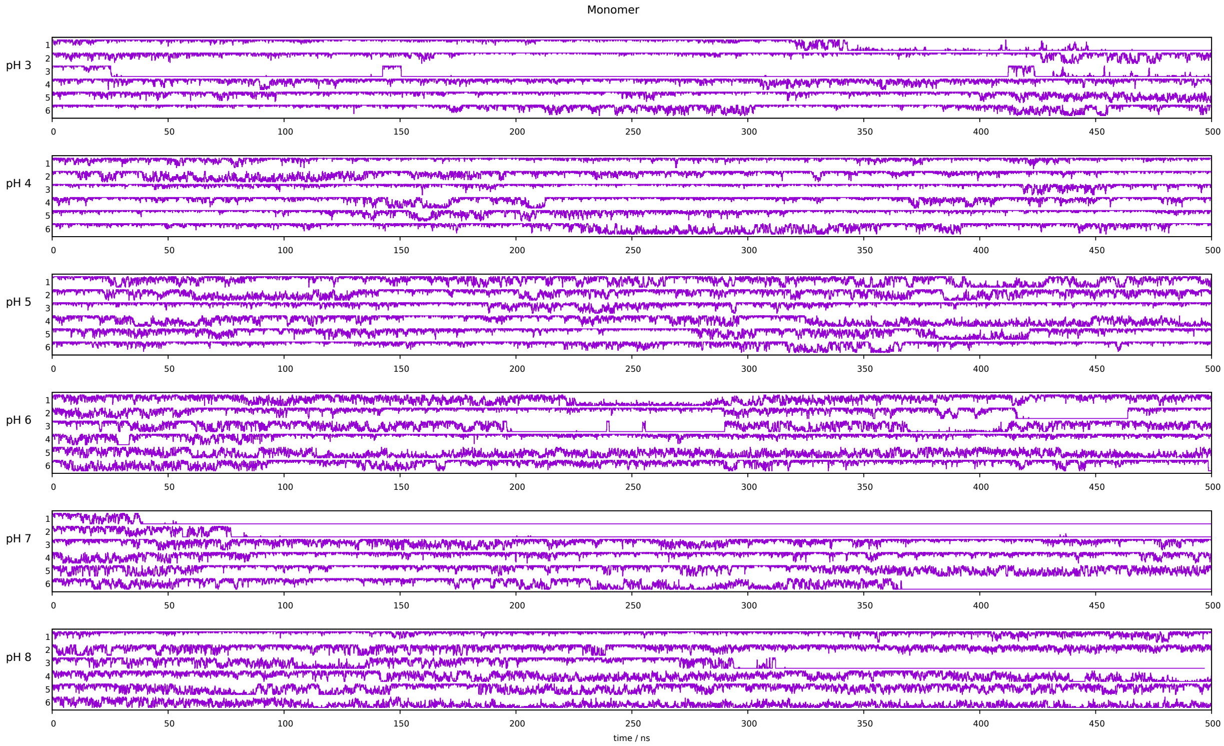

Figure S14: Block averages (0.1 ns windows) of the H3 helical transition for the monomer and chains A and B of the dimer, for all pH values. Each line corresponds to a replicate (numbered 1–6) and varies between 0 (disorded state) and 1 (3_{10} helix).

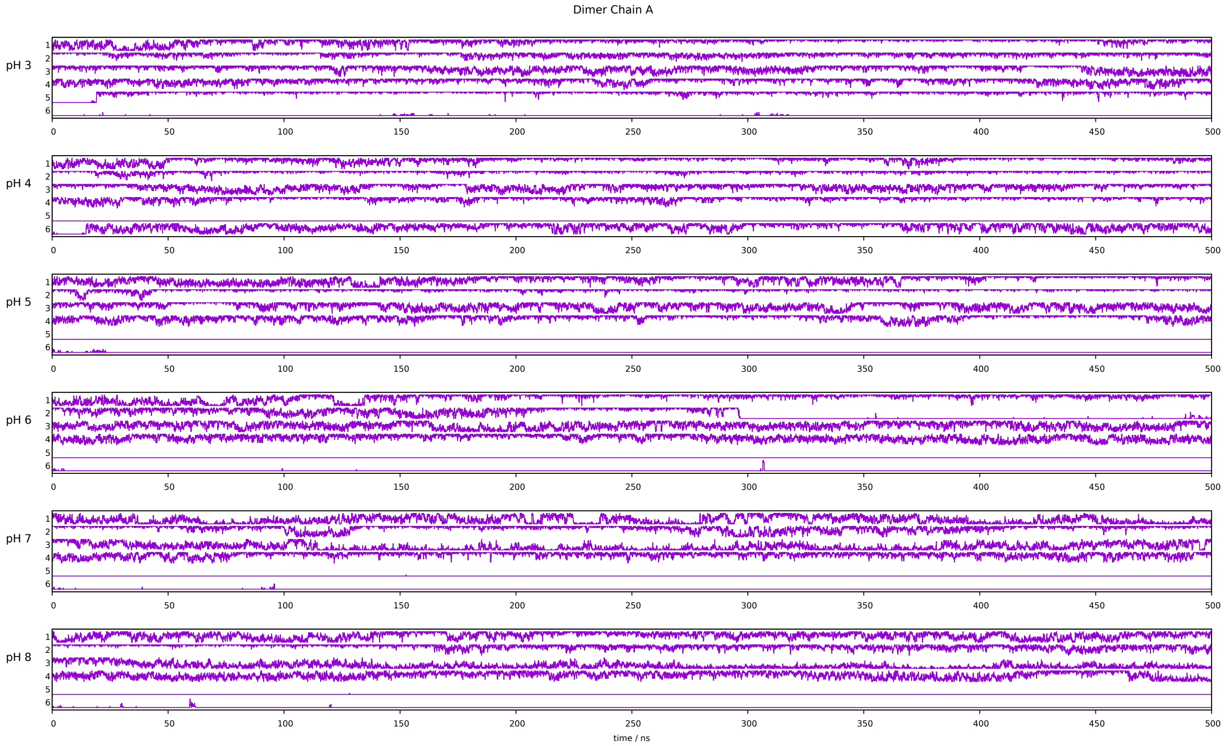

Figure S14: (continued, part 2)

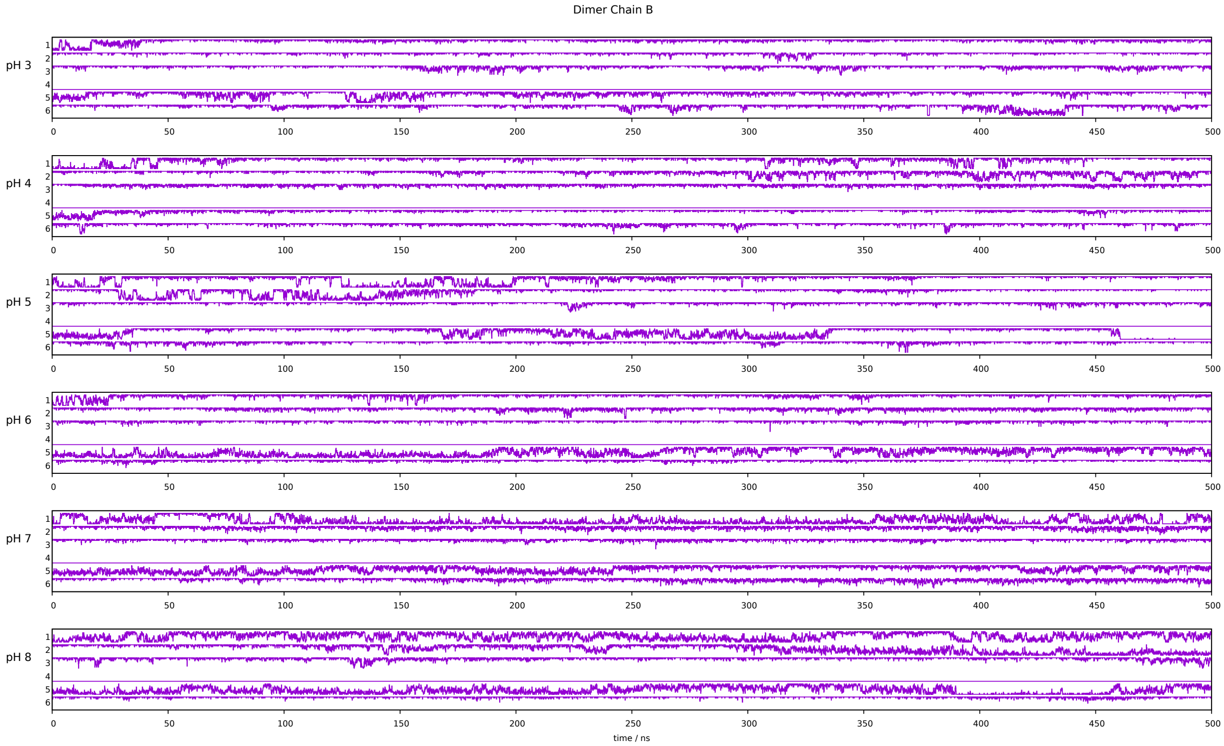

Figure S14: (continued, part 3)

4 Protonation Behaviour

Table S1: Comparison between our computed pK_a values in the monomer and dimer with bootstrap errors, including the corresponding ΔpK_a (monomer to dimer shift), and the computed pK_a values by Liguori et al.[S21] in the monomer and by Chiarrielo et al.[S37] in the dimer. Sites are labeled according to their localization, stroma (S) or lumen (L).

	**This work**			**Liguori et al.**	**Chiariello et al.**
**Site**	**Monomer**	**Dimer**	**ΔpK_a**	**Monomer**	**Dimer**
Glu13 - S	3.9 ± 0.2	3.3 ± 0.2	−0.6	3.9	4.4
Glu20 - S	5.2 ± 0.3	2.7 ± 0.6	−2.5	4.4	5.9
Asp21 - S	3.2 ± 0.5	1.4 ± 0.9	−2.2	3.7	–
Glu35 - S	3.8 ± 0.3	4.2 ± 0.1	0.4	4.1	5.7
Glu37 - S	5.8 ± 0.3	3.8 ± 0.5	−2.0	3.5	6.0
Glu55 - L	2.0 ± 74.9	4.0 ± 0.3	2.0	–	5.7
Glu69 - L	5.2 ± 0.2	4.6 ± 0.1	−0.6	5.2	6.7
Glu76 - L	5.5 ± 0.2	5.8 ± 0.1	0.3	5.7	5.7
Glu78 - L	6.7 ± 0.4	7.0 ± 0.2	0.3	6.3	7.0
Asp98 - S	3.5 ± 0.3	0.6 ± 0.7	−2.9	2.9	–
Asp104 - S	1.5 ± 1.5	2.1 ± 0.5	0.6	3.2	–
Glu105 - S	3.9 ± 0.2	3.5 ± 0.2	−0.4	3.4	4.7
Glu111 - S	4.1 ± 0.2	4.0 ± 0.1	−0.1	3.9	4.4
Asp120 - S	4.3 ± 0.3	4.0 ± 0.1	−0.3	4.5	–
Glu141 - S	6.1 ± 0.3	3.0 ± 0.5	−3.1	5.1	–
Glu159 - L	3.4 ± 2.2	2.0 ± 0.9	−1.4	4.1	5.7
Glu173 - L	6.1 ± 0.4	5.8 ± 0.2	−0.3	6.1	5.0
Glu180 - L	6.2 ± 0.2	6.4 ± 0.2	0.2	6.4	5.7
Glu182 - L	7.2 ± 0.3	6.9 ± 0.1	−0.3	7.7	5.4
Asp207 - S	2.7 ± 0.9	2.8 ± 0.1	0.1	3.8	–

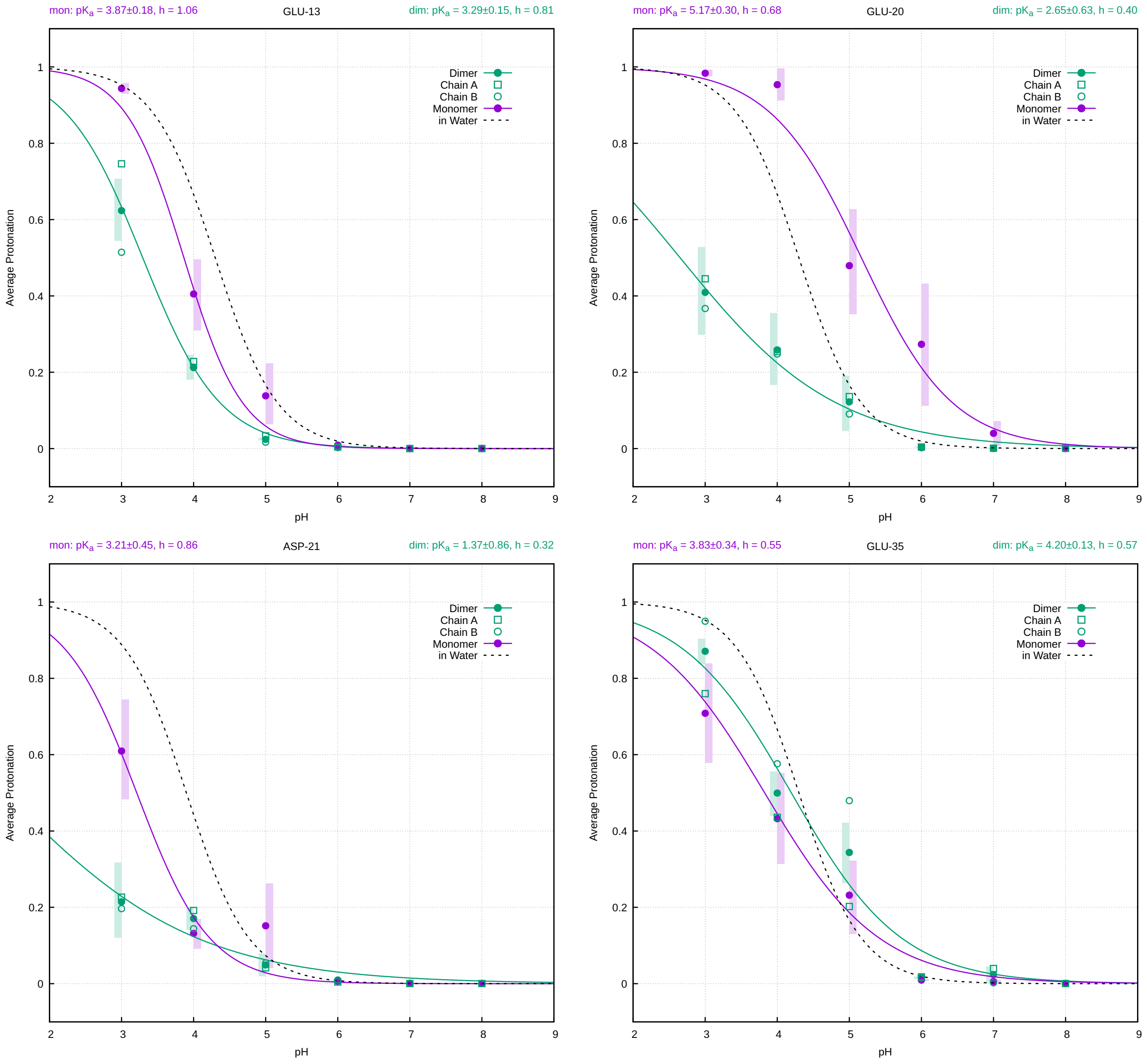

Figure S15: Titration curves of titratable residues in PsbS. The average protonation of each residue is shown for the monomer (purple) and dimer (green), with residues in water represented by black dashed curves. For the dimer, filled circles indicate the average over both chains, while chain A is shown as circles and chain B as squares. The titration curves were fitted to the Hill equation, and the fitted pK_a values (± error) and Hill coefficients (h) are indicated for each residue. The shaded transparent regions (purple and green) represent the error bounds, estimated as 68% confidence intervals obtained through the bootstrap method.

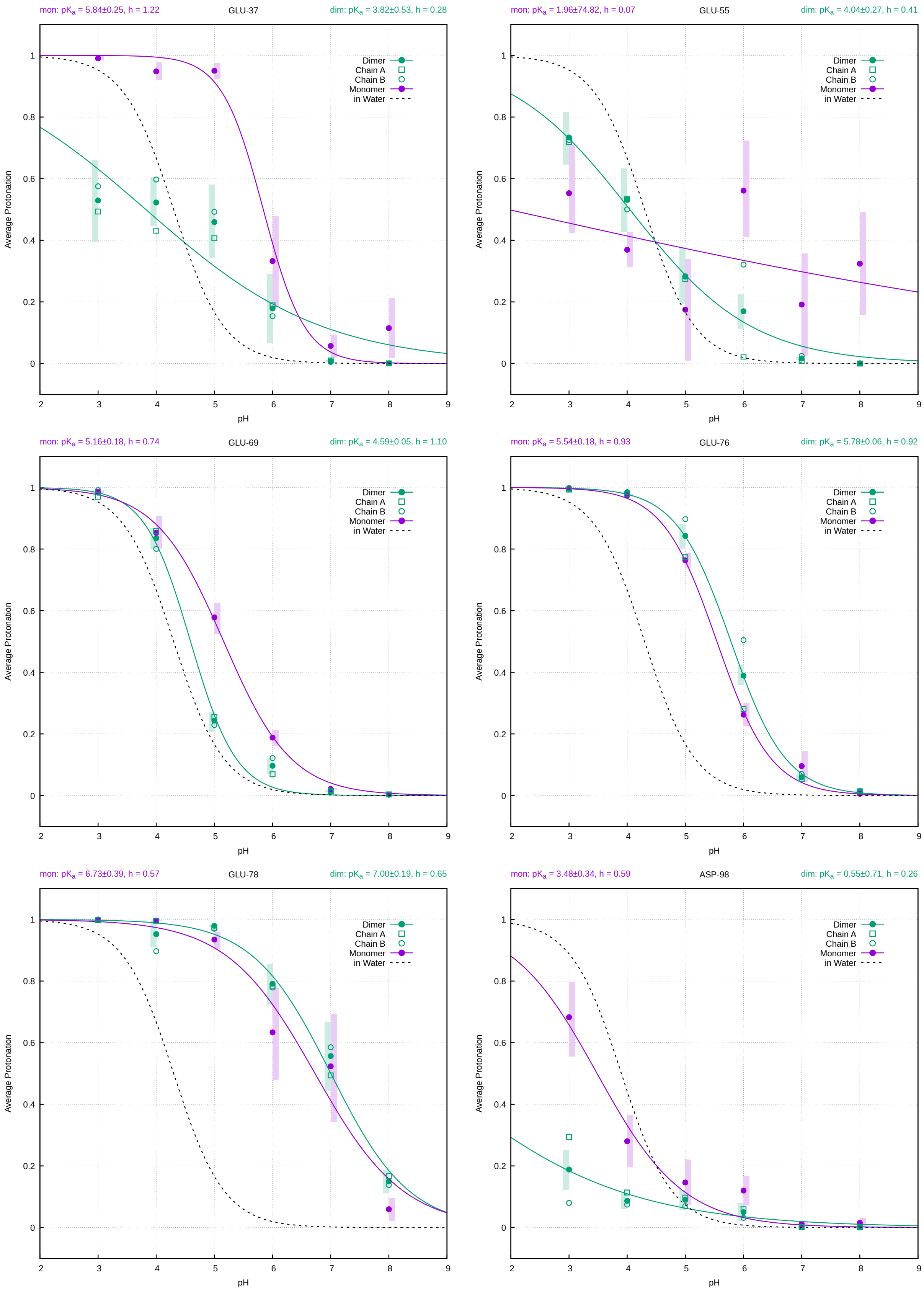

Figure S15: Titration curves of titratable residues in PsbS (continued.)

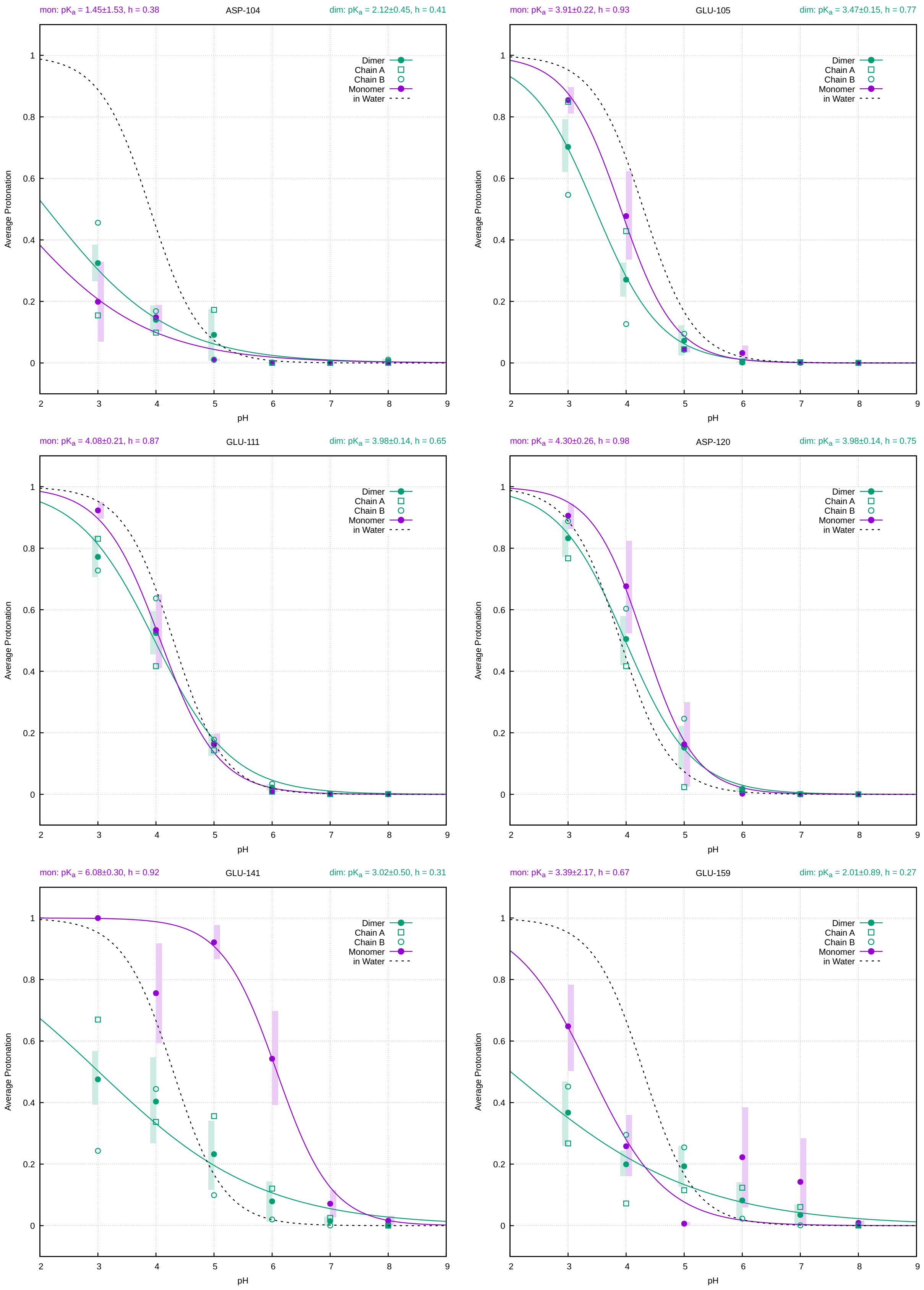

Figure S15: Titration curves of titratable residues in PsbS (continued.)

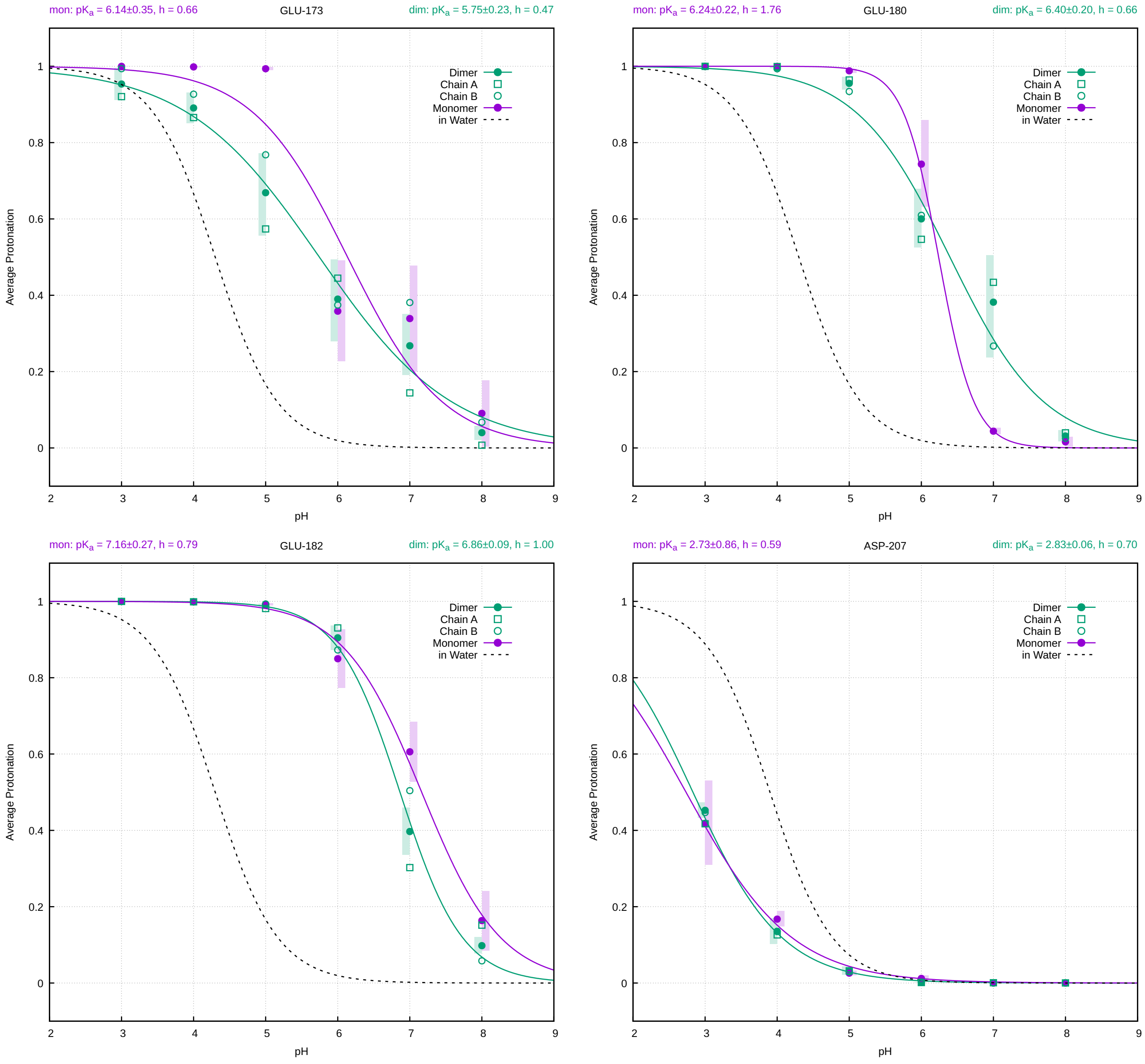

Figure S15: Titration curves of titratable residues in PsbS (continued.)

5 Protonation Correlations

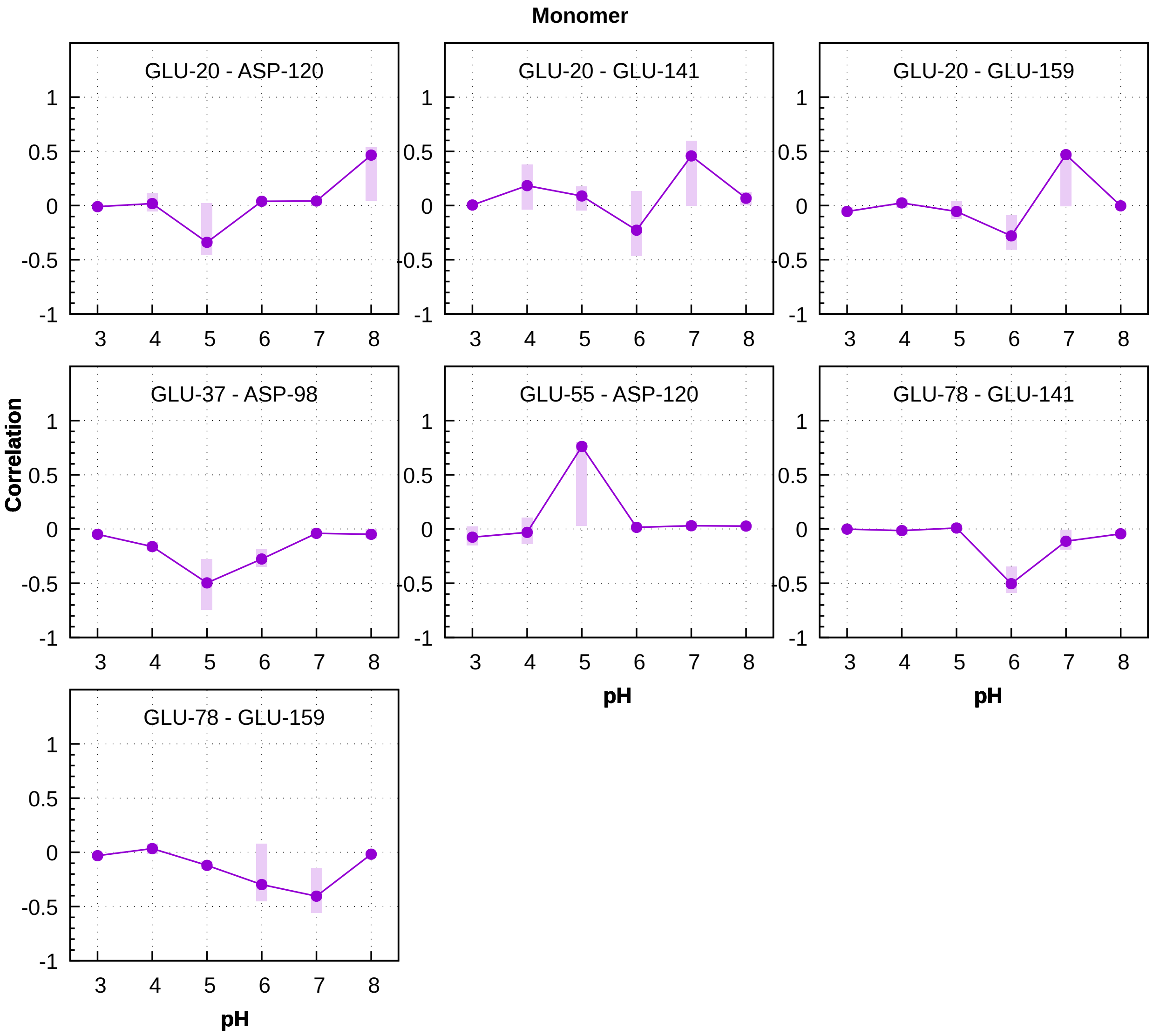

Figure S16: Correlation profiles of site pairs of the monomer that have at least one correlation coefficient with absolute value above 0.4. Each panel shows the correlation as a function of pH for the indicated pair. The shaded transparent purple regions represent the error bounds, estimated as 68% confidence intervals obtained through the bootstrap method.

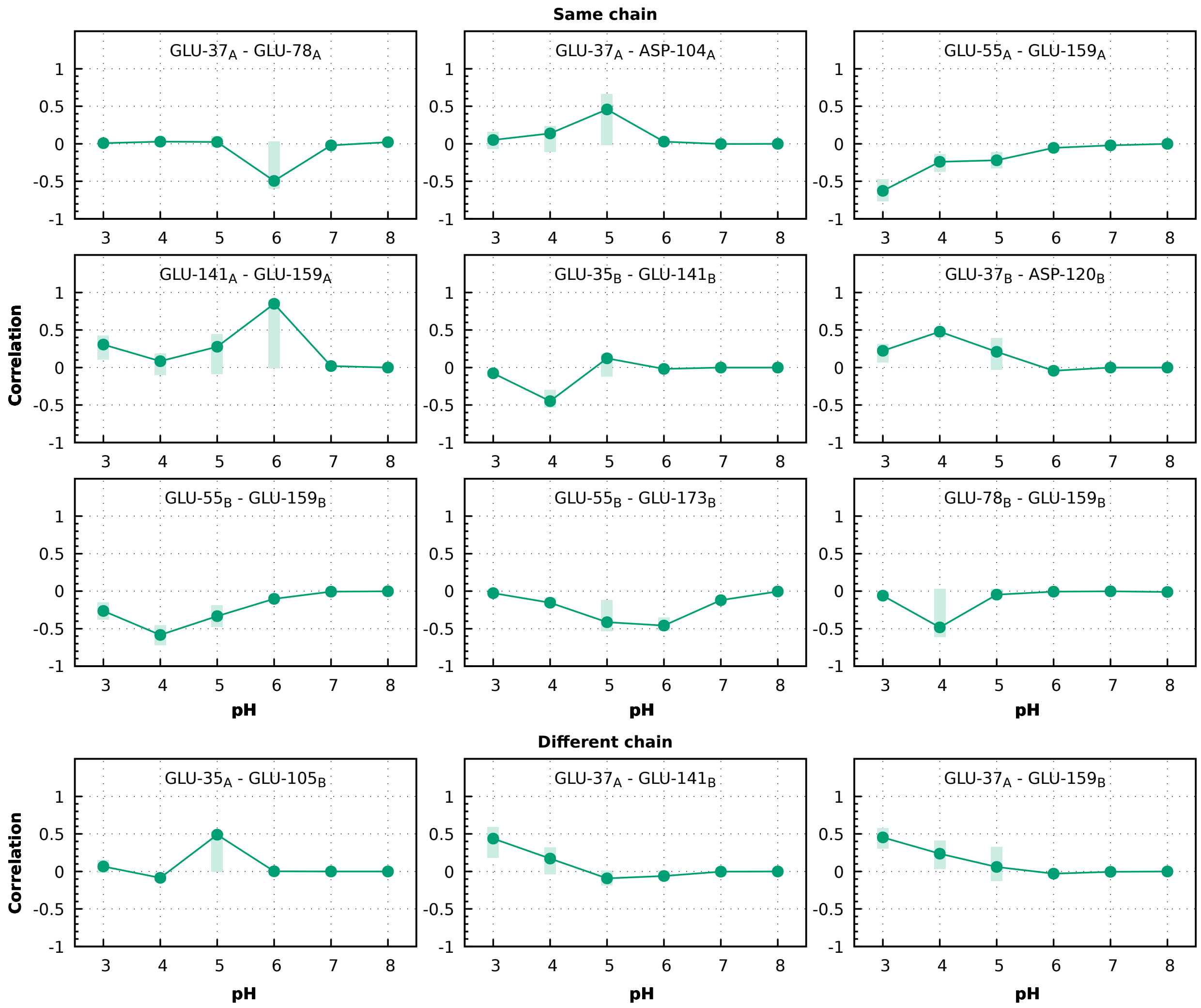

Figure S17: Correlation profiles of site pairs of the dimer that have at least one correlation coefficient with absolute value above 0.4. Each panel shows the correlation as a function of pH for the indicated pair. The shaded transparent green regions represent the error bounds, estimated as 68% confidence intervals obtained through the bootstrap method. Results are grouped according to whether the pairs belong to the same chain or to different chains.

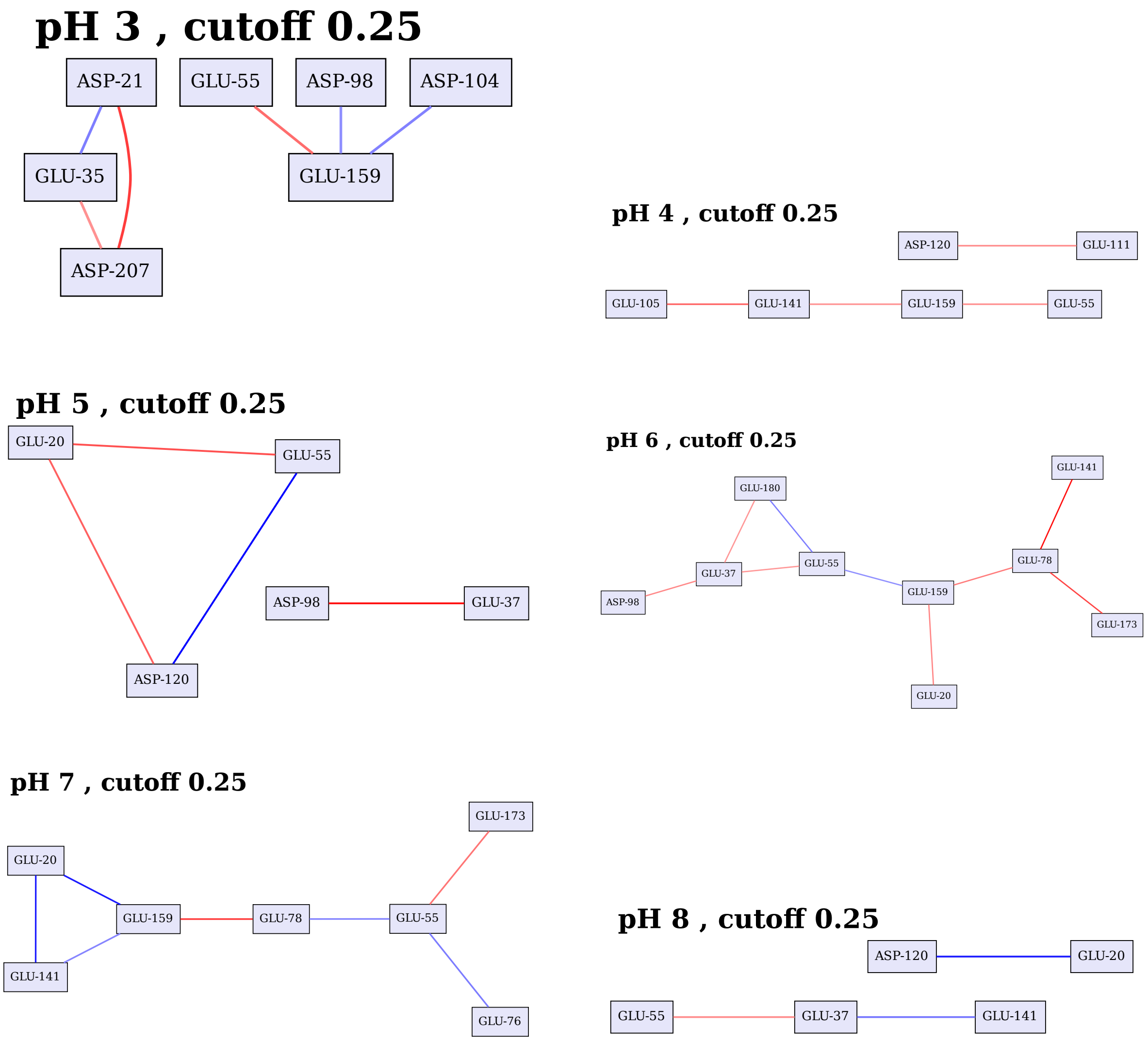

Figure S18: Correlation network between residue pairs of the monomer at pH 3–8 with a cutoff of 0.25.

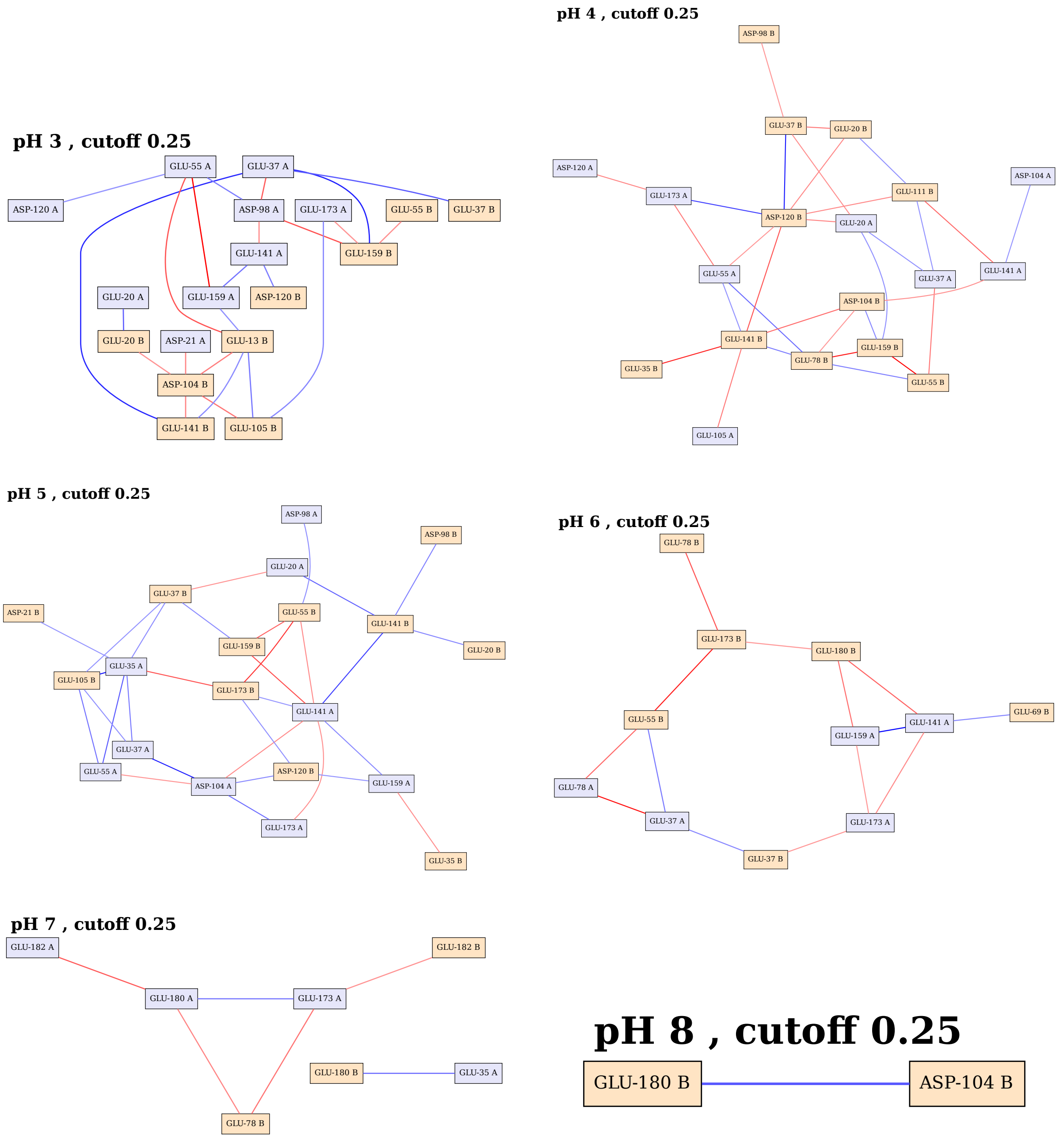

Figure S19: Correlation network between residue pairs of the dimer at pH 3–8 with a cutoff of 0.25.

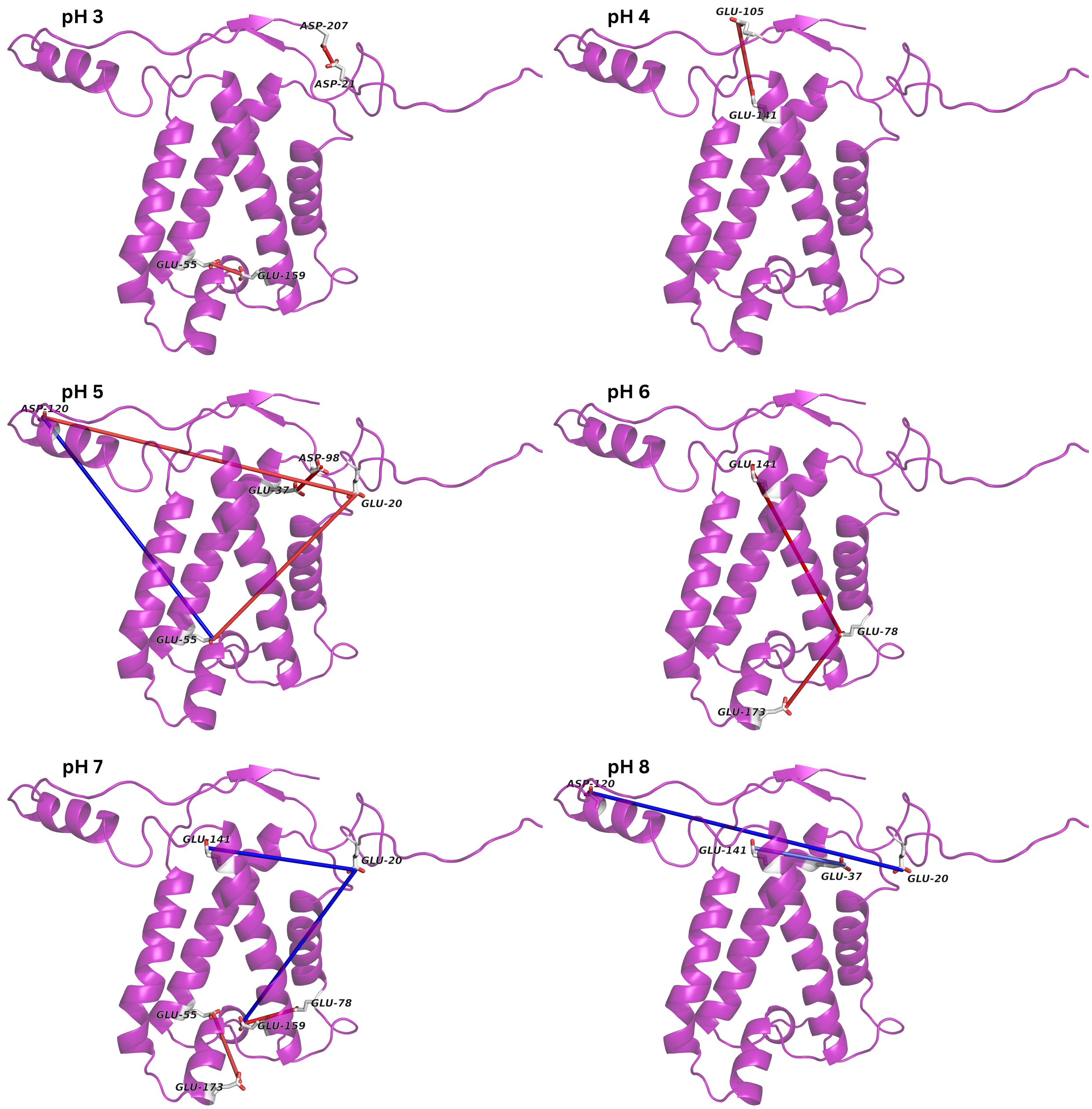

Figure S20: Correlated residue pairs in the PsbS monomer at pH 3–8. Residue pairs that have correlation coefficients with absolute value above 0.3 are shown as connecting lines: blue for positive correlations and red for negative correlations.

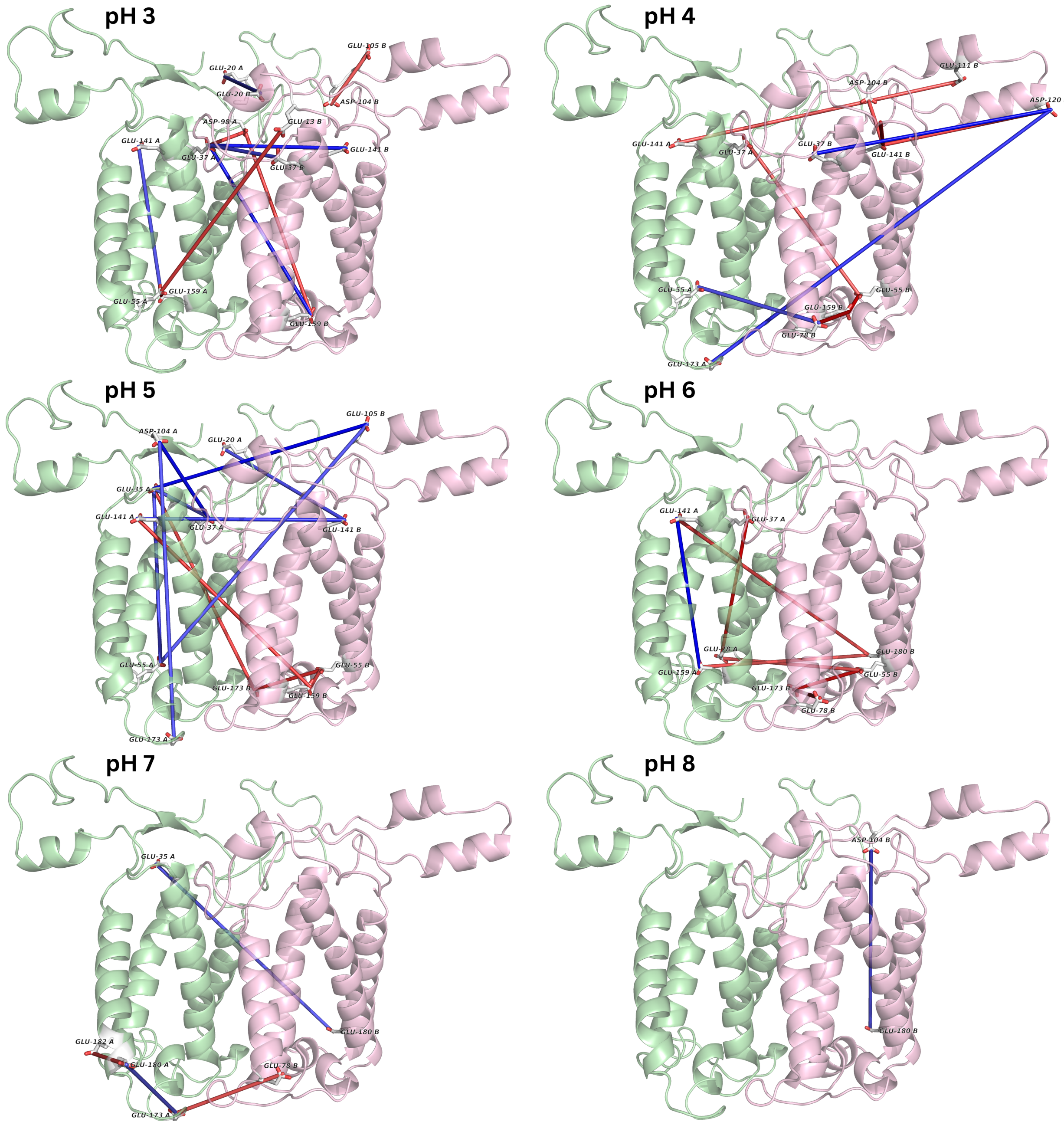

Figure S21: Correlated residue pairs in the PsbS dimer at pH 3–8. Residue pairs that have correlation coefficients with absolute value above 0.3 are shown as connecting lines: blue for positive correlations and red for negative correlations.

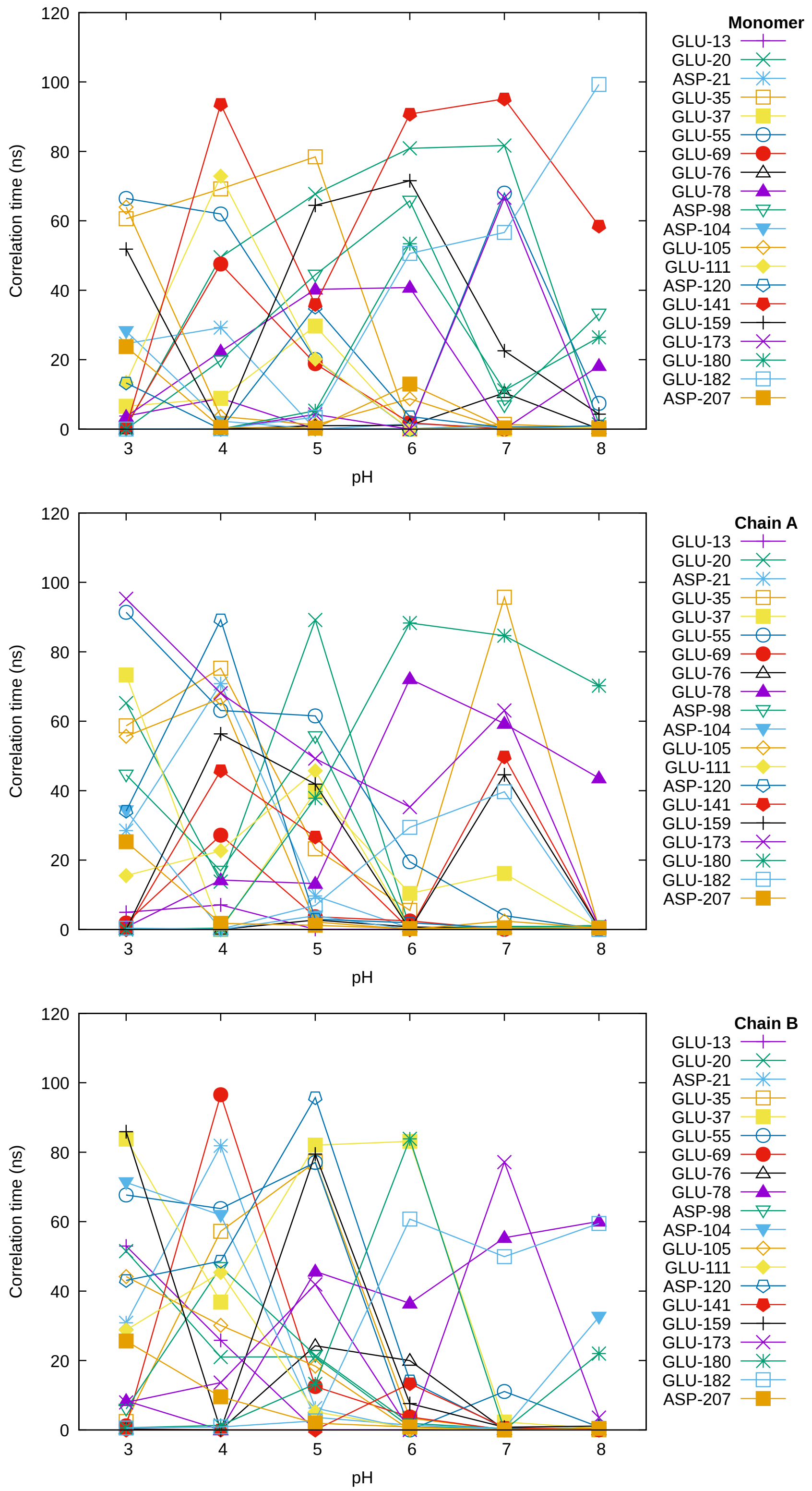

Figure S22: Correlation time of the proton occupancy (0 or 1) of the PsbS sites in the monomer and chains A and B from the dimer, as a function of pH. This was estimated as the time at which the protonation autocorrelation function becomes lower than 0.1.

References

(S1) Fan, M.; Li, M.; Liu, Z.; Cao, P.; Pan, X.; Zhang, H.; Zhao, X.; Zhang, J.; Chang, W. Crystal structures of the PsbS protein essential for photoprotection in plants. *Nat. Struct. Mol. Biol.* **2015**, *22*, 729–735.

(S2) Bank, R. P. D. RCSB PDB - 4RI2: Crystal structure of the photoprotective protein PsbS from spinach — rcsb.org. https://www.rcsb.org/structure/4ri2, [Accessed 17-01-2025].

(S3) Schwede, T. SWISS-MODEL: an automated protein homology-modeling server. *Nucleic Acids Res.* **2003**, *31*, 3381–3385.

(S4) Humphrey, W.; Dalke, A.; Schulten, K. VMD: Visual molecular dynamics. *J. Mol. Graphics* **1996**, *14*, 33–38.

(S5) Schmid, N.; Eichenberger, A. P.; Choutko, A.; Riniker, S.; Winger, M.; Mark, A. E.; van Gunsteren, W. F. Definition and Testing of the GROMOS Force-Field Versions 54A7 and 54B7. *Eur. Biophys. J.* **2011**, *40*, 843–856.

(S6) Hermans, J.; Berendsen, H. J. C.; van Gunsteren, W. F.; Postma, J. P. M. A Consistent Empirical Potential for Water–Protein Interactions. *Biopolymers* **1984**, *23*, 1513–1518.

(S7) Kukol, A. Lipid Models for United-Atom Molecular Dynamics Simulations of Proteins. *J. Chem. Theory Comput.* **2009**, *5*, 615–626.

(S8) Hess, B.; Bekker, H.; Berendsen, H. J. C.; Fraaije, J. G. E. M. LINCS: A Linear Constraint Solver for Molecular Simulations. *J. Comput. Chem.* **1997**, *18*, 1463–1472.

(S9) Darden, T.; York, D.; Pedersen, L. Particle mesh Ewald: An $N \cdot \log(N)$ method for Ewald sums in large systems. *J. Chem. Phys.* **1993**, *98*, 10089–10092.

(S10) Parrinello, M.; Rahman, A. Polymorphic transitions in single crystals: A new molecular dynamics method. *J. Appl. Phys.* **1981**, *52*, 7182–7190.

(S11) Nosé, S. A unified formulation of the constant temperature molecular dynamics methods. *J. Chem. Phys.* **1984**, *81*, 511–519.

(S12) da Rocha, L.; Baptista, A. M.; Campos, S. R. R. Approach to Study pH-Dependent Protein Association Using Constant-pH Molecular Dynamics: Application to the Dimerization of β-Lactoglobulin. *J. Chem. Theory Comput.* **2022**, *18*, 1982–2001.

(S13) Abraham, M. J.; Murtola, T.; Schulz, R.; Páll, S.; Smith, J. C.; Hess, B.; Lindahl, E. GROMACS: High performance molecular simulations through multi-level parallelism from laptops to supercomputers. *SoftwareX* **2015**, *1–2*, 19–25.

(S14) Miyamoto, S.; Kollman, P. A. SETTLE: An Analytical Version of the SHAKE and RATTLE Algorithms for Rigid Water Models. *J. Comput. Chem.* **1992**, *13*, 952–962.

(S15) Verlet, L. Computer "Experiments" on Classical Fluids. I. Thermodynamical Properties of Lennard-Jones Molecules. *Phys. Rev.* **1967**, *159*, 98–103.

(S16) Van Gunsteren, W. F.; Berendsen, H. J. C. A Leap-frog Algorithm for Stochastic Dynamics. *Mol. Simul.* **1988**, *1*, 173–185.

(S17) Berendsen, H. J. C.; Postma, J. P. M.; van Gunsteren, W. F.; DiNola, A.; Haak, J. R. Molecular dynamics with coupling to an external bath. *J. Chem. Phys.* **1984**, *81*, 3684–3690.

(S18) Baptista, A. M.; Teixeira, V. H.; Soares, C. M. Constant-pH molecular dynamics using stochastic titration. *J. Chem. Phys.* **2002**, *117*, 4184–4200.

(S19) Machuqueiro, M.; Baptista, A. M. Constant-pH Molecular Dynamics with Ionic Strength Effects: Protonation-Conformation Coupling in Decalysine. *J. Phys. Chem. B* **2006**, *110*, 2927–2933.

(S20) Bussi, G.; Donadio, D.; Parrinello, M. Canonical sampling through velocity rescaling. *J. Chem. Phys.* **2007**, *126*, 014101.

(S21) Liguori, N.; Campos, S. R. R.; Baptista, A. M.; Croce, R. Molecular Anatomy of Plant Photoprotective Switches: The Sensitivity of PsbS to the Environment, Residue by Residue. *J. Phys. Chem. Lett.* **2019**, *10*, 1737–1742.

(S22) Baptista, A. M.; Soares, C. M. Some Theoretical and Computational Aspects of the Inclusion of Proton Isomerism in the Protonation Equilibrium of Proteins. *J. Phys. Chem. B* **2001**, *105*, 293–309.

(S23) Carvalheda, C. A.; Campos, S. R. R.; Machuqueiro, M.; Baptista, A. M. Structural Effects of pH and Deacylation on Surfactant Protein C in an Organic Solvent Mixture: A Constant-pH MD Study. *J. Chem. Inf. Model.* **2013**, *53*, 2979–2989.

(S24) Grimsley, G. R.; Scholtz, J. M.; Pace, C. N. A summary of the measured pK values of the ionizable groups in folded proteins. *Protein Sci.* **2009**, *18*, 247–251.

(S25) Bashford, D.; Gerwert, K. Electrostatic calculations of the pK_a values of ionizable groups in bacteriorhodopsin. *J. Mol. Biol.* **1992**, *224*, 473–486.

(S26) Teixeira, V. H.; Cunha, C. A.; Machuqueiro, M.; Oliveira, A. S. F.; Victor, B. L.; Soares, C. M.; Baptista, A. M. On the Use of Different Dielectric Constants for Computing Individual and Pairwise Terms in Poisson–Boltzmann Studies of Protein Ionization Equilibrium. *J. Phys. Chem. B* **2005**, *109*, 14691–14706.

(S27) Metropolis, N.; Rosenbluth, A. W.; Rosenbluth, M. N.; Teller, A. H.; Teller, E. Equation of State Calculations by Fast Computing Machines. *J. Chem. Phys.* **1953**, *21*, 1087–1092.

(S28) Wyman, J.; Gill, S. J. *Binding and Linkage: Functional Chemistry of Biological Macromolecules*; University Science Books: Mill Valley, CA, 1990.

(S29) Tanford, C. *Advances in Protein Chemistry*; Elsevier, 1970; Vol. 24; pp 1–95.

(S30) Knott, G. D. *Interpolating Cubic Splines*; Springer Science & Business Media: New York, 2000; Vol. 18.

(S31) Lauffenburger, D. A.; Linderman, J. J. *Receptors: Models for Binding, Trafficking, and Signaling*; Oxford University Press, 1996.

(S32) Machuqueiro, M.; Baptista, A. M. Acidic range titration of HEWL using a constant-pH molecular dynamics method. *Proteins: Struct., Funct., Bioinf.* **2008**, *72*, 289–298.

(S33) Williams, T.; Kelley, C.; many others, Gnuplot 5.2: an interactive plotting program. 2021; Available at http://gnuplot.sourceforge.net/.

(S34) Baptista, A. M.; Martel, P. J.; Soares, C. M. Simulation of Electron-Proton Coupling with a Monte Carlo Method: Application to Cytochrome c_3 Using Continuum Electrostatics. *Biophys. J.* **1999**, *76*, 2978–2998.

(S35) Carvalheda, C. A.; Campos, S. R. R.; Baptista, A. M. The Effect of Membrane Environment on Surfactant Protein C Stability Studied by Constant-pH Molecular Dynamics. *J. Chem. Inf. Model.* **2015**, *55*, 2206–2217.

(S36) DeLano, W. L. The PyMOL Molecular Graphics System. DeLano Scientific: San Carlos, CA, USA, 2002; Available at http://www.pymol.org.

(S37) Chiariello, M. G.; Grünewald, F.; Zarmiento-Garcia, R.; Marrink, S. J. pH-Dependent Conformational Switch Impacts Stability of the PsbS Dimer. *J. Phys. Chem. Lett.* **2023**, *14*, 905–911.
